# Dynamic expression of long noncoding RNAs and repeat elements in synaptic plasticity

**DOI:** 10.3389/fnins.2015.00351

**Published:** 2015-10-01

**Authors:** Jesper L. V. Maag, Debabrata Panja, Ida Sporild, Sudarshan Patil, Dominik C. Kaczorowski, Clive R. Bramham, Marcel E. Dinger, Karin Wibrand

**Affiliations:** ^1^Genomics and Epigenetics Division, Garvan Institute of Medical ResearchSydney, NSW, Australia; ^2^Faculty of Medicine, St Vincent's Clinical School, University of New South WalesSydney, NSW, Australia; ^3^Department of Biomedicine and K.G. Jebsen Centre for Research on Neuropsychiatric Disorders, University of BergenBergen, Norway

**Keywords:** long noncoding RNA (lncRNA), LTP (long term potentiation), retrotransposons, repeat elements, rat brain, time-series data, synaptic plasticity (LTP/LTD)

## Abstract

Long-term potentiation (LTP) of synaptic transmission is recognized as a cellular mechanism for learning and memory storage. Although de novo gene transcription is known to be required in the formation of stable LTP, the molecular mechanisms underlying synaptic plasticity remain elusive. Noncoding RNAs have emerged as major regulatory molecules that are abundantly and specifically expressed in the mammalian brain. By combining RNA-seq analysis with LTP induction in the dentate gyrus of live rats, we provide the first global transcriptomic analysis of synaptic plasticity in the adult brain. Expression profiles of mRNAs and long noncoding RNAs (lncRNAs) were obtained at 30 min, 2 and 5 h after high-frequency stimulation of the perforant pathway. The temporal analysis revealed dynamic expression profiles of lncRNAs with many positively, and highly, correlated to protein-coding genes with known roles in synaptic plasticity, suggesting their possible involvement in LTP. In light of observations suggesting a role for retrotransposons in brain function, we examined the expression of various classes of repeat elements. Our analysis identifies dynamic regulation of LINE1 and SINE retrotransposons, and extensive regulation of tRNA. These experiments reveal a hitherto unknown complexity of gene expression in long-term synaptic plasticity involving the dynamic regulation of lncRNAs and repeat elements. These findings provide a broader foundation for elucidating the transcriptional and epigenetic regulation of synaptic plasticity in both the healthy brain and in neurodegenerative and neuropsychiatric disorders.

## Introduction

Long-lasting changes in synaptic communication are thought to underlie memory storage and adaptive functions of the brain related to fear, anxiety and reward (Malenka and Bear, 2004; Whitlock, 2006; Nabavi et al., 2014; Baudry et al., 2015). Long-term potentiation (LTP), a persistent increase in synaptic strength induced by electrical stimulation of synapses, is a widely used model for the cellular and molecular analysis of synaptic plasticity (Bliss and Lomo, 1973). Stable forms of activity-dependent synaptic plasticity require coordinated gene transcription and protein synthesis as well as protein degradation (Sutton and Schuman, 2006; Bramham and Wells, 2007). Although frequently addressed, the complex molecular network underlying long-lasting synaptic changes is not well understood.

One particular aspect of synaptic plasticity that has seldom been explored is the role of noncoding regions of the genome and the potential regulatory functions that they contain. The argument that these noncoding regions may be important in brain function arises predominantly from the observation that organisms with increasingly complex brains have genomes comprising increasingly large proportions of noncoding DNA. In contrast, the frequency of protein-coding genes remains relatively constant across large evolutionary distances, despite dramatic changes in organismal complexity (Liu et al., 2013). One means through which noncoding DNA transacts function is through expression into regulatory RNAs, which have been speculated upon previously to function in long-term memory formation (Mercer et al., 2007). Whole transcriptome sequencing studies have revealed that the majority of mammalian genomes are pervasively transcribed (Cheng et al., 2005; Kapranov et al., 2007; Djebali et al., 2013). However, the function of the vast majority of these transcripts is unknown and remains an area of some controversy (Clark et al., 2011).

One of the major outputs of pervasive transcription are long noncoding RNAs (lncRNAs), which are annotated according to their length (exceeding 200 nucleotides) and lack of a discernible open reading frame (Dinger et al., 2008b). LncRNAs have emerged as important regulators of transcription at different levels (Cesana et al., 2011; Clark and Blackshaw, 2014) including imprinting (Latos et al., 2012), epigenetic regulation of chromatin structure (Mercer and Mattick, 2013), transcription factor interaction (Wang et al., 2014), post-translational interaction with mRNA (Kretz et al., 2012), and decoy of microRNAs (Cesana et al., 2011). Functionally, several lncRNAs have been linked to organ development, cell pluripotency and differentiation (Dinger et al., 2008b; Fatica and Bozzoni, 2013), with studies mainly carried out using human induced pluripotent stem cells (iPSC) (Loewer et al., 2010), mouse- (Dinger et al., 2008a), and human embryonic stem cells (ESC) (Ng et al., 2011). Interestingly, a great number of lncRNAs show enrichment in the central nervous system and many are both tissue and cell specific (Mercer et al., 2008). Following from this observation, lncRNAs have been shown to regulate brain development *in vivo* (Bond et al., 2009; Sauvageau et al., 2013) and synaptogenesis *in vitro* (Bernard et al., 2010; Clark and Blackshaw, 2014). However, it is unknown whether lncRNA expression is regulated in the context of activity-dependent synaptic plasticity.

Another recently described noncoding element that may play a role in synaptic plasticity is transposable elements. Transposable elements (TEs), which comprise 66% of the human genome (de Koning et al., 2011), are mobile sequences that can contribute to genomic instability and modify gene expression networks both in the germline and somatic cells. Recent studies have shown that TEs are involved in creating genetic heterogeneity in the CNS, moreover these insertions are enriched in hippocampal genes and neuronal stem cell enhancers (Thomas and Muotri, 2012; Upton et al., 2015). In addition, they display altered expression in the adult CNS (Reilly et al., 2013) in processes such as neurogenesis (Muotri et al., 2005, 2010; Coufal et al., 2009), aging (Li et al., 2013), neurodegenerative diseases (Li et al., 2012), alcoholism (Ponomarev et al., 2012), and post-traumatic stress disorder (PTSD) (Ponomarev et al., 2010). Little is known about how TE mobility is regulated and the role of TEs in synaptic plasticity has not been explored.

Previous studies have revealed dynamic regulation of mRNA and microRNA expression in long-term synaptic plasticity (Håvik et al., 2007; Ryan et al., 2011; Wibrand et al., 2012; Joilin et al., 2014; Pai et al., 2014). Here, we report the first global transcriptome analysis of *in vivo* synaptic plasticity, using the well-established model of LTP in the rat dentate gyrus (DG). The dentate gyrus (DG), a subregion of the hippocampus, is involved in processing information that results in declarative memory formation. Using RNA-seq we have identified a number of novel lncRNAs that are dynamically regulated in response to LTP. In addition, we also observed an altered expression of multiple classes of repeat elements including retrotransposons. Taken together, the identification of dynamic expression of these groups of noncoding RNAs in response to synaptic activation opens new avenues for future studies into the mechanisms surrounding synaptic plasticity, memory formation, and human cognitive diseases.

## Materials and methods

### Animals

*In vivo* electrophysiological experiments were carried out on 20 adult male rats of the Sprague–Dawley outbred strain (Taconic Europe, Ejby, Denmark), weighing 250–350 g while 3 animals were used in the naïve untreated group. They had free access to food and water and were on a 12-h light/dark cycle. Three animals per group were chosen for RNA-sequencing. Animal experiments were carried out in accordance with the European Community Council Directive of 24 November 1986 (86/609/EEC) and approved by the Norwegian Committee for Animal Research.

### Electrophysiology

The electrophysiology procedures have been detailed elsewhere (Messaoudi et al., 2002; Panja et al., 2009). Briefly, rats were anesthetized with urethane and electrodes were inserted for selective stimulation the medial perforant pathway and recording of evoked potentials in the hilus region of the dentate gyrus. Recordings were done with borosilicate glass micropipettes (tip size 4–8 μm) filled with 1 M NaCl (input impedance 3–4 MΩ). Test pulses were applied at 0.033 Hz thought the experiment except during the period of HFS. The HFS paradigm for LTP induction consisted of eight pulses at 400 Hz, repeated four times, at 10 s intervals. Three sessions of HFS were given, with 5 min between each HFS. The baseline stimulated group received test pulses (0.033 Hz) only without the HFS. CPP [(R,S)-3-22-carboxypiperazin-4-yl-propyl-1-phosphonic acid; Tocris, #0173] was dissolved in saline and injected IP at a dose of 10 mg/kg, 90 min prior to HFS. Signals from the dentate hilus were amplified, filtered (1 Hz–10 kHz), and digitized (25 kHz). Acquisition and analysis of field potentials were accomplished using Data Wave Technologies Work Bench Software (Longmont, CO). The maximum slope of the fEPSP and the amplitude of the population spike measured from its negative going apex to the tangent line, joining the first two positive peaks were measured and the averages of four consecutive responses were obtained.

### Dissection and tissue homogenization

Animals were decapitated at indicated time points after HFS induction and the dentate gyrus (DG) and the cornu amonis (CA) of the hippocampus were rapidly micro dissected on ice. After dissection, the tissue was flash frozen on dry ice and stored at −80°C until use. The tissues were homogenized (6500 rpm, 2 cycles at 15 s) in 600 μl cold lysis buffer (AllPrep DNA/RNA Mini Kit, Qiagen) or in 500 μl Trizol, using the Prelyse system (Prelyse™ 24, Bertin Technologies, Montigny-le-Bretonneux, France) and recommended ceramic beads (03961ck14).

### RNA extraction

The RNA for *RNA-seq experiment II* was isolated using Allprep RNA/DNA isolation kit (Qiagen) with on-column DNase treatment. RNA from primary hippocampal cell cultures was isolated with Trizol, dissolved in nuclease free water and treated with Turbo DNase (Ambion) to remove genomic DNA. Trizol purified RNA was finally precipitated and dissolved in 1 mM EDTA. RNA samples were evaluated by ultraviolet spectroscopy for purity and concentration (NanoDrop 2000 Spectrophotometer, Thermo Scientific) and were assessed further for RNA integrity on the Agilent 2100 Bioanalyzer (Agilent Technologies). All analyzed samples had an RNA Integrity Number (RIN) of 8 or better.

### RNA sequencing

Total RNA was stabilized with RNAstable® (Biomatrica, San Diego, CA, USA) and sent to the Garvan Institute of Medical Research, Sydney, Australia) for RNA library construction and subsequent RNA sequencing. RNA stabilized with RNAstable® was resuspended with nuclease-free H_2_O. The 4 experimental treatment groups were 30 min LTP, 2 h LTP, 5 h LTP, and naïve tissue (3 biological replicates for experimental and control dentate gyrus). RNA quality was assessed with an Agilent Technologies 2100 Bioanalyzer with an RNA 6000 Nano kit (Agilent Technologies, USA) according to the manufacturer's instructions. RNA concentration was measured using a Nanodrop 2000 spectrophotometer (Thermo Fisher Scientific, USA). 500 ng of total RNA was used as input material for library preparation using the TruSeq Stranded Total RNA Sample Prep Kit (LT) (Illumina, USA) according to manufacturer's instructions. Individual libraries were indexed as recommended by Illumina. Indexed cDNA libraries were analyzed individually using an Agilent Technologies 2100 Bioanalyzer with the DNA 1000 kit according to the manufacturer's instructions (Agilent Technologies, USA). Libraries were diluted and pooled to a final concentration of 10 nM each in nuclease-free H_2_O (Ambion, USA). Pooled libraries were quantitated using a Life Technologies Qubit 2.0 Fluorometer with the Qubit dsDNA HS Assay Kit (Life Technologies, USA) and further diluted to 2 nM. Final DNA library concentration was confirmed using a Qubit dsDNA HS Assay Kit. PCR-competent library DNA concentration was verified using the universal KAPA Library Quantification Kit for Illumina Sequencing Platforms according to manufacturer's instructions (KAPA Biosystems, USA). An Applied Biosystems 7900 HT Fast Real-Time PCR machine (Life Technologies, USA) was used for qPCR. For total RNA, sequencing was performed using the Illumina HiSeq 2500 platform with 100 bp paired-end sequencing with a fragment size of approximately 297 bp. Illumina TruSeq version 3 chemistry was used for cluster generation and sequencing.

### cDNA synthesis and semi-quantitative real-time PCR

300 ng of total RNA was used for cDNA synthesis using qScript™ cDNA SuperMix (Quanta Biosciences, Inc., Gaithersburg, MD) following the manufacturer's instructions. The cDNA was diluted 10 times in RNAse and DNAse free water prior to real-time PCR. Semiquantitative real-time PCR was performed on a Roche LightCycler® 480. 10 μl reactions were run in 384-well plates using gene specific primers (Table S1) and 2x PerfecCTa™ SYBR® Green FastMix™ (Quanta BioScience,) at 60°C. Samples were run in triplicates. The geometric mean of Cyclopholin, Hypoxanthine-guanine phosphoribosyltransferase and Polyubiqutin was used for normalization. The relative amount of Arc mRNA, Ptgs bidirectional RNA and SINEs were calculated in treated vs. untreated samples by the second derivative method. Primer efficiency for all genes analyzed was determined by standard curve.

### Trimming and mapping of reads to the rat genome

The reads were analyzed with FastQC to find inherent biases in the sequencing. The reads were then trimmed using trimgalore (http://www.bioinformatics.babraham.ac.uk/projects/trim_galore/) on default settings, and quality inspected with FastQC again to assert the effect of trimming on the read quality. Alignment to the Rn4 Rattus_norvegicus.RGSC3.4.69.dna.toplevel.fa genome was done using Spliced Transcripts Alignment to a Reference (STAR) (Dobin et al., 2012), with genome indexing as a first step. After the initial alignment, the splice junctions from each sample were merged together and the STAR Rn4 genome was regenerated with the splice junctions. The trimmed samples were then realigned to the re-indexed Rn4 genome. While the rat genome shows sparse annotation compared to both mouse and human levels [mouse 41,128 genes and human 60,155 genes (Harrow et al., 2012)], Ensembl (Flice et al., 2012) was chosen due to its relatively abundant gene annotation (29,516 for rat). Transcription factor annotation was downloaded from rat AnimalTFDB (Zhang et al., 2011).

### Differential and co-expression analysis

Following alignment to the genome, multimapped reads were discarded and only unique reads were counted to the Ensembl rat gene annotation Rattus_norvegicus.RGSC3.4.69.gtf (29,516 genes) using HTSeq with the union gene model (Anders et al., 2015) to study known gene expression (Figure S1A). To study retrotransposons and repeats, the repeatMasker track was downloaded from UCSC for the Rn4 gene build. Counting to the repeats was conducted as above. The repeats were then aggregated into their respective repeat type and had their counts combined using a custom R script (Figure S1B). *De novo* transcriptome assembly was conducted in order to find novel long noncoding RNAs (Figure S1C). Each sample was assembled using Trinity (Grabherr et al., 2011) and the assembled transcriptome was mapped back to the genomic coordinates using GMAP (Wu and Watanabe, 2005). Cuffmerge (Trapnell et al., 2010) was used to combine each assembled GFF file from GMAP into an uniform transcript file with the Ensembl gene annotation from above as a reference. Multiexonic unknown intergenic transcripts and antisense transcripts with exonic overlap was kept and their sequence was generated using gffread from the Cufflink package (Trapnell et al., 2010). PLEK (Li et al., 2014) was used on the transcript sequences to predict long noncoding RNAs. PLEK filters out transcripts smaller than 200 nt, and predicts if the transcript is coding or not. Transcripts remaining after the filtering steps are called noncoding transcripts, and their expression was counted with HTSeq as above.

Differential expression analysis was performed in EdgeR (Robinson et al., 2010), and the rest of the analysis was performed using custom R scripts or by various packages from Bioconductor (Gentleman et al., 2004). Novel lncRNAs showing differentially expression were then manually filtered by inspecting the transcripts in UCSC. Transcripts showing signs of association to its neighboring gene such as overlapping the neighboring gene in non-rat Refseq annotations, or belonging to the 3′UTR of Ensembl transcripts were filtered out. The reported differentially expressed lncRNA were those remaining after the manual filtering step.

Co-expression analysis was conducted using the R library Hmisc. Briefly for neighboring analysis, differentially expressed lncRNAs and mRNAs were intersected to find neighboring lncRNA-mRNA pairs. The expression of these were then correlated across all samples using Pearson coefficient, and pairs correlated with a *P* < 0.05 was deemed significant. Similar analysis was used for trans-correlation, however, neighbors were excluded from the analysis. Furthermore, the top DE genes from each time point were used to correlate the expression to lncRNAs and repeat elements. Here we used a *P* < 0.05 and an absolute Pearson coefficient of >0.75.

## Results

### NMDA receptor-dependent LTP-induction in rat dentate gyrus *in vivo*: validation of arc expression by transcriptomic analysis

**Figure 1:**
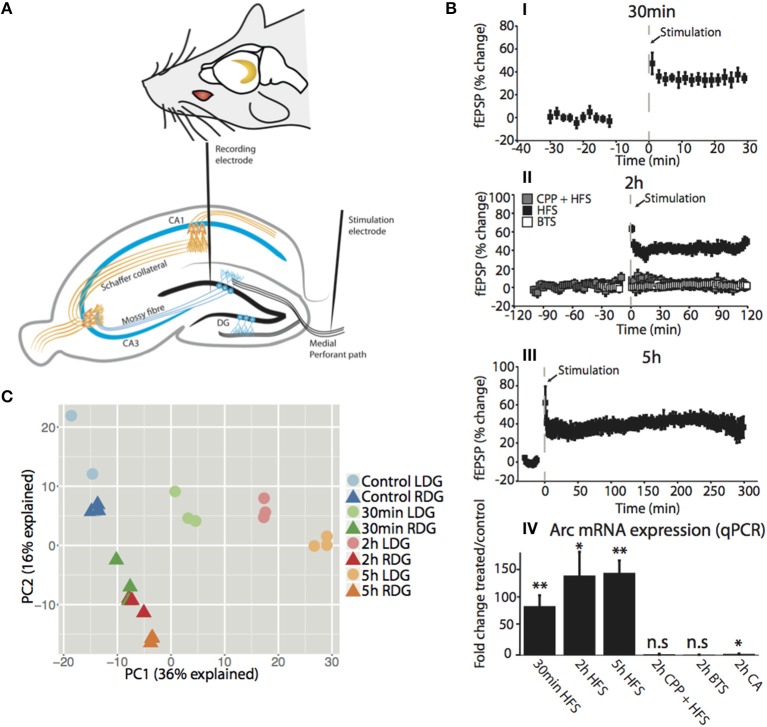
**Time dependent high frequency stimulation is the main driver of variation between dentate gyrus samples *in vivo***. **(A)** (top) Location of the hippocampus in both hemispheres of the brain. (bottom) Transverse section of the rat brain showing the hippocampal trisynaptic circuitry and the position of the stimulation electrode at the medial performant path and the registration electrode in the middle molecular layer of the dentate gyrus. **(B) (I–III)** Time course plot showing changes in the medial performant path-evoked fEPSP slope expressed as percentage of baseline. Experimental groups received HFS alone, HFS in the presence of NMDA receptor antagonist CPP, or only baseline test stimuli (BTS). CPP was injected IP at the dose of 10 mg/ml, 90 min prior to HFS. Dentate gyrus tissue was collected at the indicated time points post-HFS **(I)** 30min-HFS **(II)** 2h-HFS, 2h-CPP + HFS, 2h-BTS **(III)** 5h-HFS. Values are means (±s.e.m.) **(IV)** Quantitative PCR was used to validate the expression of the IEG Arc mRNA in the dentate gyrus (treated/control). PCR was performed in triplicate and normalized to the geometric mean of three housekeeping genes. Values are means (±s.e.m.), *n* = 5 in all groups except BLS and 30 min-HFS *n* = 4, ^*^ denotes *p* < 0.05, ^**^denotes *p* < 0.01; students paired *t*-test. **(C)** PCA plot showing the difference between the samples for the first two compartments with the amount of variation explained in the axis. Circles represent the stimulated dentate gyrus (LDG) while triangles represent the unstimulated contralateral side (RDG). The color of each time point is matched for ipsilateral (light) and contralateral (dark) side of the HFS hippocampus.

LTP was induced in the dentate gyrus (DG) of urethane-anesthetized rats using a well-characterized paradigm of patterned, high-frequency stimulation (HFS) of the medial perforant path input to DG granule cells (Figure 1A) (Messaoudi et al., 2002; Panja et al., 2009). Figure 1B shows the rapid, long-lasting increase in the slope of the field excitatory postsynaptic potential (fEPSP) at 30 min (35.0 ± 5.6%; mean ± s.e.m), 2 h (42.4 ± 8.1%; mean ± s.e.m) and 5 h (39.4 ± 6.8%; mean ± s.e.m) post-HFS (Figures 1BI–III). Treatment with the NMDA receptor antagonist, CPP, blocked induction of LTP (3.8 ± 4.5%; mean ± s.e.m), and no change was observed in the fEPSP in rats receiving baseline test stimulus (BTS) only (2.3 ± 2.8%; mean ± s.e.m) (Figure 1BIII). Next, we examined expression of activity-regulated cytoskeleton-associated gene (Arc) as a positive control for NMDA-receptor-dependent gene expression. NMDA receptor-dependent Arc mRNA expression is required for LTP consolidation (Guzowski et al., 2000; Messaoudi et al., 2007). qPCR analysis showed a robust (> 80-fold) increase in Arc mRNA expression at 30 min (*p* < 0.01), 2 h (*p* < 0.05), and 5 h (*p* < 0.01) post-HFS (Figure 1BIV). The increase in Arc expression was abolished in CPP-treated animals and was absent in the BTS control group, and only a slight increase (2-fold-change, *p* < 0.05) in Arc expression was observed in the neighboring cornu amonis (CA) region of the hippocampus compared to 30 min, 2 h, and 5 h (86 ± 19, 140 ± 37, 144 ± 19, mean fold-change ± s.e.m, respectively), post-HFS. The findings confirm HFS-induced, NMDA receptor-dependent LTP and Arc gene expression in the DG.

To characterize changes in both the mRNA and lncRNA transcriptome during the temporal development of LTP, we performed strand-specific, total RNA-seq using the Illumina HiSeq 2500 platform on RNA isolated from rat DG obtained at 30 min, 2 h, and 5 h post HFS. RNA was extracted separately from the left and right DG before conversion to cDNA. Naïve rats were used as controls. In all cases, the experimental side receiving HFS (LDG) was compared to the contralateral untreated side (RDG). Each time point was performed in three independent biological replicates. In total, 700 million 125 bp paired end reads were generated, averaging 30 million reads per sample (Table S2). The complete experimental workflow is outlined in Figure S1.

As a quality control of the RNA-Seq method, we examined Arc expression in the transcriptomic data. Robust increases in Arc mRNA expression were seen at 30 min (FDR = 5.6e^−132^), 2 h (FDR = 2.1e^−144^), and 5 h (FDR = 2.5e^−163^) post-HFS in the treated DG relative to the contralateral DG, which expressed Arc at the same low levels as naïve control (FDR = n.s.) (Figure S2B). The RNA-seq analysis reproduced the findings from qPCR performed in independent samples. To examine the robustness of the model, we undertook a principal components analysis (PCA) of the transcriptomic datasets. This analysis confirmed that differences between samples were driven by the stimulation itself and the time point after stimulation, indicated by PC1 explaining 36% of the variation (Figure 1C).

### RNA-seq analysis of known ensembl genes

**Figure 2:**
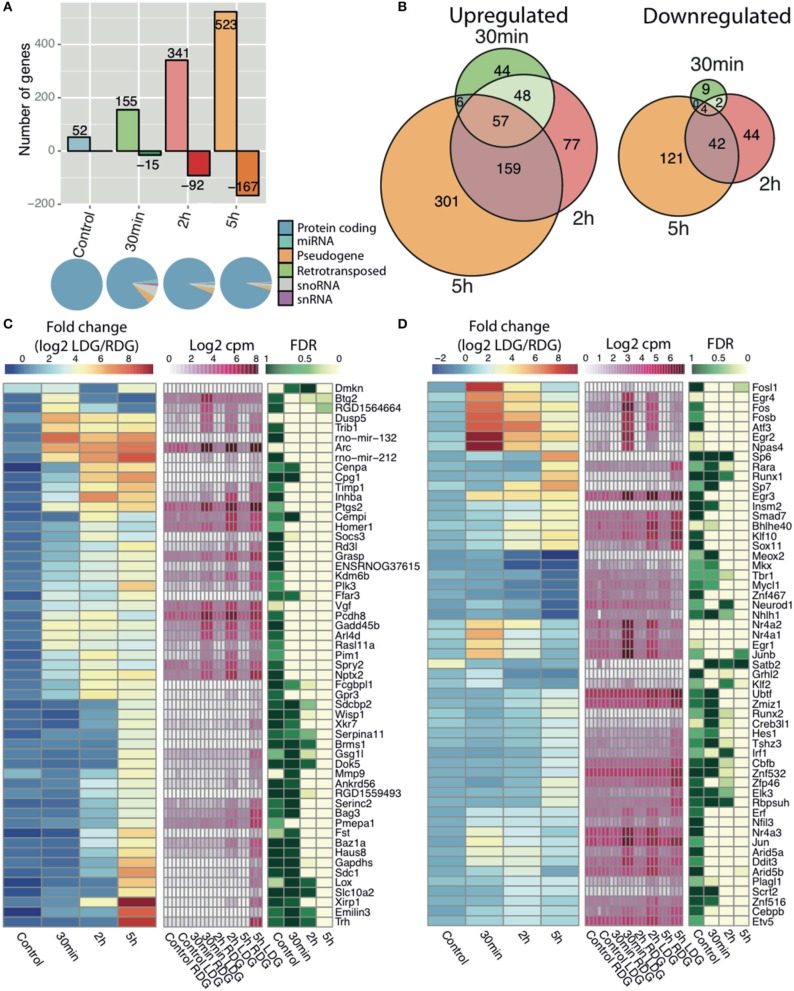
**RNA-seq reveals time dependent increase in transcription after high frequency stimulation**. **(A)** (Top) The number of differentially expressed genes comparing the experimental (LDG) and the contralateral side (RDG) of the dentate gyrus as either upregulated (positive) or downregulated (negative) with number of differentially expressed genes in each bar (|log2 FC|>1 and FDR < 0.05). (Bottom) Representation of Ensembl classes for each differentially expressed Ensemble gene. Classes were taken from the Ensembl gtf file. **(B)** Intersection of differentially expressed genes between the different time points, upregulated (left), and downregulated (right). Expression of Ensembl genes **(C)** and transcription factors **(D)** with greatest fold-change (|log2FC|>4 for genes and |log2FC|>2 for TFs) at any time point between the experimental (LDG) and control (RDG). Left key (blue/yellow/red) describes the fold-change value (log2 LDG/RDG), middle key (white/pink) denotes the expression values (log2 cpm), right key (green/white) represents the significance of the differential expression (FDR, *n* = 3).

To identify the key genes and study the pathways modulated during LTP, we performed differential expression (DE) analysis of Ensembl-annotated rat genes. The correlation between stimulated and unstimulated dentate gyrus was lowest 5 h post-stimulation (Figure S2A). We observed a dynamic temporal regulation of DE genes with an increase in the number of both upregulated and downregulated genes with time, with the vast majority of genes being protein-coding (Figures 2A,B). Although samples from naïve rat display 52 DE genes (Figure 2A), these were extremely low abundance transcripts (near the detection threshold) which showed less fold-change and higher FDR compared to the stimulated samples (Figure S2C); only 7 differentially expressed genes were shared between naïve and stimulated samples. The resulting DE genes can be found in Table S3. Out of 954 DE genes, the largest number of regulated genes (523 upregulated and 167 downregulated) was found 5 h post-HFS followed by 2 h (341 up-, and 92 downregulated), and 30 min (155 up-, and 15 downregulated) post-HFS, with the majority of genes being upregulated (Figure 2A). Consistent with this observation, the largest number of uniquely regulated genes (301 up, and 121 downregulated) occurred 5 h post-stimulation, with 22% of the genes shared between 5 and 2 h post-HFS, whereas 6% were upregulated and only 0.4% downregulated at all-time points (Figure 2B, left and right).

Gene set enrichment analysis indicated regulation of transcription and phosphatase/kinase pathways at all-time points, whereas signaling pathways, response to stimuli and synaptic transmission, and genes involved in developmental processes are enriched only at late time points (2 and 5 h) (Figure S3). The heatmaps show the temporal expression pattern of annotated Ensembl genes (Figure 2C) and transcription factors [Figure 2D, Annotated from AnimalTFDB (Zhang et al., 2011)] with the highest fold-change between LDG and RDG for each time point. Rapid activation of transcriptional programs is an essential part of synaptic plasticity. Numerous well-characterized early response genes encoding transcription factors such as C-fos, Erg1, and Npas4 (Flavell and Greenberg, 2008) are rapidly and transiently induced 30 min after LTP induction. In addition, we observed a second wave of transcription factor gene expression at 2 and 5 h after LTP induction. This late wave of IEGs included the zinc finger transcription factors SP6 and SP7, Runt-related transcription factor (Runx1), and retinoic acid receptor-alpha (RARα).

Gene ontology analysis of the earliest genes with increased expression, as identified by K-means clustering of the temporal profiles (Figure S4A), revealed that these genes were associated with presynaptic membrane components (Figure S4B) and involved in learning or memory (Figure S4C) and DNA-binding (Figure S4D). In agreement with previous studies (Ryan et al., 2011), we observed a rapid transient increase in a number of immediate early genes (IEGs), including transcription factors from the early growth response (Egr) and Fos families (Figure S2C, Figures 2C,D). In addition, the expression of a number of activity regulated genes previously associated with different forms of synaptic plasticity were increased including Arc, Homer1, Dual-specificity phosphatases (Dusps), ADP-ribosylation factor-like 4D (Arld4D), prostaglandin-endoperoxide synthase 2 (Ptsg2) (Figure 2C) (Wibrand et al., 2006; Ryan et al., 2011; Vallès et al., 2011). Moreover, our analysis reveals that primary microRNAs encoded by the mir212-132 cluster were upregulated, as previously reported (Vo et al., 2005; Nudelman et al., 2009; Wibrand et al., 2010).

As this was the first global transcriptomic analysis of synaptic plasticity, we examined the differential expression data to look for genes not previously associated with LTP. In total, we identified differential expression of 955 genes, out of the genes with a log2 fold-change over 4 (Figure 2C) and the transcription factors with a log2 fold-change over 2 (Figure 2D), we found 32 genes, and 27 transcription factors, that had no previously described role in synaptic plasticity from the literature. For example, Pmepa1 and Gsg1l are both expressed predominantly at the 5 h time point. Pmepa1 (prostate transmembrane protein androgen induced 1) encodes a protein (PMEPA1) that interacts with the ubiquitin-protein ligase NEDD4 (Xu et al., 2003). The present study associates over 60 highly differentially expressed genes previously not connected to LTP. We suggest further investigation of these genes is warranted to understand their role and mechanisms in synaptic plasticity.

### Identification of novel long noncoding RNAs involved in rat brain

Whereas an increasing number of lncRNAs have been annotated in both human and mice, the rat genome is still poorly annotated with respect to lncRNAs. To identify novel lncRNAs and study their involvement in LTP, we used Trinity to assemble a *de novo* transcriptome (Grabherr et al., 2011) (Figure S1C). Using our pipeline and filtering for transcripts with multiple exons and low coding potential (Figure S5A), we discovered 17,691 potential genes not previously described in the rat Ensembl annotation. These lncRNAs were divided into functional classes based on their position to known protein coding genes: Intergenic lncRNAs are more than 1 kb away from a known gene on both strands, antisense lnRNAs overlap with a gene on the opposite strand, bidirectional lncRNAs are less than 1 kb from a transcription start site (TSS) and potentially share the same promoter on the opposite strand, head-to-head lncRNA face the known gene's 3′UTR to the end of the lncRNA, 5′UTR-associated lnRNA are less than 1 kb upstream from the known gene, and 3′UTR-associated lncRNA are less than 1 kb away on the same strand from the 3′UTR end of the known gene (Figure S5B). Our novel lncRNAs share characteristics with the Gencode mouse and human lncRNAs in regard to exon number (Figure S5C) and exon length (Figure S5D). Following more stringent filtering to remove lowly expressed transcripts, we annotate a total of 10,256 novel lncRNAs in the rat transcriptome.

### Rapid regulation of long noncoding RNA expression in long-term potentiation *in vivo*

**Figure 3:**
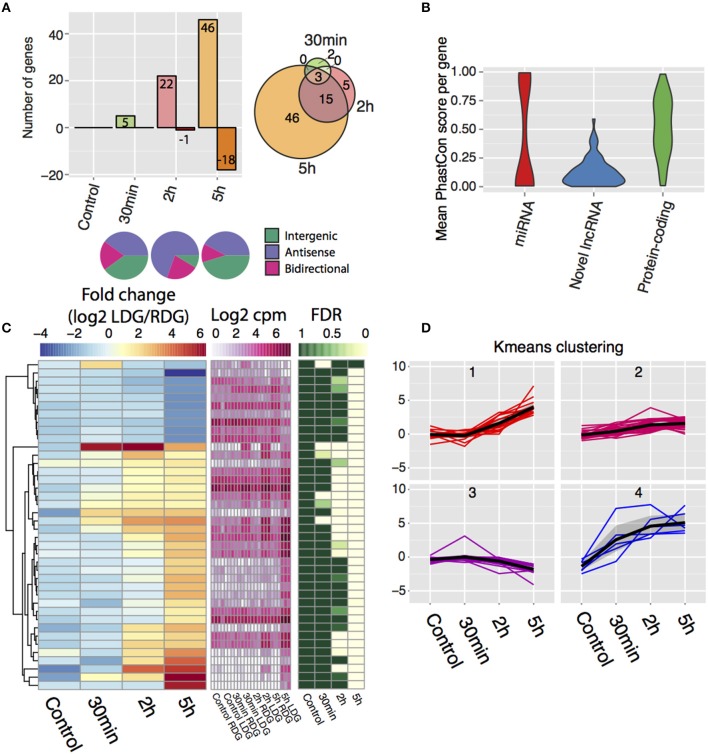
**De novo transcriptome assembly identifies novel differentially-expressed lncRNAs after high frequency stimulation. (A)** (Top left) Frequency of differentially expressed lncRNAs and direction of change post-stimulation with positive values indicating upregulated lncRNAs and negative indicating downregulated lncRNAs. (bottom) Class of lncRNAs that display differential expression for each time point. (top right) Intersection of differentially expressed lncRNA between each time point. **(B)** Conservation of the novel differentially expressed lncRNAs (blue) compared to Ensembl miRNA (red), protein-coding genes (green). (random 71 genes taken from each group). **(C)** Temporal expression of novel lncRNAs between the brain hemispheres. Left key (blue/yellow/red) describes the log2FC value, middle key (white/pink) denotes the expression values (log2 cpm), right key (green/white) represents the significance of the differential expression (FDR, *n* = 3). **(D)** K-means clustering of the temporal profile for the differentially expressed lncRNAs divided into four individual clusters.

Differential expression analysis, followed by manual filtering (see Materials and Methods), reveals 71 differentially expressed lncRNAs with the majority being upregulated (Figure 3A, Table S2) and with an increasing number of lncRNAs being differentially expressed after the 2 and 5 h time points (Figure 3A, left). After 30 min, only a few lncRNAs from each class change their expression, but after 2 h a bias toward differentially expressed antisense lncRNAs is observed with 16 antisense, 5 bidirectional, and 2 intergenic transcripts identified (Figure 3A, bottom). This bias disappears 5-h post-stimulation with an increased number of intergenic lncRNAs changing their expression (27 antisense, 8 bidirectional, and 29 intergenic). The majority of genes upregulated at 2 h remained elevated at 5 h (Figure 3A, right). Phastcon scores reveal that significantly regulated lncRNAs show less conservation than other genomic elements such as protein-coding genes across vertebrates (compared to mouse, human, dog, cow, opossum, chicken, frog, and zebrafish) on a base pair level, which is consistent with previous reports on lncRNA conservation (Johnsson et al., 2014) (Figure 3B). K-means clustering of the expression data revealed four distinct temporal patterns of expression (Figures 3C,D). In general, the direction, and temporal pattern of regulation of lncRNAs matches the patterns observed for mRNAs. Interestingly, one cluster shows a weak gradual decrease with time in expression (Figures 3C,D). However, in contrast to protein coding genes, downregulation of lncRNA expression was almost completely confined to the 5 h time point, with the exception of one lncRNA downregulated at 2 h. Thus, lncRNA downregulation appears to be delayed compared to protein coding genes. The genomic locations of all differentially expressed lncRNAs are presented in Table S4.

### LncRNAs are positively correlated to known LTP-genes

**Figure 4:**
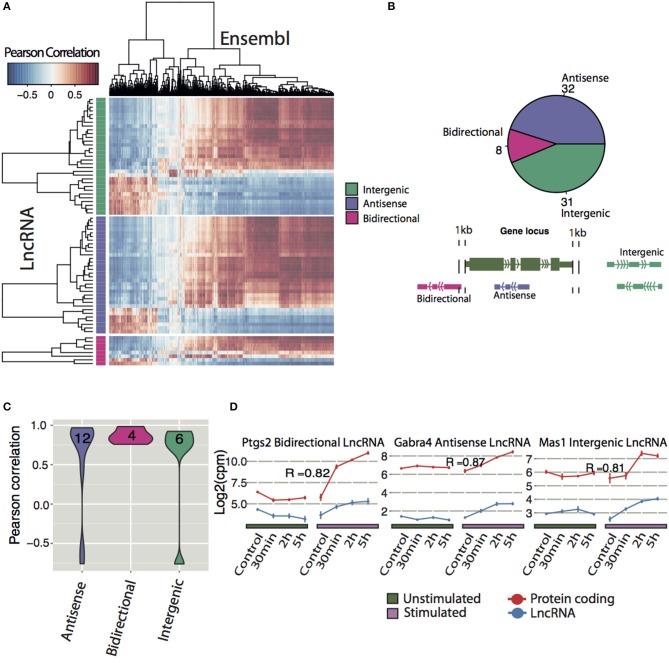
**Expression correlation analysis of lncRNAs with Ensembl genes**. **(A)** Correlation matrix between all differentially expressed lncRNAs (y-axis divided into their respective class) and differentially expressed Ensembl genes (x-axis). Pearson correlation was used to determine degree of correlation. **(B)** Classification of all differentially expressed novel lncRNA with a coding potential less than zero (Antisense: overlapping a protein-coding gene on the antisense, Bidirectional: < 1 kb from the TSS of the protein-coding gene on the opposite strand, Intergenic: >1 kb from the nearest protein-coding gene). **(C)** Frequencies of classes of differentially expressed lncRNAs that had highly correlated expression with a protein-coding neighbor (*p* < 0.05). **(D)** Examples of neighboring lncRNA-mRNA pairs (blue, red respectively) that had highly correlated expression. Pearson correlation coefficients (R) are indicated.

To infer possible functions of lncRNAs, we correlated differentially expressed lncRNAs with regulated protein coding genes. Of 71 differentially expressed lncRNAs, the majority of each class of lncRNAs (intergenic, antisense and bidirectional with number and classification shown in Figure 4B) were positively correlated with the majority of the protein coding genes, whereas a smaller proportion of lncRNAs were negatively correlated (Figure 4A).

Next the correlation between neighbor lncRNAs and Ensembl genes were explored. By only looking at differentially expressed neighboring lncRNA and protein coding pairs (23 in total), we found 22 correlated DE lncRNA that neighbor DE Ensembl genes with a *P* < 0.05 (Figure 4C). Divided into their classes, 6 intergenic and 12 antisense lncRNA show a range of positive and negative expression correlation to their neighboring gene. In contrast, 4 bidirectional lncRNAs, display high positive correlation with adjacent protein coding genes, suggesting that they share common regulatory mechanisms. Examples of differentially expressed lncRNA positively correlated to their neighbor Ensembl gene are shown in Figure 4D. Prostaglandin-endoperoxide synthase 2 (ptgs2), also known as cyclooxygenase-2 (COX-2), is an IEG (Yamagata et al., 1993) implicated in the induction phase of LTP and in memory and learning (Cowley et al., 2008). We identified an activity-induced lncRNA (XLOC_045589) in close proximity but on the opposite strand of ptgs2 (Figure 4D and Figure S6D). The expression of XLOC_045589 was confirmed with qPCR to be specific to NMDA-dependent LTP (Figure S6E). A conserved human ortholog was recently shown to positively regulate ptgs2 expression by sequestering repressive protein complexes from the Ptgs2 promoter and thereby facilitating the expression of Ptgs2 in human mammary epithelial cells (Krawczyk et al., 2014). However, it is yet to be determined whether the same mechanism applies in neuronal cells for this lncRNA-mRNA pair. Two other notable correlation pairs are the antisense lncRNA to gamma-aminobutyric acid (GABA) A receptor alpha 4 (Gabra4), a receptor subunit of the major inhibitory neurotransmitter in CNS and a nearby intergenic lncRNA to Mas-Related G Protein-Coupled Receptor A (Mas1), (Bartolomei, 2009; Shen et al., 2010).

**Figure 5:**
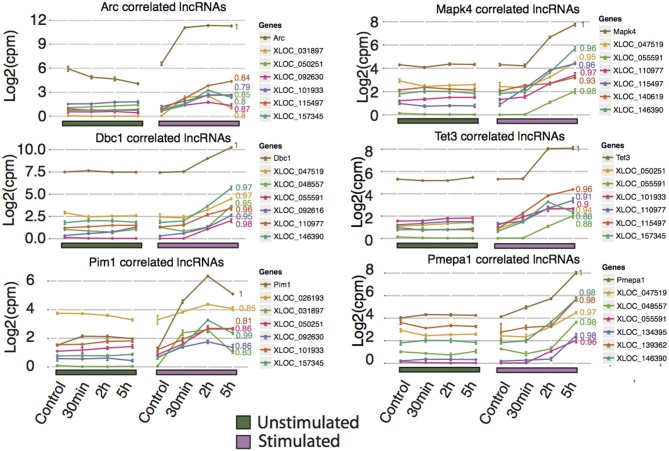
**Novel rat lncRNAs correlated to highly differentially expressed Ensembl genes and Arc mRNA**. The Ensembl genes chosen were those with the lowest FDR value for 2 and 5 h (no lncRNAs were identified at 30 min that showed high correlation to any Ensembl genes), and Arc. LncRNA with a neighboring Ensembl gene (from Figure 4C) were excluded from this analysis. The top 5 correlated lncRNAs with a *p* < 0.05 were plotted. Pearson correlation values are shown to the right of each line. Ensembl genes are colored brown and assigned a Pearson correlation value of 1, while the lncRNA are color-coded in each graph.

Unlike protein-coding genes, where function can be inferred by identification of known regulatory motifs, structural prediction, or orthology to other genes, there are no established rules for predicting the function of lncRNAs. Based on the premise that genes that show correlated expression profiles may be under a common regulatory architecture and therefore potentially share a common role, we investigated lncRNAs situated in trans relative to the differentially expressed protein-coding genes with focus on lncRNAs correlated to Arc and the most significantly differentially expressed protein-coding genes, Arc, Mapk4, Dbc1, Tet3, Pim1, and Pmepa1 (Figure 5). By applying this method, we found 34 lncRNAs highly correlated (*p* < 0.05 and Pearson correlation coefficient > 0.75). The corresponding lncRNA include XLOC_047519 corresponding to a lncRNA upstream of Tmem150c (Figure S6A), XLOC_139362 corresponding to the lincRNA Tunar (Figure S6B), and XLOC_055591 corresponding to a lncRNA overlapping, but not sharing any exons with, LOC306079, which does not show any sign of differential expression (Figure S6C). Tunar (TCL1 Upstream Neural Differentiation-Associated RNA), or in human TUNA (Figure S5B), was recently shown to be evolutionary conserved and required for neuronal differentiation (Lin et al., 2014). Deregulation of TUNA in the striatum has been suggested to be part of the pathophysiology of Huntington's disease. The present findings implicate Tunar in neuronal activity-dependent synaptic plasticity in the adult brain, however, these observations need to be tested further. In light of increasing numbers of lncRNAs with demonstrated roles in regulation, we propose that the lncRNAs described in this study may represent important targets for future biological studies in understanding the molecular mechanisms underlying LTP.

### Rapid, differential regulation of diverse repeat elements in long-term potentiation *in vivo*

**Figure 6:**
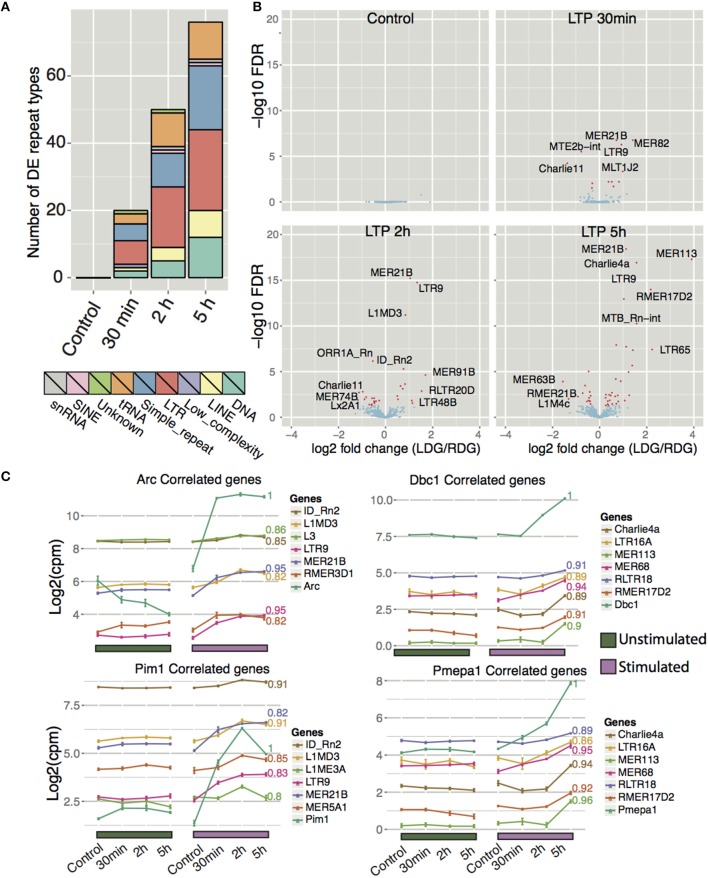
**Expression profiling of repeat elements reveals differentially expressed repeat types after high frequency stimulation. (A)** Frequency of significantly differentially expressed (FDR < 0.05) repeat types aggregated to their respective repeat class between LDG and RDG for naïve rats and for each time point. **(B)** Differential expression of different repeat types (excluding simple repeats, tRNA and low complexity repeat types) between each time point with significantly differentially expressed (FDR < 0.05) repeats colored red. **(C)** Expression correlation of Arc, Dbc1, Pim1, and Pmepa1 with different repeat types as in Figure 5, excluding simple repeats, tRNA, and low complexity repeat types.

Recent studies have shown that L1 repeat elements are expressed and that L1 and Alu elements are mobilized in the human brain. The resulting genomic mosaicism driven by retrotransposition is hypothesized to reshape the genetic circuitry of the brain and underpin normal and abnormal neurobiologial processes. In light of these observations, we hypothesized that retrotransposition may play a role in genomic reprogramming during memory formation in the rat brain. If this were the case, we would expect to see up-regulated expression of retrotransposable elements following HFS stimulation. Therefore, we examined uniquely mapping reads to all repeat-masked regions of the genome taken from the repeatMasker track downloaded from UCSC. Differential expression analysis between the left stimulated and the right unstimulated DG indeed showed that a wide array of repeat types, collapsed into their respective classes, displayed differential expression (Figure 6A and Table S5). The total number of differential expressed repeats (up- and down-regulated) increased with time (as seen for coding- and lncRNA genes).

The five major classes of differential expressed repeat element were class I transposons (retrotransposons) including long terminal repeat elements (LTRs) and long interspersed nuclear elements (LINEs), class II transposons (DNA), simple repeats, and tRNA (Figure 6A). As was the case for protein coding genes and lncRNA, most repeat types show increased expression after HFS-LTP. The most differentially expressed repeat types belong to either LTR or DNA repeats (Figure 6B). Moreover, many repeat types are highly correlated to the expression of LTP genes (Figure 6C, Figure S7D). MER21B, a LTR belonging to the ERV1 family, is upregulated early after stimulation and remains upregulated during all subsequent time points. The most upregulated individual repeat element was the MER113 DNA transposon, which was upregulated 16-fold 5 h after stimulation. Although simple repeats generally exhibited lower degrees of change compared to other repeat types, they are the second largest class of differentially expressed repeats, second only to LTRs (Figure 6A). Furthermore, the simple repeat (TCCCG)n displayed the highest degree of change across all time points with a 64-fold upregulation post-stimulation (Figure S7A).

A previous study showed that B2, a SINE element, is repressed in rat hippocampus after 30 min acute stress, and that H3K9me3 is responsible for this repression (Hunter et al., 2012). H3K9me3 enrichment was identified as selective for LTR/ERV, whereas H3K9me3 was depleted at DNA transposons and simple repeats, and most predominantly the tRNA family. In concordance with this, we observe highly upregulated expression of 11 tRNAs and of 24 different simple repeats after stimulation. However, we observe both up- and downregulation of LINEs and LTRs, while only one SINE type, ID_Rn2 (Figures 6A,B), was differentially expressed and, in our case, upregulated. The NMDA-receptor-dependent upregulation, and region specificity, of three specific ID_Rn2 was confirmed by qPCR, using unique primers (Figure S7C).

To infer the potential function of the DNA transposon, LINE, LTR, and SINE elements in LTP, we correlated their profiles with the major expression profile clusters generated for protein-coding genes (Figure 6C, Figure S7D). We identified many repeat types that positively correlated with DE coding genes associated with synaptic plasticity (Figure S7B). In summary, our analysis of repeat elements during LTP shows many distinct classes of elements being differentially expressed. Previous studies have shown changes in repeat element expression during aging and in alcoholism and PTSD. We propose that these elements may play a function in synaptic plasticity, however, these findings need to be studied further to investigate the potential function of repeat elements in brain.

## Discussion

This study presents the first global transcriptomic analysis following *in vivo* rat LTP induction revealing the rapid regulation of lncRNA, repeat elements, and tRNA. This is the first evidence linking these major, functionally diverse classes of RNA to synaptic plasticity in adult brain. Importantly, the present study confirmed the differential expression of numerous previously reported IEGs and shows the NMDA-dependent expression of Arc, validating the approach chosen. As well as providing the most detailed annotation of the rat brain transcriptome, the analysis also greatly extends the set of differentially expressed protein-coding genes (900 Ensembl-annotated genes), and provides evidence of a late expression wave of transcription factors associated with LTP. This is exemplified by Runx1 which is considered a master regulator of haematopoiesis and is also implicated in the proliferation and differentiation of neural progenitor cells in the olfactory epithelium, although no role in post-mitotic neurons in brain has been described (Theriault, 2005). RARα signaling is implicated in homeostatic control of synaptic strength through regulation of local translation in dendrites (Chen et al., 2014). The present results show late transcriptional upregulation of RARα, but further studies are needed to address contributions of RARα protein expression to LTP. Altogether we identified over 60 highly differentially expressed genes not previously associated with LTP, such as Pmepa1 and Gsg1l. PMEP1 is thought to function as a membrane-bound protein interacting with downstream signaling molecules through WW- and SH3-binding domains (Giannini et al., 2003). Germ cell-specific gene 1-like protein (Gsg1l), an auxiliary subunit of the AMPA-receptor, can boost AMPA receptor surface expression and modify its electrophysiological properties (Haering et al., 2014). The expression of Gsg1l is upregulated at 5 h, but not at earlier time points (30 min and 2 h) after LTP induction. Moreover, abnormal Gsg1l expression levels have been linked to Huntington's disease (Becanovic et al., 2010).

Several studies couple lncRNA expression to brain development (Amaral et al., 2009; Lipovich et al., 2012; Pauli et al., 2012), aging (Wood et al., 2012), and pluripotency or neuronal fate specification (Dinger et al., 2008a; Mercer et al., 2010; Ng et al., 2011; Aprea et al., 2013). In situ hybridization analysis has shown brain region-specific expression patterns and subcellular localization of lncRNAs (cis-antisense, linc, and bidirectional) (Mercer et al., 2008). Specific lncRNAs have also been implicated in synaptogenesis (Bernard et al., 2010), local dendritic protein synthesis (Lin et al., 2008), and short-term hippocampus-dependent memory (Anguera et al., 2011). However, no systematic analysis has been done to investigate the importance of lncRNAs in long-term synaptic plasticity.

The present study demonstrates regulation of lncRNA expression in relation to synaptic activation and LTP formation in the medial perforant path input to the dentate gyrus *in vivo*. Following LTP induction and stringent filtering, 71 lncRNAs were identified as differentially expressed. Already 30 min after LTP, 5 lncRNAs were upregulated and the number of DE lncRNAs increased with time after stimulation. After initial (30 min) expression of a few lncRNAs from all classes, antisense transcripts predominate at 2 h post-HFS, while late (5 h) expression was more restricted to intergenic transcript. Our analysis revealed 22 lncRNAs to be highly correlated to their neighboring DE protein-coding gene, indicating potential lncRNA-mRNA regulatory pairs while 34 lncRNAs were highly correlated to known, highly significant LTP DE genes in trans, indicating their possible importance in LTP.

Repeat elements can be seen as marks of earlier transposition events. Although repeat elements make up one third of the genome in mammals (de Koning et al., 2011), they have been long considered as evolutionary debris, lacking function. Although originally thought to be confined to germ cells, pluripotent cells, and cancer cells, retrotransposition has since been demonstrated to occur in somatic cells, and specifically in neuronal precursors derived from rat hippocampal stem cells (Muotri et al., 2005). Retrotransposition in neuronal progenitors is now well documented in both rodents and humans (Coufal et al., 2009; Baillie et al., 2011; Richardson et al., 2014). Here we show, for the first time, that different classes of repeat elements are rapidly, and temporally, transcribed in the context of long-term synaptic plasticity.

The mechanism that drives the expression of repeat elements following stimulation was not explored here. However, it has been previously demonstrated that LTP and learning influences epigenetic marks and therefore we speculate that the activation of retrotransposon transcription might be a consequence of the activity-dependent modulation of chromatin remodeling factors (Lipsky, 2013; Telese et al., 2013). Acute behavioral stress (environmental) can induce epigenetic effects that impact expression of specific retrotransponsons including B2 SINEs (Grabherr et al., 2011; Hunter et al., 2012). Indeed, the repression of numerous noncoding and repetitive elements has been shown to be mediated by a histone methyltransferase (SETDB1) regulating H3K9me3 (Karimi et al., 2011).

Expressed retrotransposons are thought to insert back into the genome at different locations to either increase or disrupt transcription of neighboring genes, creating a somatic mosaicism that influences neuronal type specificity and diversity. Moreover, depletion of RNAi regulatory proteins in the fly brain results in enhanced retrotransposition in specific neuronal types in the mushroom body, a brain region involved in memory formation (Perrat et al., 2013). This raises the question of whether a conserved mechanism for increased retrotransposition exists in the hippocampus and other memory-relevant circuits in mammals (Erwin et al., 2014). It is intriguing to consider that expression of repeat elements during LTP is the first step toward retrotransposition and reshaping of the neuronal genome. A hypothetical mechanism for how these repeat elements could be linked to memory, would be that a certain stimuli, whether it is stress or a learning task (here LTP), deregulate the repression of repeat elements which are then rapidly and transiently transcribed. These elements reinsert themselves back into the genome of stimulated neurons where they influence the expression of neighboring genes. It has now been shown that each individual hippocampal neuron contains an average of 13.7 novel somatic L1 insertions enriched in hippocampal genes and neuronal stem cell enhancers (Upton et al., 2015). The present work supports the intriguing hypothesis that dynamic retrotransposition may act as a molecular means to reprogram the neuronal genome as part of long-term synaptic plasticity and memory formation (Mattick and Mehler, 2008).

In summary, the results presented here reveal a vast extension of mRNAs previously not associated with neuronal plasticity; the discovery of extensive, dynamic regulation of lncRNAs, repeat elements, and tRNA following LTP induction in the adult rat brain. Activity-dependent gene expression is fundamental to the formation of neural circuits and the remodeling of neuronal connectivity with experience. Many neurodevelopmental and psychiatric disorders have been linked to dysregulation of activity-dependent gene expression and synaptic function. Our data provides new insights into the molecular underpinnings of synaptic plasticity and builds a case for further study of this novel regulatory repertoire in neurological disease.

### Data access

Raw FASTQ files from the RNA-seq experiments have been deposited with ArrayExpress under the accession number E-MTAB-2072.

## Author contributions

JM led the project and performed the bioinformatic analysis. SP conducted the electrophysiology experiments. SP contributed to electrophysiology. KW extracted the RNA and conducted qPCRs. IS contributed to RNA extraction. DK prepared the samples for RNA-sequencing. KW, JM, and MD designed the study. J, KW, CB, and MD wrote the manuscript.

## Funding

This work was supported by the Research Council of Norway (grants 204861 and 199355), Bergen Medical Research Foundation, and the University of Bergen (KW).

### Conflict of interest statement

The authors declare that the research was conducted in the absence of any commercial or financial relationships that could be construed as a potential conflict of interest.
