# Corrigendum: Dynamic expression of long noncoding RNAs and repeat elements in synaptic plasticity

**DOI:** 10.3389/fnins.2016.00354

**Published:** 2016-07-27

**Authors:** Jesper L. V. Maag, Debabrata Panja, Ida Sporild, Sudarshan S. Patil, Dominik C. Kaczorowski, Clive R. Bramham, Marcel E. Dinger, Karin Wibrand

**Affiliations:** ^1^Genomics and Epigenetics Division, Garvan Institute of Medical ResearchSydney, NSW, Australia; ^2^Faculty of Medicine, St Vincent's Clinical School, University of New South WalesSydney, NSW, Australia; ^3^Department of Biomedicine and K.G. Jebsen Centre for Research on Neuropsychiatric Disorders, University of BergenBergen, Norway

**Keywords:** long noncoding RNA (lncRNA), LTP (long term potentiation), retrotransposons, repeat elements, rat brain, time-series data, synaptic plasticity (LTP/LTD)

Reason for Corrigendum:

There was a mistake in the accession number as published. The accession number in the published version is referred to as E-MTAB-2072 while the correct accession number is E-MTAB-3375. The authors apologize for the mistake. This error does not change the scientific conclusions of the article in any way.

## Author contributions

JM led the project and performed the bioinformatic analysis. DP conducted the electrophysiology experiments. SP contributed to electrophysiology. KW extracted the RNA and conducted qPCRs. IS contributed to RNA extraction. DK prepared the samples for RNA-sequencing. KW, JM, and MD designed the study. JM, KW, CB, and MD wrote the manuscript.

## Funding

This work was supported by the Research Council of Norway (grants 204861 and 199355), Bergen Medical Research Foundation, and the University of Bergen (KW).

### Conflict of interest statement

The authors declare that the research was conducted in the absence of any commercial or financial relationships that could be construed as a potential conflict of interest.

