# Supplementary material for: Dynamic expression of long noncoding RNAs and repeat elements in synaptic plasticity
Source: Front Neurosci. 2015 Oct 1;9:351. doi: 10.3389/fnins.2015.00351 (PMC4589673; doi:10.3389/fnins.2015.00351)
Supplement: Supplementary file 1 [file DataSheet1.DOCX]

**Supplementary Material**

**Dynamic expression of long noncoding RNAs and repeat elements in synaptic plasticity**

Jesper L.V. Maag, Debabrata Panja, Ida Sporild, Sudarshan Patil, Dominik C. Kaczorowski, Clive R. Bramham, Marcel E. Dinger, Karin Wibrand

**Supplemental figures**

Figure S1. Pipeline of analysis

Figure S2. Differential expression of Ensembl genes

Figure S3. Gene set enrichment

Figure S4. K-means clustering and GO analysis

Figure S5. De novo assembly and characterisation of novel lncRNAs

Figure S6. Novel LTP-associated lncRNAs

Figure S7. Repeat analysis and correlation of simple repeats

**Supplemental tables**

Table S1. Primers

Table S2. Number of reads per sample

Table S3. Differentially expressed Ensembl genes

Table S4. Differentially expressed novel lncRNAs

Table S5. Differentially expressed repeat elements


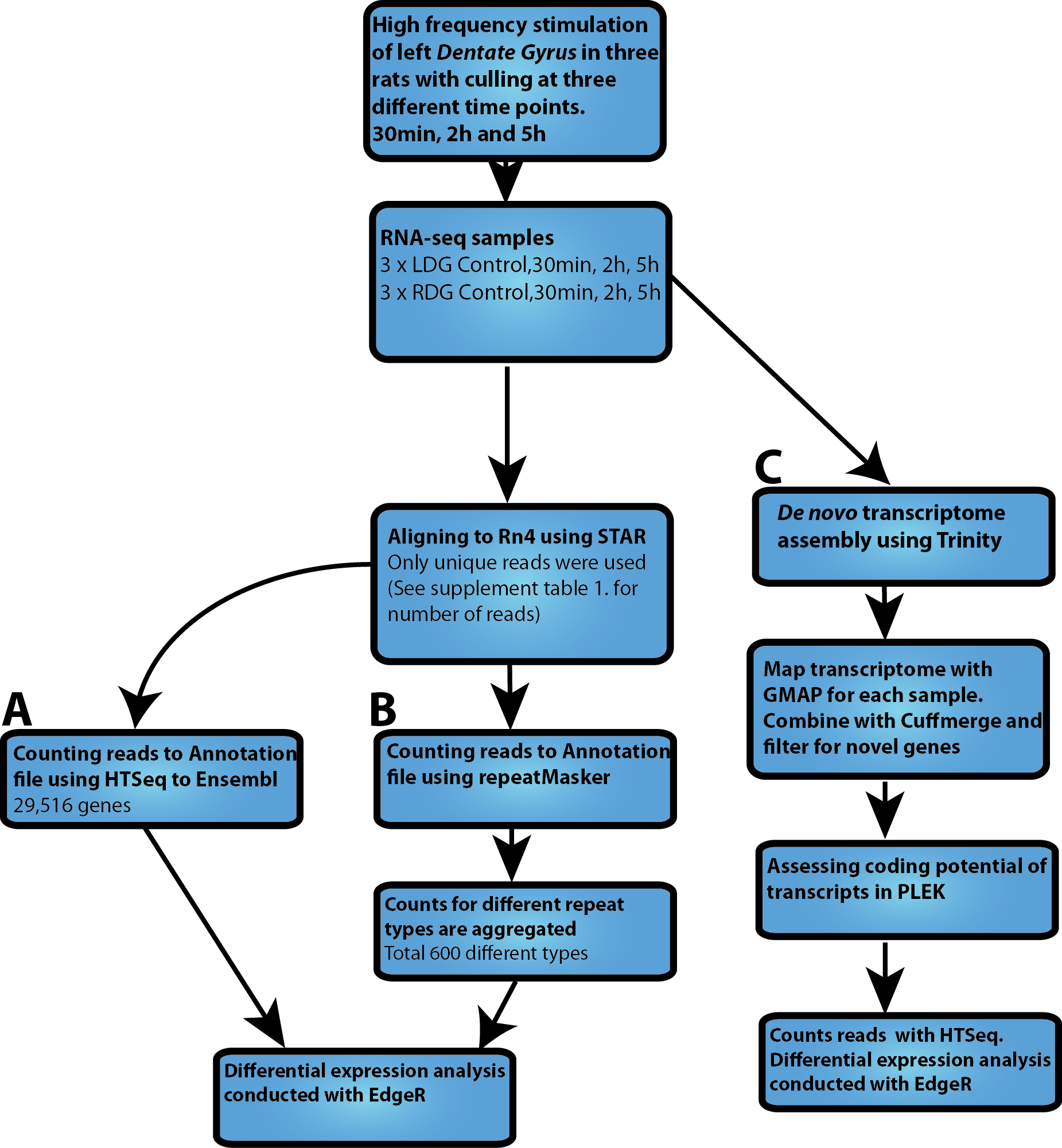


***Supplementary figure 1****. Analytical pipeline. High frequency stimulation in the left dentate gyrus was performed on urea-anesthetised rats at different time points. Naïve rats were used as controls. RNA was extracted from both left and right dentate gyrus and pooled and sequenced on 7 lanes on a HiSeq 2500. Reads were trimmed with trimgalore on default, and aligned to the Rn4 genome with STAR. Only uniquely mapped reads were aligned. Reads were counted to (A) Ensembl transcriptome and (B) repeatmasker for repeat analysis. (C) De novo assembly was done with Trinity for each sample. De novo transcripts were then aligned back to the genome with GMAP and merged with Cufflinks. Coding potential was measured with PLEK. Differential expression was measured with edgeR.*


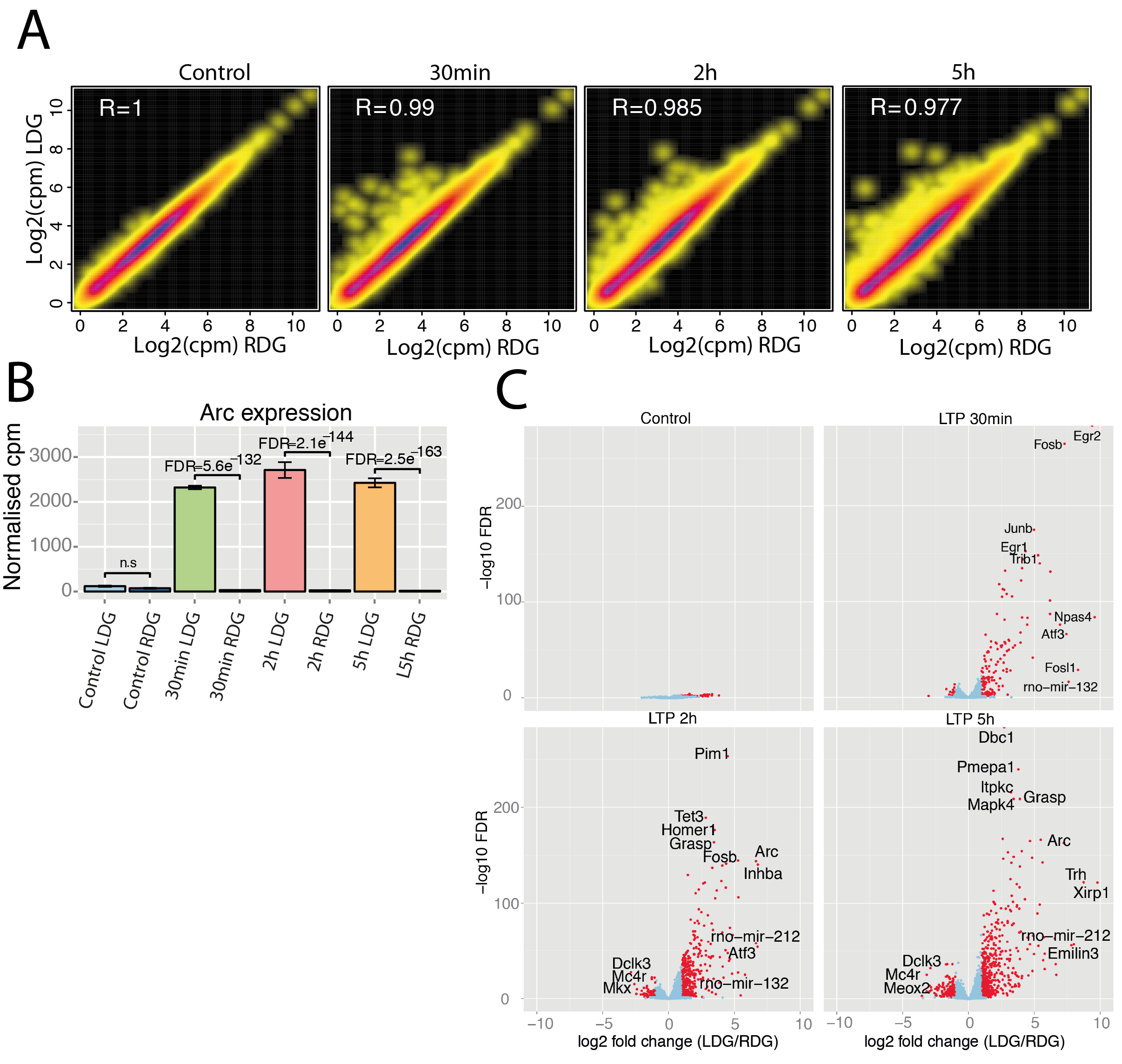


***Supplementary figure 2****. Differential expression of Ensembl genes. (A) Ensembl gene expression of all genes with over 1 count per million in at least 3 samples which totalled 14,025 genes. Log2 expression in left DG (y-axis) and right DG (x-axis) was plotted against each other for the naïve rats and for each time point in a density scatterplot. The colours represent the density of the number of genes with blue, red, orange, yellow representing the highest to lowest density respectively. R was measured with spearman correlation (the mean expression value for each time point is shown, n=3. (B) Arc mRNA expression is shown as normalized counts per million reads. Reads were counted with HTSeq and normalization was done using the TMM method in EdgeR (mean ±s.e.m, FDR values from EdgeR). (C) Volcano plot comparing gene expression (log2 fold changes and FDR) of left stimulated DG over right unstimulated DG for Control, 30min, 2h, and 5h. Differentially expressed genes (log2FC >1 and FDR <0.05) between the brain halves*


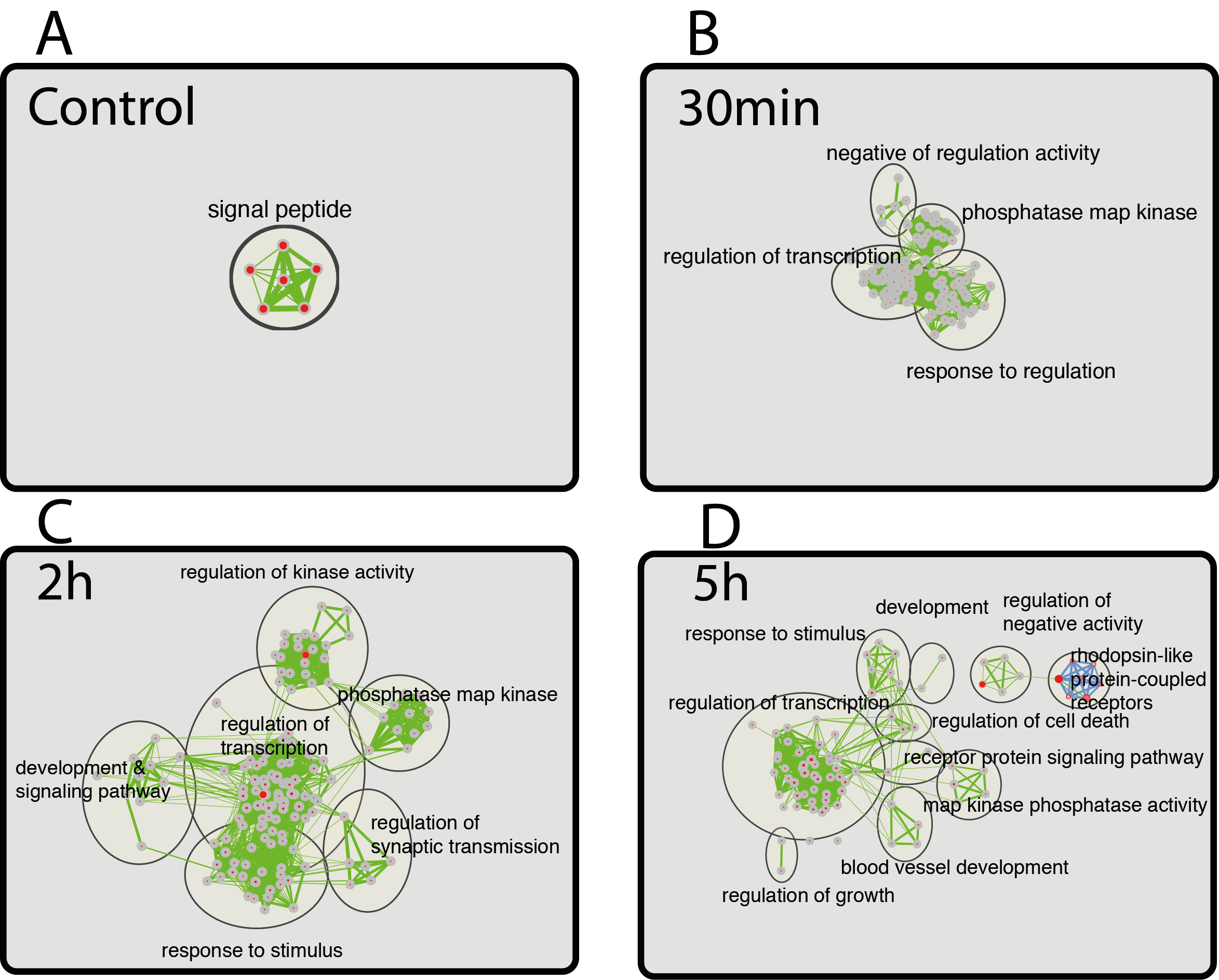


***Supplementary figure 3.*** *Gene set enrichment. Gene set enrichment map showing the enrichment for the up (green) and down (blue) regulated genes between (A) Control, (B) 30min, (C) 2h, and (D) 5h. Differentially expressed genes HFS and control side group were entered into DAVID to obtain gene set enrichments. Results were plotted with Cytoscape 3.20 BETA using the EnrichmentMap, clustermaker, and WordCloud apps.*


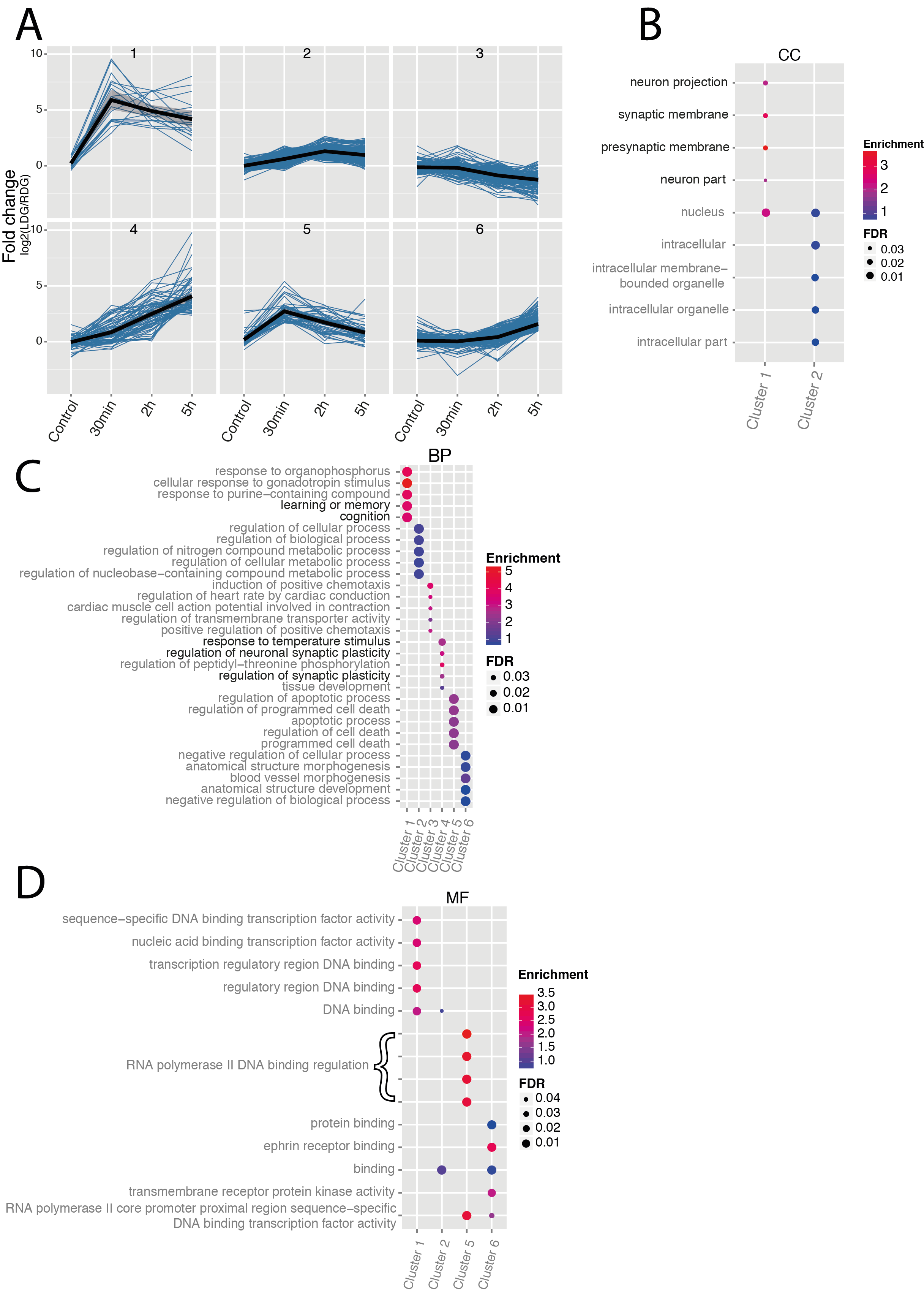


***Supplementary figure 4.*** *Kmeans clustering and Gene ontology enrichment analysis showing the full temporal dynamic of the transcriptome. (A) Log2FC values of LDG/RDG at each time point were averaged from 3 rats, and divided into 6 kmeans clusters depending on their temporal changes (black line equals mean). Top 5 terms of gene ontology enrichment (FDR <0.05) for (B) cellular compartment, (C) biological function, (D) molecular function for each kmeans cluster were plotted against each other to compare the cluster enrichments for the specific temporal expression profile. Size of the dots represents the FDR-value while the colour represents the log2 Enrichment values*

*
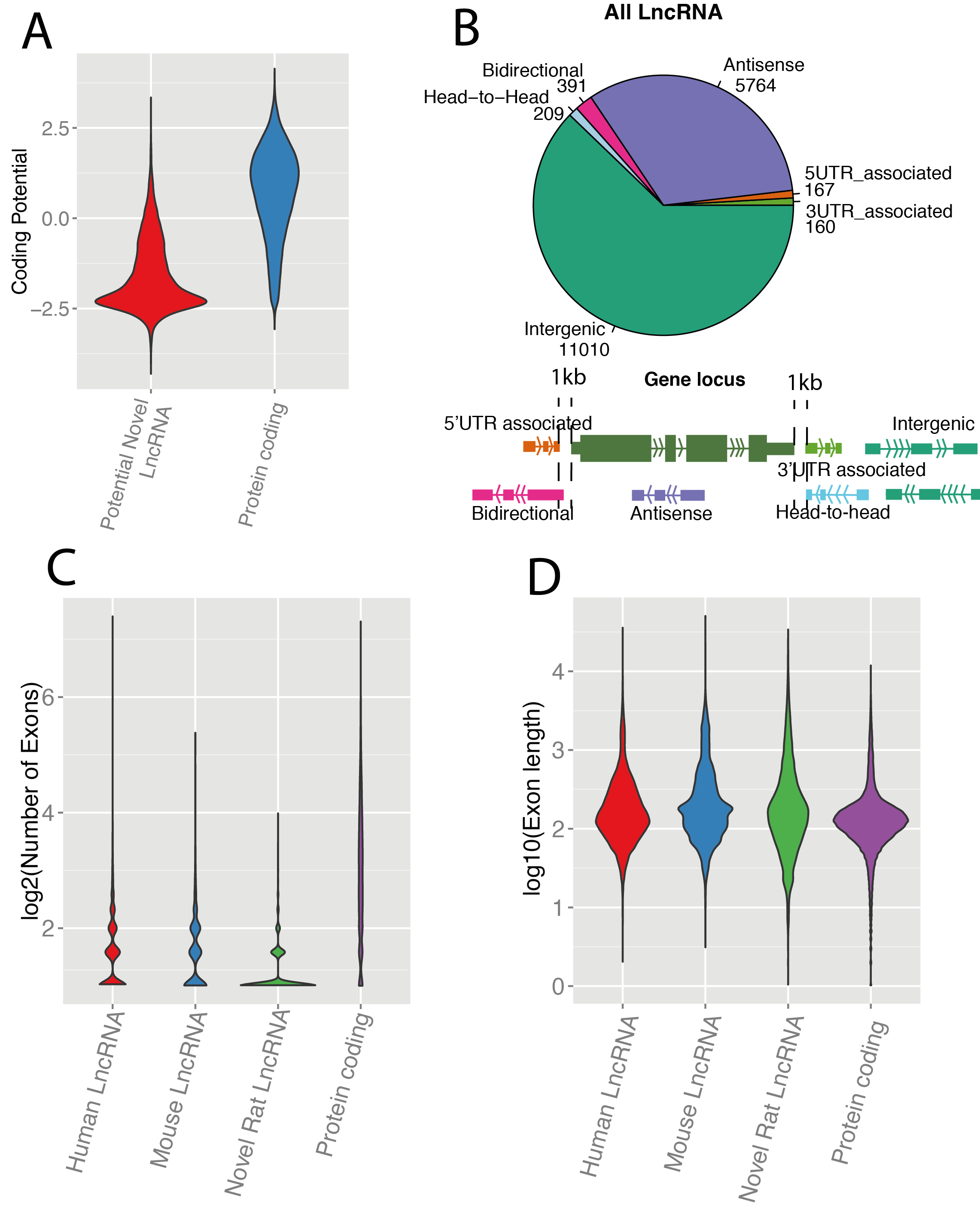
*

***Supplementary figure 5.*** *De novo assembly and characterisation of novel lncRNAs. (A) Coding potential of all novel transcripts found through the de novo pipeline (left, red n=*17,691*) compared to the coding potential of all the protein-coding transcripts in the Ensembl Rn4 gene annotation file (left, blue). (B) Classification of all lncRNA with a coding potential less than zero (Antisense: overlapping a protein-coding gene on the antisense, Bidirectional: <1kb from the TSS of the protein-coding gene on the opposite strand, Intergenic: >1kb from the nearest protein-coding gene, 5’UTR associated: <1kb form the TSS on the same strand as the protein-coding gene, 3’UTR associated: <1kb from the nearest protein-coding gene on the same strand. Comparison of number of exons (C) and the exon length (D) between rat ensemble genes (purple), human (red), mouse (green) and novel rat (green) lncRNA.*

*
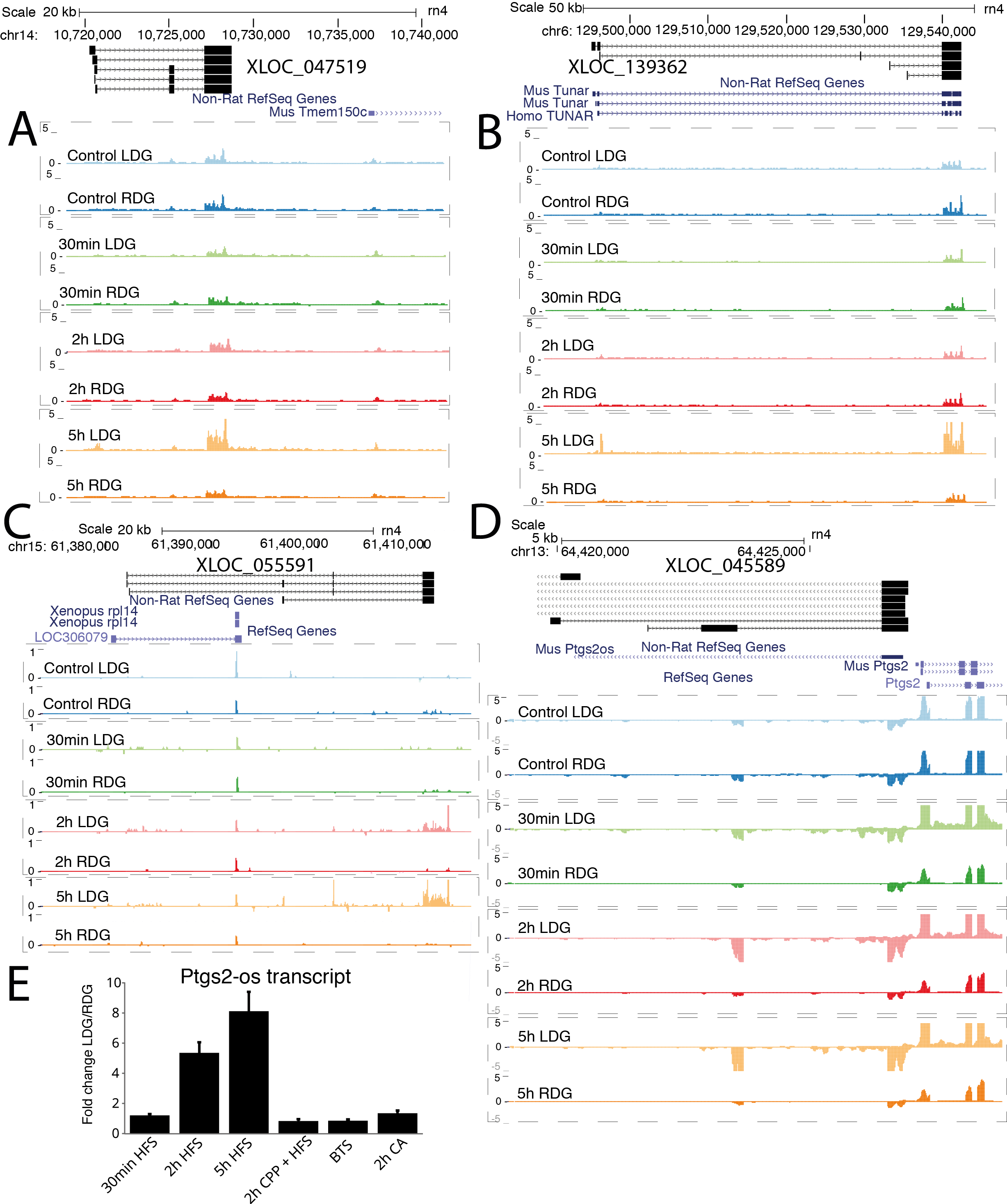
*

***Supplementary figure 6****. Novel LTP-associated lncRNA. Examples of novel lncRNAs found in this study by either neighbouring gene, or guilt-by-association analysis. (A) Tmem150c upstream lncRNA, (B) Tunar, (C) Loc306079 overlapping lncRNA, and (D) Ptgs2 bidirectional lncRNA. (E) qPCR showing the fold change of Ptgs2-os transcripts between stimulated and unstimulated DG per time point, NMDA-receptor inhibitor (CPP), and in the CA.*

*
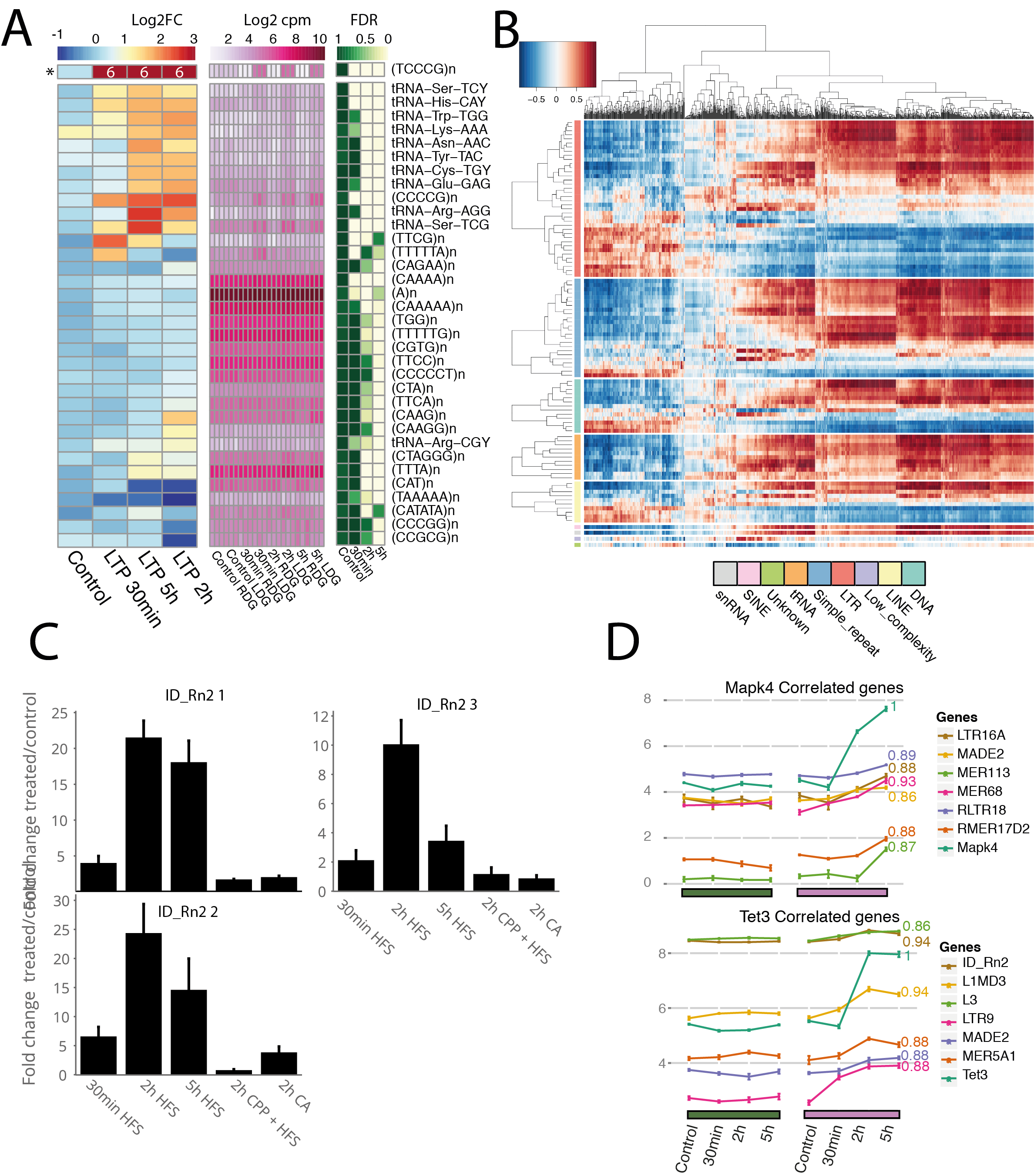
*

***Supplementary figure 7.*** *Repeat analysis and correlation of simple repeats, tRNA. (A) Heatmap showing the temporal profile of the simple repeats and tRNA with highest log2 fold change. (TCCCG)n had a log2 fold change of 6 in each stimulated sample, but is set here to 3 to retain the resolution of the other repeats. Left (blue/yellow/red) describes the Log2FC value, middle (white/pink) denotes the expression values (log2 cpm), right (green/white) represents the significance of the differential expression (FDR, n=3). (B) Correlation plot with all the differentially expressed repeat types clustered by class and compared to the differentially expressed Ensembl genes. Measured with Pearson correlation. (C) qPCR of three unique ID_Rn2 repeats showing fold change for each time point, and displaying NMDA-receptor dependency and region specificity (D) Guilt-by-association analysis for repeats correlated with Mapk4 and Tet3.*

**Table S1.** Primers

| Gene | Primer pair |  |
| --- | --- | --- |
| Cyclopholine | FW: AGCACTGGGGAGAAAGGATT | |
|  | BW: GATGCCAGGACCTGTATGCT | |
| Hypoxanthine-guanine phosphoribosyltransferase | FW: TCCACTTTCGCTGATGACAC | |
|  | BW: GCAGACTTTGCTTTCCTTGG | |
| Polyubiqutine | FW: GGCAAGACCATCACCCTAGA | |
|  | BW: GCAGGGTTGACTCTTTCTGG | |
| Arc | FW: CCCAGTCTGTGGCTTTTGTCA | |
|  | BW: GTGTCAGCCCCAGCTCAATC | |
| Ptgs bidirectional RNA (XLOC_045589) | FW: ACGTCGGAATGCTAAGTGTC | |
|  | BW: GTTAGATCCCAGCGTTCAAGTA | |
| ID_Rn2 1 | FW: GGGATTTAGCTCAGTGGTAGAG | |
|  | BW: TCGCTCCAGTCTTAAACCTAAC | |
| ID_Rn2 2 | FW: CCCTCCTGACAATCAAACTAAGA | |
|  | BW: AATTCCTCCCAGTTCTCTGTTAC | |
| ID_Rn2 3 | FW: GGTTAGAAGGGAGTAGCAGAAA | |
|  | BW: CAGTCAAACAGCTTTGTCAGG | |

**Table S2.** Reads and mapped reads per sample

| Sample ID | Tissue/treatment | OD260/280 | RIN | Total Reads | Uniquely mapped reads | Uniquely mapped reads (%) |
| --- | --- | --- | --- | --- | --- | --- |
| D1 | LDG1 2h LTP | 2,07 | 8,8 | 29,056,732 | 23,031,906 | 79.3 |
| D2 | RDG1 2h LTP | 2,07 | 8,7 | 23,413,365 | 18,664,165 | 79.7 |
| D5 | LDG2 2h LTP | 2,09 | 8,9 | 24,308,012 | 19,341,330 | 79.6 |
| D6 | RDG2 2h LTP | 2,08 | 8,9 | 31,379,980 | 25,733,461 | 82.0 |
| D9 | LDG3 2h LTP | 2,11 | 8,8 | 25,674,677 | 19,850,890 | 77.3 |
| D10 | RDG3 2h LTP | 2,1 | 8,9 | 26,923,168 | 19,816,166 | 73.6 |
| D31 | LDG1 5h LTP | 2,1 | 8,8 | 40,662,513 | 31,705,683 | 78.0 |
| D32 | RDG1 5h LTP | 2,09 | 8,9 | 48,584,497 | 39,613,828 | 81.5 |
| D35 | LDG2 5h LTP | 2,11 | 8,9 | 21,836,010 | 16,892,071 | 77.4 |
| D36 | RDG2 5h LTP | 2,1 | 8,9 | 31,626,354 | 24,884,589 | 78.7 |
| D39 | LDG3 5h LTP | 2,1 | 8,8 | 29,893,594 | 23,338,039 | 78.1 |
| D40 | RDG3 5h LTP | 2,1 | 8,9 | 38,949,720 | 30,233,190 | 77.6 |
| D51 | LDG1 30 min LTP | 2,1 | 8,8 | 23,680,889 | 19,038,079 | 80.4 |
| D52 | RDG1 30 min LTP | 2,09 | 9 | 21,848,116 | 17,307,245 | 79.2 |
| D56 | LDG2 30 min LTP | 2,07 | 8,9 | 24,078,828 | 19,121,834 | 79.4 |
| D57 | RDG2 30 min LTP | 2,1 | 8,9 | 27,860,673 | 21,571,274 | 77.4 |
| D61 | LDG3 30 min LTP | 2,1 | 8,8 | 23,324,443 | 18,089,770 | 77.6 |
| D62 | RDG3 30 min LTP | 2,08 | 8,7 | 27,139,027 | 21,140,365 | 77.9 |
| D71 | LDG1 naive | 2,1 | 8,7 | 37,487,264 | 29,376,291 | 78.4 |
| D72 | RDG1 naive | 2,1 | 8,7 | 25,655,155 | 20,733,226 | 80.8 |
| D78 | LDG2 naive | 2 | 8,3 | 27,227,303 | 21,048,430 | 77.3 |
| D79 | RDG2 naive | 2,04 | 8,6 | 30,362,037 | 23,338,630 | 76.9 |
| D85 | LDG3 naive | 2,09 | 8,4 | 31,133,107 | 24,215,513 | 77.8 |
| D86 | RDG3 naive | 2,09 | 8,7 | 41,474,979 | 32,436,901 | 78.2 |

**Table S3.** Differentially expressed Ensembl genes

| **Condition** | **EnsembleID** | **Genes** | **logFC** | **logCPM** | **PValue** | **FDR** |
| --- | --- | --- | --- | --- | --- | --- |
| Control | ENSRNOG00000037371 | D3ZPQ3_RAT | 1.54 | 0.60 | 7.64E-09 | 0.000107126 |
| Control | ENSRNOG00000008697 | Nov | 3.27 | 4.49 | 2.03E-08 | 0.000142026 |
| Control | ENSRNOG00000005185 | Nxph3 | 3.03 | 1.93 | 1.01E-07 | 0.000378927 |
| Control | ENSRNOG00000010834 | Mpped1 | 2.68 | 5.20 | 1.08E-07 | 0.000378927 |
| Control | ENSRNOG00000032660 | Lphn2 | 2.73 | 3.47 | 1.38E-07 | 0.000387355 |
| Control | ENSRNOG00000003073 | F1M5K1_RAT | 2.90 | 2.59 | 1.78E-07 | 0.000416826 |
| Control | ENSRNOG00000025209 | D4AA77_RAT | 1.43 | 3.36 | 8.32E-07 | 0.001458059 |
| Control | ENSRNOG00000002053 | F1M3H3_RAT | 1.73 | 4.31 | 1.78E-06 | 0.002773356 |
| Control | ENSRNOG00000005108 | Wfs1 | 2.58 | 4.95 | 2.31E-06 | 0.002802104 |
| Control | ENSRNOG00000009577 | Ndst4 | 2.43 | 3.03 | 2.37E-06 | 0.002802104 |
| Control | ENSRNOG00000027309 | D4AA49_RAT | 1.67 | 1.96 | 2.40E-06 | 0.002802104 |
| Control | ENSRNOG00000007079 | Met | 3.25 | 1.96 | 3.11E-06 | 0.003359163 |
| Control | ENSRNOG00000010412 | F1LXL0_RAT | 3.79 | 0.55 | 4.87E-06 | 0.00388503 |
| Control | ENSRNOG00000013042 | Htr1b | 2.63 | 1.40 | 5.15E-06 | 0.00388503 |
| Control | ENSRNOG00000025032 | D4A0N6_RAT | 1.24 | -0.12 | 5.00E-06 | 0.00388503 |
| Control | ENSRNOG00000033143 | Ddc8 | 1.51 | -0.59 | 4.21E-06 | 0.00388503 |
| Control | ENSRNOG00000005797 | Sla | 1.42 | 0.56 | 6.36E-06 | 0.004209502 |
| Control | ENSRNOG00000013515 | Ptpru | 2.42 | 3.73 | 7.66E-06 | 0.004672936 |
| Control | ENSRNOG00000017704 | Sema3f | 1.29 | 0.28 | 1.15E-05 | 0.006423638 |
| Control | ENSRNOG00000019297 | Homer2 | 1.11 | 4.00 | 1.45E-05 | 0.007237151 |
| Control | ENSRNOG00000032827 | F1M0R9_RAT | 1.08 | 0.65 | 1.42E-05 | 0.007237151 |
| Control | ENSRNOG00000000288 | Scarf2 | 1.28 | 0.84 | 2.10E-05 | 0.009499876 |
| Control | ENSRNOG00000022358 | D4AEC9_RAT | 1.65 | 0.19 | 2.92E-05 | 0.012409765 |
| Control | ENSRNOG00000004554 | Dcn | 2.16 | 2.91 | 4.80E-05 | 0.01727284 |
| Control | ENSRNOG00000021266 | Prokr2 | 2.77 | 0.22 | 4.58E-05 | 0.01727284 |
| Control | ENSRNOG00000032327 | Pdia5 | 1.19 | -0.12 | 4.46E-05 | 0.01727284 |
| Control | ENSRNOG00000017659 | Hs3st2 | 1.97 | 1.41 | 5.07E-05 | 0.01777592 |
| Control | ENSRNOG00000002238 | Sema5b | 1.01 | 2.69 | 8.17E-05 | 0.02208228 |
| Control | ENSRNOG00000005723 | NMD3A_RAT | 1.48 | 2.74 | 7.74E-05 | 0.02208228 |
| Control | ENSRNOG00000007189 | Ttc22 | 1.36 | 1.34 | 8.00E-05 | 0.02208228 |
| Control | ENSRNOG00000007843 | Wnt2 | 1.36 | 3.32 | 8.18E-05 | 0.02208228 |
| Control | ENSRNOG00000014028 | F1LXJ6_RAT | 1.19 | 0.23 | 8.19E-05 | 0.02208228 |
| Control | ENSRNOG00000026128 | Cpne8 | 1.49 | 2.70 | 7.55E-05 | 0.02208228 |
| Control | ENSRNOG00000027645 | Tmem90a | 1.48 | -0.15 | 7.55E-05 | 0.02208228 |
| Control | ENSRNOG00000010694 | Tpbg | 1.90 | 1.63 | 8.86E-05 | 0.023435993 |
| Control | ENSRNOG00000002773 | Rgs4 | 1.00 | 5.61 | 0.000104482 | 0.026639221 |
| Control | ENSRNOG00000002196 | LOC100361733 | 1.26 | 3.32 | 0.000126033 | 0.029386131 |
| Control | ENSRNOG00000010188 | Satb2 | 2.95 | 0.32 | 0.000127226 | 0.029386131 |
| Control | ENSRNOG00000023337 | Sema3a | 1.61 | 0.35 | 0.00012783 | 0.029386131 |
| Control | ENSRNOG00000024055 | Dmkn | 2.56 | -1.24 | 0.000122244 | 0.029386131 |
| Control | ENSRNOG00000003288 | CCG5_RAT | 1.76 | -0.12 | 0.000151411 | 0.032827249 |
| Control | ENSRNOG00000017539 | Mmp9 | 1.53 | 1.89 | 0.00018064 | 0.036187435 |
| Control | ENSRNOG00000015860 | Gipr | 2.14 | 0.13 | 0.00018709 | 0.036438442 |
| Control | ENSRNOG00000002987 | Myh2 | 1.10 | 1.62 | 0.000195063 | 0.036542314 |
| Control | ENSRNOG00000010972 | Neurog2 | 1.36 | -0.46 | 0.000198047 | 0.036542314 |
| Control | ENSRNOG00000025120 | Gli1 | 1.16 | -0.06 | 0.000222692 | 0.039035161 |
| Control | ENSRNOG00000000036 | Klhdc8a | 1.28 | 0.26 | 0.000228675 | 0.039589089 |
| Control | ENSRNOG00000002097 | Rasl11b | 1.25 | 3.20 | 0.000244412 | 0.040245949 |
| Control | ENSRNOG00000002579 | Parm1 | 1.14 | 2.55 | 0.000242397 | 0.040245949 |
| Control | ENSRNOG00000011202 | Chrna4 | 1.08 | 2.79 | 0.00024682 | 0.040245949 |
| Control | ENSRNOG00000014010 | Gfra2 | 1.35 | 1.00 | 0.000252608 | 0.040716423 |
| Control | ENSRNOG00000013610 | Chrna5 | 1.28 | 2.06 | 0.000269534 | 0.042950858 |
| 30 min | ENSRNOG00000000640 | Egr2 | 9.40 | 5.24 | 0 | 0 |
| 30 min | ENSRNOG00000016811 | Fosb | 7.29 | 5.06 | 4.11E-269 | 2.88E-265 |
| 30 min | ENSRNOG00000042838 | Junb | 4.98 | 7.29 | 3.76E-179 | 1.76E-175 |
| 30 min | ENSRNOG00000019422 | Egr1 | 4.34 | 6.61 | 1.25E-156 | 4.37E-153 |
| 30 min | ENSRNOG00000004100 | Trib1 | 5.29 | 4.60 | 3.13E-152 | 8.77E-149 |
| 30 min | ENSRNOG00000019822 | Gadd45b | 4.15 | 6.55 | 3.74E-148 | 8.74E-145 |
| 30 min | ENSRNOG00000007607 | Nr4a1 | 5.40 | 5.91 | 8.75E-144 | 1.75E-140 |
| 30 min | ENSRNOG00000020770 | Arl4d | 4.07 | 5.67 | 1.10E-138 | 1.94E-135 |
| 30 min | ENSRNOG00000020938 | Ppp1r15a | 2.79 | 5.64 | 6.22E-136 | 9.70E-133 |
| 30 min | ENSRNOG00000043465 | Arc | 6.22 | 9.91 | 3.97E-135 | 5.57E-132 |
| 30 min | ENSRNOG00000017828 | Egr3 | 4.00 | 8.00 | 9.06E-126 | 1.16E-122 |
| 30 min | ENSRNOG00000006436 | Spty2d1 | 2.34 | 4.46 | 5.52E-122 | 6.46E-119 |
| 30 min | ENSRNOG00000005285 | Fbxo33 | 2.59 | 6.90 | 1.11E-116 | 1.20E-113 |
| 30 min | ENSRNOG00000003687 | Rgs2 | 2.70 | 6.20 | 6.06E-116 | 6.07E-113 |
| 30 min | ENSRNOG00000033433 | Csrnp1 | 2.90 | 4.94 | 1.93E-111 | 1.81E-108 |
| 30 min | ENSRNOG00000011238 | D3ZMH5_RAT | 3.31 | 6.60 | 4.86E-109 | 4.26E-106 |
| 30 min | ENSRNOG00000023509 | Irs2 | 2.54 | 7.01 | 1.89E-108 | 1.56E-105 |
| 30 min | ENSRNOG00000014061 | Dusp5 | 6.19 | 4.30 | 9.42E-105 | 7.34E-102 |
| 30 min | ENSRNOG00000015719 | Egr4 | 6.20 | 4.69 | 1.72E-90 | 1.27E-87 |
| 30 min | ENSRNOG00000013703 | Siah2 | 2.18 | 4.63 | 1.97E-90 | 1.38E-87 |
| 30 min | ENSRNOG00000020009 | Npas4 | 9.57 | 4.36 | 4.06E-87 | 2.71E-84 |
| 30 min | ENSRNOG00000003300 | Btg2 | 4.08 | 6.68 | 9.30E-87 | 5.93E-84 |
| 30 min | ENSRNOG00000021687 | RGD1564664 | 4.49 | 4.63 | 1.35E-86 | 8.22E-84 |
| 30 min | ENSRNOG00000000529 | Pim1 | 2.74 | 4.26 | 1.20E-84 | 7.02E-82 |
| 30 min | ENSRNOG00000008015 | Fos | 6.95 | 6.22 | 2.21E-79 | 1.24E-76 |
| 30 min | ENSRNOG00000005600 | Nr4a2 | 4.46 | 6.72 | 3.64E-79 | 1.96E-76 |
| 30 min | ENSRNOG00000000956 | Rasl11a | 3.95 | 3.03 | 4.69E-71 | 2.43E-68 |
| 30 min | ENSRNOG00000003745 | Atf3 | 7.44 | 5.03 | 2.33E-69 | 1.17E-66 |
| 30 min | ENSRNOG00000018232 | Srf | 1.30 | 6.40 | 1.01E-68 | 4.87E-66 |
| 30 min | ENSRNOG00000011921 | Dusp4 | 3.05 | 5.32 | 1.72E-63 | 8.03E-61 |
| 30 min | ENSRNOG00000018714 | Arl5b | 3.10 | 5.89 | 2.23E-62 | 1.01E-59 |
| 30 min | ENSRNOG00000013520 | RGD1560523 | 1.71 | 5.79 | 1.08E-61 | 4.75E-59 |
| 30 min | ENSRNOG00000005964 | Nr4a3 | 2.73 | 6.84 | 3.73E-61 | 1.58E-58 |
| 30 min | ENSRNOG00000001416 | Vgf | 2.75 | 6.77 | 8.87E-61 | 3.66E-58 |
| 30 min | ENSRNOG00000002828 | Tob1 | 2.08 | 5.13 | 1.50E-60 | 6.03E-58 |
| 30 min | ENSRNOG00000002525 | Ptgs2 | 3.97 | 9.04 | 2.58E-60 | 1.01E-57 |
| 30 min | ENSRNOG00000002837 | Ier2 | 2.80 | 3.20 | 2.29E-59 | 8.67E-57 |
| 30 min | ENSRNOG00000042188 | ENSRNOG00000042188 | 2.15 | 5.75 | 2.65E-59 | 9.79E-57 |
| 30 min | ENSRNOG00000013101 | PCDH8_RAT | 3.53 | 8.53 | 2.45E-57 | 8.82E-55 |
| 30 min | ENSRNOG00000001006 | Nptx2 | 2.54 | 7.11 | 2.74E-57 | 9.61E-55 |
| 30 min | ENSRNOG00000010058 | Spry2 | 2.47 | 5.96 | 3.16E-57 | 1.08E-54 |
| 30 min | ENSRNOG00000006789 | Ddit3 | 2.49 | 5.97 | 9.95E-56 | 3.32E-53 |
| 30 min | ENSRNOG00000006329 | Peli1 | 1.69 | 5.42 | 1.39E-55 | 4.52E-53 |
| 30 min | ENSRNOG00000000781 | Rnf39 | 3.39 | 5.61 | 5.73E-55 | 1.83E-52 |
| 30 min | ENSRNOG00000024363 | Sertad1 | 2.22 | 4.53 | 2.76E-53 | 8.61E-51 |
| 30 min | ENSRNOG00000026293 | Jun | 3.08 | 7.49 | 8.23E-53 | 2.51E-50 |
| 30 min | ENSRNOG00000011951 | Plk2 | 2.92 | 8.58 | 2.41E-51 | 7.18E-49 |
| 30 min | ENSRNOG00000026880 | D3Z8K5_RAT | 1.49 | 5.99 | 5.29E-51 | 1.55E-48 |
| 30 min | ENSRNOG00000016326 | Cx3cl1 | 1.52 | 8.97 | 1.52E-50 | 4.35E-48 |
| 30 min | ENSRNOG00000006532 | H3f3b | 1.44 | 8.35 | 3.29E-50 | 9.24E-48 |
| 30 min | ENSRNOG00000025764 | Mt1a | 2.72 | 5.02 | 3.48E-47 | 9.57E-45 |
| 30 min | ENSRNOG00000015382 | Arid5a | 2.74 | 4.66 | 4.71E-47 | 1.27E-44 |
| 30 min | ENSRNOG00000023446 | LOC689986 | 1.22 | 7.59 | 1.80E-46 | 4.77E-44 |
| 30 min | ENSRNOG00000035476 | rno-mir-212 | 4.88 | 1.34 | 1.33E-44 | 3.45E-42 |
| 30 min | ENSRNOG00000007387 | Per1 | 2.42 | 6.48 | 4.22E-43 | 1.08E-40 |
| 30 min | ENSRNOG00000012623 | Arf4 | 1.24 | 7.39 | 5.59E-43 | 1.40E-40 |
| 30 min | ENSRNOG00000024114 | D4A582_RAT | 1.71 | 7.02 | 4.18E-42 | 1.03E-39 |
| 30 min | ENSRNOG00000016122 | Hmgcr | 1.30 | 7.53 | 1.75E-40 | 4.23E-38 |
| 30 min | ENSRNOG00000004284 | Btg1 | 2.24 | 6.53 | 4.29E-40 | 1.02E-37 |
| 30 min | ENSRNOG00000012193 | Cited2 | 2.68 | 5.73 | 2.47E-39 | 5.78E-37 |
| 30 min | ENSRNOG00000010653 | RGD1306119 | 2.10 | 5.41 | 2.37E-37 | 5.45E-35 |
| 30 min | ENSRNOG00000025142 | D3ZFL6_RAT | 2.12 | 4.75 | 3.97E-37 | 8.98E-35 |
| 30 min | ENSRNOG00000007346 | Grasp | 1.57 | 6.62 | 1.03E-35 | 2.29E-33 |
| 30 min | ENSRNOG00000000130 | Dnajb5 | 1.34 | 7.97 | 1.64E-35 | 3.59E-33 |
| 30 min | ENSRNOG00000007029 | Dnaja1 | 1.07 | 8.66 | 1.08E-33 | 2.32E-31 |
| 30 min | ENSRNOG00000043168 | Homer1 | 1.40 | 6.70 | 6.99E-33 | 1.48E-30 |
| 30 min | ENSRNOG00000014320 | Inhba | 2.92 | 5.21 | 1.33E-32 | 2.79E-30 |
| 30 min | ENSRNOG00000020552 | Fosl1 | 8.33 | -0.83 | 9.26E-32 | 1.91E-29 |
| 30 min | ENSRNOG00000018484 | Plk3 | 2.64 | 3.77 | 4.05E-31 | 8.23E-29 |
| 30 min | ENSRNOG00000038047 | LOC100362769 | 3.20 | 2.00 | 5.25E-31 | 1.05E-28 |
| 30 min | ENSRNOG00000018107 | Zfand5 | 1.01 | 8.02 | 2.39E-30 | 4.72E-28 |
| 30 min | ENSRNOG00000037613 | Kdm6b | 2.07 | 5.36 | 3.29E-30 | 6.41E-28 |
| 30 min | ENSRNOG00000007126 | Gpr19 | 1.58 | 4.08 | 4.10E-30 | 7.88E-28 |
| 30 min | ENSRNOG00000013090 | Gadd45g | 2.92 | 5.56 | 1.79E-29 | 3.40E-27 |
| 30 min | ENSRNOG00000017801 | Atf4 | 1.09 | 7.60 | 5.12E-29 | 9.57E-27 |
| 30 min | ENSRNOG00000016690 | Idi1 | 1.92 | 5.80 | 1.46E-28 | 2.69E-26 |
| 30 min | ENSRNOG00000006859 | Insig1 | 1.52 | 7.21 | 1.67E-24 | 3.00E-22 |
| 30 min | ENSRNOG00000038044 | Tsc22d2 | 1.14 | 5.48 | 3.00E-24 | 5.32E-22 |
| 30 min | ENSRNOG00000001189 | Sik1 | 1.82 | 5.54 | 5.71E-24 | 1.00E-21 |
| 30 min | ENSRNOG00000039274 | ENSRNOG00000039274 | 1.50 | 6.16 | 5.87E-24 | 1.02E-21 |
| 30 min | ENSRNOG00000011586 | Ccnl1 | 1.77 | 5.62 | 7.67E-24 | 1.31E-21 |
| 30 min | ENSRNOG00000001277 | Mafk | 1.37 | 4.45 | 1.74E-23 | 2.95E-21 |
| 30 min | ENSRNOG00000020426 | Erf | 1.56 | 4.92 | 2.21E-23 | 3.70E-21 |
| 30 min | ENSRNOG00000004720 | Kcnj2 | 2.40 | 2.76 | 1.17E-22 | 1.93E-20 |
| 30 min | ENSRNOG00000021264 | Pcna | 1.22 | 5.36 | 2.08E-22 | 3.39E-20 |
| 30 min | ENSRNOG00000000461 | Brd2 | 1.02 | 7.90 | 2.78E-22 | 4.48E-20 |
| 30 min | ENSRNOG00000004624 | Rnd3 | 1.52 | 4.81 | 1.58E-21 | 2.49E-19 |
| 30 min | ENSRNOG00000014456 | Coq10b | 1.50 | 5.08 | 2.33E-21 | 3.63E-19 |
| 30 min | ENSRNOG00000023896 | Dusp6 | 1.96 | 4.79 | 1.22E-20 | 1.88E-18 |
| 30 min | ENSRNOG00000028513 | ENSRNOG00000028513 | 1.79 | 2.41 | 1.58E-20 | 2.41E-18 |
| 30 min | ENSRNOG00000003329 | Ppm1d | 1.11 | 5.76 | 1.25E-19 | 1.82E-17 |
| 30 min | ENSRNOG00000010448 | Ptbp1 | 1.20 | 4.68 | 3.07E-19 | 4.35E-17 |
| 30 min | ENSRNOG00000011668 | Nfil3 | 1.57 | 3.56 | 4.96E-19 | 6.89E-17 |
| 30 min | ENSRNOG00000035516 | rno-mir-132 | 7.61 | -0.07 | 7.62E-19 | 1.05E-16 |
| 30 min | ENSRNOG00000011026 | Pqcp | 1.00 | 5.29 | 1.08E-18 | 1.47E-16 |
| 30 min | ENSRNOG00000010888 | Ankrd33b | 1.10 | 5.54 | 1.72E-18 | 2.32E-16 |
| 30 min | ENSRNOG00000039902 | LOC683626 | 1.36 | 4.90 | 1.75E-18 | 2.34E-16 |
| 30 min | ENSRNOG00000027368 | Fam100a | 1.10 | 4.80 | 3.33E-18 | 4.36E-16 |
| 30 min | ENSRNOG00000003251 | B3galt2 | 1.21 | 6.42 | 1.56E-17 | 2.01E-15 |
| 30 min | ENSRNOG00000018275 | Errfi1 | 1.13 | 6.69 | 2.70E-17 | 3.42E-15 |
| 30 min | ENSRNOG00000000748 | Dstnl1 | 1.17 | 3.78 | 3.09E-17 | 3.86E-15 |
| 30 min | ENSRNOG00000037615 | ENSRNOG00000037615 | 2.13 | 2.85 | 3.67E-17 | 4.55E-15 |
| 30 min | ENSRNOG00000006368 | Lrrn3 | 1.13 | 7.06 | 1.51E-16 | 1.77E-14 |
| 30 min | ENSRNOG00000006118 | Klf10 | 1.20 | 6.56 | 1.74E-16 | 2.02E-14 |
| 30 min | ENSRNOG00000003809 | Sat1 | 1.29 | 6.82 | 2.28E-16 | 2.55E-14 |
| 30 min | ENSRNOG00000001099 | Rbak | -1.05 | 3.56 | 2.80E-16 | 3.12E-14 |
| 30 min | ENSRNOG00000028834 | Polr2a | 1.10 | 6.10 | 3.65E-15 | 3.90E-13 |
| 30 min | ENSRNOG00000010208 | Timp1 | 2.60 | 3.61 | 1.45E-14 | 1.50E-12 |
| 30 min | ENSRNOG00000014886 | Fam107b | 1.17 | 5.21 | 2.28E-14 | 2.33E-12 |
| 30 min | ENSRNOG00000016571 | Ngf | 2.30 | 3.62 | 3.67E-14 | 3.73E-12 |
| 30 min | ENSRNOG00000014548 | Nedd9 | 1.25 | 6.83 | 1.87E-13 | 1.84E-11 |
| 30 min | ENSRNOG00000030416 | Zfp709l2 | -1.07 | 3.42 | 6.28E-13 | 6.03E-11 |
| 30 min | ENSRNOG00000000471 | B3galt4 | -1.68 | 1.49 | 2.15E-11 | 1.91E-09 |
| 30 min | ENSRNOG00000015196 | D3ZUB3_RAT | 1.67 | 1.52 | 3.34E-11 | 2.89E-09 |
| 30 min | ENSRNOG00000013092 | D3ZER0_RAT | 1.19 | 6.10 | 5.51E-11 | 4.54E-09 |
| 30 min | ENSRNOG00000004084 | Fam84a | 1.12 | 8.13 | 8.15E-11 | 6.57E-09 |
| 30 min | ENSRNOG00000005037 | Kif18a | 2.32 | 0.23 | 1.30E-10 | 1.03E-08 |
| 30 min | ENSRNOG00000030517 | RGD1563273 | -1.18 | 2.42 | 1.79E-10 | 1.41E-08 |
| 30 min | ENSRNOG00000014205 | Klf2 | 1.81 | 1.90 | 2.24E-10 | 1.75E-08 |
| 30 min | ENSRNOG00000015416 | Obfc2a | 1.02 | 4.22 | 3.54E-10 | 2.73E-08 |
| 30 min | ENSRNOG00000001428 | Alkbh4 | -1.14 | 2.76 | 2.52E-09 | 1.76E-07 |
| 30 min | ENSRNOG00000045465 | SNORD113 | 1.45 | 1.10 | 2.06E-08 | 1.29E-06 |
| 30 min | ENSRNOG00000020325 | Calhm2 | -1.39 | 2.56 | 3.38E-08 | 2.04E-06 |
| 30 min | ENSRNOG00000021628 | Wdr89 | -1.01 | 2.11 | 7.32E-08 | 4.28E-06 |
| 30 min | ENSRNOG00000013862 | Dusp2 | 1.89 | 2.38 | 8.77E-08 | 5.08E-06 |
| 30 min | ENSRNOG00000034718 | SNORD113 | 1.54 | 0.92 | 1.20E-07 | 6.80E-06 |
| 30 min | ENSRNOG00000012280 | Ptx3 | 1.81 | 1.59 | 1.52E-07 | 8.51E-06 |
| 30 min | ENSRNOG00000003977 | Dusp1 | 1.33 | 4.90 | 1.96E-07 | 1.08E-05 |
| 30 min | ENSRNOG00000036007 | SNORD113 | 1.95 | -0.33 | 2.26E-07 | 1.22E-05 |
| 30 min | ENSRNOG00000043267 | Ftsj2 | -1.14 | 1.59 | 2.78E-07 | 1.49E-05 |
| 30 min | ENSRNOG00000027445 | D4A242_RAT | 1.29 | 1.63 | 2.84E-07 | 1.51E-05 |
| 30 min | ENSRNOG00000034168 | Gemin7l1 | -1.21 | 1.72 | 2.89E-07 | 1.54E-05 |
| 30 min | ENSRNOG00000015160 | Gem | 1.05 | 2.07 | 1.32E-06 | 6.35E-05 |
| 30 min | ENSRNOG00000002097 | Rasl11b | 1.68 | 3.20 | 1.43E-06 | 6.88E-05 |
| 30 min | ENSRNOG00000014648 | Efnb2 | 1.08 | 3.17 | 2.61E-06 | 0.000120388 |
| 30 min | ENSRNOG00000023527 | F1M621_RAT | 1.33 | 0.72 | 2.80E-06 | 0.000126773 |
| 30 min | ENSRNOG00000041873 | SNORD113 | 1.30 | 0.69 | 5.35E-06 | 0.000233167 |
| 30 min | ENSRNOG00000042717 | RGD1566380 | 1.07 | 3.14 | 6.03E-06 | 0.000259434 |
| 30 min | ENSRNOG00000009540 | Gpr3 | 2.00 | 0.94 | 6.89E-06 | 0.000291171 |
| 30 min | ENSRNOG00000023055 | D3Z8S2_RAT | 1.00 | 1.82 | 7.69E-06 | 0.000321733 |
| 30 min | ENSRNOG00000028278 | RGD1562655 | 1.41 | 0.80 | 1.32E-05 | 0.000527317 |
| 30 min | ENSRNOG00000034598 | U2 | 1.29 | 0.13 | 1.41E-05 | 0.000556856 |
| 30 min | ENSRNOG00000030034 | Sox11 | 1.06 | 4.89 | 1.47E-05 | 0.000571428 |
| 30 min | ENSRNOG00000040690 | SNORD86 | 1.57 | -0.24 | 1.46E-05 | 0.000571428 |
| 30 min | ENSRNOG00000035909 | U2 | 1.56 | 0.03 | 1.81E-05 | 0.000692015 |
| 30 min | ENSRNOG00000034978 | SNORD113 | 1.78 | -0.56 | 1.82E-05 | 0.000693792 |
| 30 min | ENSRNOG00000010158 | Magel2 | 1.27 | 1.08 | 3.56E-05 | 0.001292751 |
| 30 min | ENSRNOG00000018692 | Mc4r | -1.46 | 1.19 | 4.18E-05 | 0.001491477 |
| 30 min | ENSRNOG00000021266 | Prokr2 | 2.98 | 0.22 | 4.52E-05 | 0.001588523 |
| 30 min | ENSRNOG00000027030 | Adm | 1.49 | 0.48 | 8.12E-05 | 0.002711461 |
| 30 min | ENSRNOG00000043031 | RGD1305627 | 1.58 | 3.39 | 9.83E-05 | 0.003191828 |
| 30 min | ENSRNOG00000037102 | F1M724_RAT | 1.37 | 0.39 | 0.000137857 | 0.004295937 |
| 30 min | ENSRNOG00000035032 | U5 | 1.01 | 0.88 | 0.000165046 | 0.005009617 |
| 30 min | ENSRNOG00000010516 | Plau | -1.38 | 1.64 | 0.000273864 | 0.007696176 |
| 30 min | ENSRNOG00000035442 | SNORD113 | 1.52 | 0.94 | 0.000493364 | 0.01276465 |
| 30 min | ENSRNOG00000008785 | Klf5 | 1.05 | 1.24 | 0.000597784 | 0.015076856 |
| 30 min | ENSRNOG00000035189 | SNORD113 | 1.94 | -0.26 | 0.000643722 | 0.016005176 |
| 30 min | ENSRNOG00000034443 | SNORD113 | 1.16 | 1.04 | 0.000661352 | 0.016353247 |
| 30 min | ENSRNOG00000009157 | Fut4 | -1.18 | 0.42 | 0.000721871 | 0.017543837 |
| 30 min | ENSRNOG00000034534 | SNORD113 | 1.21 | 0.54 | 0.000741836 | 0.017935812 |
| 30 min | ENSRNOG00000004531 | Tceanc | -1.24 | -0.21 | 0.001210241 | 0.027045393 |
| 30 min | ENSRNOG00000004762 | D3ZH63_RAT | -3.03 | 0.11 | 0.001281001 | 0.028423217 |
| 30 min | ENSRNOG00000035576 | rno-mir-27b | 1.18 | 0.06 | 0.001532761 | 0.032665518 |
| 30 min | ENSRNOG00000034642 | SNORD113 | 1.23 | -0.02 | 0.001592092 | 0.03365998 |
| 30 min | ENSRNOG00000023465 | LOC500300 | 1.39 | 1.10 | 0.001605036 | 0.03374788 |
| 30 min | ENSRNOG00000039315 | LOC499781 | 1.49 | -0.42 | 0.001834219 | 0.037760097 |
| 30 min | ENSRNOG00000031540 | retrotransposed | 1.10 | 0.04 | 0.001913714 | 0.039176661 |
| 30 min | ENSRNOG00000035568 | rno-mir-24-1 | 1.24 | -0.32 | 0.001976791 | 0.040135504 |
| 30 min | ENSRNOG00000023352 | Fam78a | -1.13 | 0.50 | 0.002245311 | 0.044013781 |
| 30 min | ENSRNOG00000007000 | Grhl2 | 1.62 | -0.51 | 0.002518774 | 0.047990173 |
| 2 h | ENSRNOG00000000529 | Pim1 | 4.49 | 4.26 | 5.79E-258 | 8.12E-254 |
| 2 h | ENSRNOG00000011387 | Tet3 | 2.83 | 6.63 | 2.23E-193 | 1.56E-189 |
| 2 h | ENSRNOG00000043168 | Homer1 | 3.50 | 6.70 | 1.15E-180 | 5.36E-177 |
| 2 h | ENSRNOG00000007346 | Grasp | 3.45 | 6.62 | 1.23E-167 | 4.30E-164 |
| 2 h | ENSRNOG00000016811 | Fosb | 5.28 | 5.06 | 1.87E-148 | 5.26E-145 |
| 2 h | ENSRNOG00000043465 | Arc | 6.64 | 9.91 | 9.07E-148 | 2.12E-144 |
| 2 h | ENSRNOG00000001006 | Nptx2 | 4.37 | 7.11 | 4.60E-145 | 9.22E-142 |
| 2 h | ENSRNOG00000014320 | Inhba | 6.78 | 5.21 | 4.65E-144 | 8.15E-141 |
| 2 h | ENSRNOG00000019822 | Gadd45b | 4.08 | 6.55 | 6.95E-143 | 1.08E-139 |
| 2 h | ENSRNOG00000033433 | Csrnp1 | 3.33 | 4.94 | 1.08E-140 | 1.51E-137 |
| 2 h | ENSRNOG00000005561 | Dbc1 | 1.48 | 8.50 | 6.09E-133 | 7.76E-130 |
| 2 h | ENSRNOG00000017828 | Egr3 | 4.02 | 8.00 | 6.89E-127 | 8.06E-124 |
| 2 h | ENSRNOG00000023509 | Irs2 | 2.75 | 7.01 | 3.31E-125 | 3.57E-122 |
| 2 h | ENSRNOG00000000130 | Dnajb5 | 2.64 | 7.97 | 4.18E-124 | 4.19E-121 |
| 2 h | ENSRNOG00000004100 | Trib1 | 4.36 | 4.60 | 8.98E-120 | 8.39E-117 |
| 2 h | ENSRNOG00000012258 | Rras2 | 2.29 | 5.65 | 8.55E-118 | 7.49E-115 |
| 2 h | ENSRNOG00000010058 | Spry2 | 3.67 | 5.96 | 6.77E-117 | 5.58E-114 |
| 2 h | ENSRNOG00000043498 | Sik2 | 2.05 | 6.20 | 8.93E-114 | 6.96E-111 |
| 2 h | ENSRNOG00000000640 | Egr2 | 5.31 | 5.24 | 1.14E-109 | 8.40E-107 |
| 2 h | ENSRNOG00000020770 | Arl4d | 3.54 | 5.67 | 1.30E-108 | 9.12E-106 |
| 2 h | ENSRNOG00000015401 | Mapk4 | 2.31 | 5.77 | 5.36E-97 | 3.58E-94 |
| 2 h | ENSRNOG00000010888 | Ankrd33b | 2.50 | 5.54 | 3.32E-94 | 2.12E-91 |
| 2 h | ENSRNOG00000024363 | Sertad1 | 2.99 | 4.53 | 6.25E-91 | 3.81E-88 |
| 2 h | ENSRNOG00000023446 | LOC689986 | 1.68 | 7.59 | 2.88E-86 | 1.68E-83 |
| 2 h | ENSRNOG00000000635 | Arid5b | 2.21 | 5.82 | 6.86E-85 | 3.85E-82 |
| 2 h | ENSRNOG00000013520 | RGD1560523 | 1.99 | 5.79 | 7.28E-83 | 3.92E-80 |
| 2 h | ENSRNOG00000001416 | Vgf | 3.29 | 6.77 | 2.30E-82 | 1.19E-79 |
| 2 h | ENSRNOG00000008415 | Nab2 | 2.31 | 5.88 | 2.98E-82 | 1.49E-79 |
| 2 h | ENSRNOG00000031167 | Srxn1 | 2.70 | 4.94 | 5.82E-80 | 2.81E-77 |
| 2 h | ENSRNOG00000002525 | Ptgs2 | 4.67 | 9.04 | 1.00E-77 | 4.68E-75 |
| 2 h | ENSRNOG00000037613 | Kdm6b | 3.38 | 5.36 | 1.90E-75 | 8.61E-73 |
| 2 h | ENSRNOG00000016326 | Cx3cl1 | 1.87 | 8.97 | 7.22E-74 | 3.16E-71 |
| 2 h | ENSRNOG00000009821 | Slitrk3 | 2.01 | 6.53 | 7.76E-74 | 3.30E-71 |
| 2 h | ENSRNOG00000013101 | PCDH8_RAT | 4.09 | 8.53 | 1.25E-72 | 5.15E-70 |
| 2 h | ENSRNOG00000006329 | Peli1 | 1.90 | 5.42 | 2.08E-70 | 8.32E-68 |
| 2 h | ENSRNOG00000013945 | Itpkc | 1.91 | 4.66 | 4.00E-70 | 1.56E-67 |
| 2 h | ENSRNOG00000014061 | Dusp5 | 4.89 | 4.30 | 4.34E-70 | 1.65E-67 |
| 2 h | ENSRNOG00000016180 | Pdp1 | 1.99 | 6.75 | 3.04E-69 | 1.12E-66 |
| 2 h | ENSRNOG00000024114 | D4A582_RAT | 2.21 | 7.02 | 1.53E-67 | 5.48E-65 |
| 2 h | ENSRNOG00000003687 | Rgs2 | 2.03 | 6.20 | 1.24E-66 | 4.33E-64 |
| 2 h | ENSRNOG00000004049 | Baiap2 | 1.94 | 8.28 | 5.96E-65 | 2.04E-62 |
| 2 h | ENSRNOG00000006118 | Klf10 | 2.49 | 6.56 | 2.11E-64 | 7.06E-62 |
| 2 h | ENSRNOG00000011921 | Dusp4 | 3.02 | 5.32 | 7.67E-63 | 2.50E-60 |
| 2 h | ENSRNOG00000026880 | D3Z8K5_RAT | 1.64 | 5.99 | 2.11E-61 | 6.74E-59 |
| 2 h | ENSRNOG00000035476 | rno-mir-212 | 6.65 | 1.34 | 3.41E-61 | 1.06E-58 |
| 2 h | ENSRNOG00000015415 | Rhoq | 2.32 | 5.05 | 2.10E-60 | 6.40E-58 |
| 2 h | ENSRNOG00000025372 | Glce | 1.70 | 5.61 | 2.77E-60 | 8.25E-58 |
| 2 h | ENSRNOG00000011951 | Plk2 | 3.21 | 8.58 | 3.31E-60 | 9.66E-58 |
| 2 h | ENSRNOG00000019181 | Synpo | 2.39 | 7.12 | 1.00E-58 | 2.87E-56 |
| 2 h | ENSRNOG00000003745 | Atf3 | 6.76 | 5.03 | 4.28E-57 | 1.20E-54 |
| 2 h | ENSRNOG00000042188 | ENSRNOG00000042188 | 2.10 | 5.75 | 7.44E-57 | 2.05E-54 |
| 2 h | ENSRNOG00000018359 | Smad7 | 2.12 | 5.77 | 8.11E-56 | 2.19E-53 |
| 2 h | ENSRNOG00000005393 | Sertad2 | 1.92 | 4.03 | 2.12E-55 | 5.62E-53 |
| 2 h | ENSRNOG00000002657 | Pla2g4a | 2.30 | 3.87 | 1.06E-54 | 2.74E-52 |
| 2 h | ENSRNOG00000012442 | RGD1305254 | 4.32 | 6.49 | 1.83E-53 | 4.66E-51 |
| 2 h | ENSRNOG00000001189 | Sik1 | 2.83 | 5.54 | 6.21E-53 | 1.56E-50 |
| 2 h | ENSRNOG00000014528 | F1M4G7_RAT | 1.47 | 5.63 | 7.62E-53 | 1.87E-50 |
| 2 h | ENSRNOG00000042838 | Junb | 2.43 | 7.29 | 1.33E-52 | 3.22E-50 |
| 2 h | ENSRNOG00000010258 | Vhl | 1.40 | 6.16 | 2.36E-51 | 5.60E-49 |
| 2 h | ENSRNOG00000012623 | Arf4 | 1.35 | 7.39 | 1.17E-50 | 2.74E-48 |
| 2 h | ENSRNOG00000006000 | CDK12_RAT | 1.36 | 6.35 | 1.38E-50 | 3.16E-48 |
| 2 h | ENSRNOG00000006789 | Ddit3 | 2.37 | 5.97 | 1.50E-50 | 3.39E-48 |
| 2 h | ENSRNOG00000015719 | Egr4 | 4.49 | 4.69 | 2.10E-50 | 4.68E-48 |
| 2 h | ENSRNOG00000009046 | Phf13 | 1.93 | 3.82 | 3.07E-49 | 6.72E-47 |
| 2 h | ENSRNOG00000018107 | Zfand5 | 1.30 | 8.02 | 4.02E-49 | 8.54E-47 |
| 2 h | ENSRNOG00000019422 | Egr1 | 2.29 | 6.61 | 3.96E-49 | 8.54E-47 |
| 2 h | ENSRNOG00000022772 | Prickle1 | 1.97 | 8.03 | 6.06E-49 | 1.27E-46 |
| 2 h | ENSRNOG00000007892 | Tram1 | 1.16 | 6.97 | 2.07E-48 | 4.27E-46 |
| 2 h | ENSRNOG00000006828 | F1M4U9_RAT | 2.86 | 4.42 | 3.81E-48 | 7.75E-46 |
| 2 h | ENSRNOG00000008919 | Arpp21 | 2.51 | 8.42 | 2.78E-47 | 5.57E-45 |
| 2 h | ENSRNOG00000000956 | Rasl11a | 3.37 | 3.03 | 3.39E-47 | 6.69E-45 |
| 2 h | ENSRNOG00000000648 | Jmjd1c | 1.53 | 7.97 | 3.95E-47 | 7.66E-45 |
| 2 h | ENSRNOG00000002129 | Lrrc8b | 1.20 | 7.66 | 3.99E-47 | 7.66E-45 |
| 2 h | ENSRNOG00000005600 | Nr4a2 | 3.28 | 6.72 | 6.80E-47 | 1.29E-44 |
| 2 h | ENSRNOG00000039902 | LOC683626 | 2.24 | 4.90 | 1.56E-46 | 2.91E-44 |
| 2 h | ENSRNOG00000000781 | Rnf39 | 3.06 | 5.61 | 2.26E-46 | 4.16E-44 |
| 2 h | ENSRNOG00000002946 | Socs3 | 3.91 | 0.72 | 6.47E-46 | 1.18E-43 |
| 2 h | ENSRNOG00000001785 | Etv5 | 1.61 | 6.14 | 2.78E-45 | 4.99E-43 |
| 2 h | ENSRNOG00000006832 | Zdhhc5 | 1.10 | 6.22 | 3.34E-45 | 5.94E-43 |
| 2 h | ENSRNOG00000010712 | Terf2ip | 1.63 | 6.90 | 4.32E-45 | 7.57E-43 |
| 2 h | ENSRNOG00000037615 | ENSRNOG00000037615 | 3.21 | 2.85 | 4.99E-45 | 8.64E-43 |
| 2 h | ENSRNOG00000036662 | Wdr45l | 1.29 | 5.66 | 7.36E-45 | 1.26E-42 |
| 2 h | ENSRNOG00000013339 | Gmeb2 | 1.49 | 4.23 | 1.54E-44 | 2.60E-42 |
| 2 h | ENSRNOG00000009540 | Gpr3 | 4.67 | 0.94 | 6.39E-44 | 1.07E-41 |
| 2 h | ENSRNOG00000015691 | RGD1306526 | 1.07 | 8.05 | 3.79E-43 | 6.26E-41 |
| 2 h | ENSRNOG00000005157 | Rbbp7 | 1.29 | 7.99 | 7.64E-43 | 1.25E-40 |
| 2 h | ENSRNOG00000007478 | Cry2 | 1.24 | 7.31 | 1.69E-42 | 2.73E-40 |
| 2 h | ENSRNOG00000010208 | Timp1 | 4.59 | 3.61 | 5.19E-42 | 8.28E-40 |
| 2 h | ENSRNOG00000010060 | Panx1 | 1.67 | 4.79 | 1.12E-41 | 1.77E-39 |
| 2 h | ENSRNOG00000005285 | Fbxo33 | 1.49 | 6.90 | 1.55E-41 | 2.41E-39 |
| 2 h | ENSRNOG00000003121 | Rtn4rl1 | 1.88 | 8.06 | 7.75E-41 | 1.19E-38 |
| 2 h | ENSRNOG00000003329 | Ppm1d | 1.63 | 5.76 | 1.01E-40 | 1.54E-38 |
| 2 h | ENSRNOG00000011704 | Fbxo34 | 1.12 | 6.21 | 1.40E-40 | 2.11E-38 |
| 2 h | ENSRNOG00000004807 | Arf2 | 1.29 | 5.10 | 2.44E-40 | 3.64E-38 |
| 2 h | ENSRNOG00000013916 | Nefm | 1.50 | 8.16 | 2.61E-40 | 3.85E-38 |
| 2 h | ENSRNOG00000015547 | Jak2 | 1.18 | 6.09 | 3.65E-40 | 5.33E-38 |
| 2 h | ENSRNOG00000010889 | D3ZQU4_RAT | 1.27 | 7.14 | 7.20E-40 | 1.04E-37 |
| 2 h | ENSRNOG00000002159 | Agpat9 | 1.84 | 3.21 | 2.22E-39 | 3.18E-37 |
| 2 h | ENSRNOG00000017212 | Spsb1 | 1.38 | 4.64 | 9.75E-39 | 1.38E-36 |
| 2 h | ENSRNOG00000018484 | Plk3 | 2.79 | 3.77 | 1.61E-38 | 2.26E-36 |
| 2 h | ENSRNOG00000018911 | Pfkfb3 | 1.47 | 5.69 | 2.39E-38 | 3.32E-36 |
| 2 h | ENSRNOG00000009525 | Rnf114 | 1.05 | 5.64 | 2.49E-38 | 3.42E-36 |
| 2 h | ENSRNOG00000012934 | Arhgef7 | 1.13 | 7.38 | 2.63E-38 | 3.58E-36 |
| 2 h | ENSRNOG00000003019 | Ubn1 | 1.08 | 6.46 | 5.57E-38 | 7.51E-36 |
| 2 h | ENSRNOG00000005140 | Prmt8 | 1.09 | 6.03 | 1.44E-37 | 1.90E-35 |
| 2 h | ENSRNOG00000020061 | LOC100365924 | 1.65 | 5.22 | 1.43E-37 | 1.90E-35 |
| 2 h | ENSRNOG00000015382 | Arid5a | 2.44 | 4.66 | 1.84E-37 | 2.42E-35 |
| 2 h | ENSRNOG00000033906 | Znf667 | 1.31 | 5.84 | 3.29E-37 | 4.27E-35 |
| 2 h | ENSRNOG00000009186 | Stmn4 | 1.17 | 7.98 | 3.50E-37 | 4.50E-35 |
| 2 h | ENSRNOG00000019229 | Fbl | 1.83 | 5.29 | 5.03E-37 | 6.41E-35 |
| 2 h | ENSRNOG00000010744 | Nrp1 | 1.86 | 7.45 | 5.52E-37 | 6.98E-35 |
| 2 h | ENSRNOG00000007152 | Bhlhe40 | 3.16 | 6.43 | 9.36E-37 | 1.17E-34 |
| 2 h | ENSRNOG00000010765 | Vcl | 1.54 | 6.25 | 1.86E-36 | 2.31E-34 |
| 2 h | ENSRNOG00000008015 | Fos | 4.12 | 6.22 | 3.65E-36 | 4.49E-34 |
| 2 h | ENSRNOG00000017938 | Btaf1 | 1.31 | 6.52 | 6.75E-36 | 8.23E-34 |
| 2 h | ENSRNOG00000006184 | Pmepa1 | 1.43 | 5.76 | 2.13E-35 | 2.53E-33 |
| 2 h | ENSRNOG00000010720 | Mast4 | 1.61 | 7.08 | 5.91E-34 | 6.91E-32 |
| 2 h | ENSRNOG00000016122 | Hmgcr | 1.19 | 7.53 | 6.96E-34 | 8.07E-32 |
| 2 h | ENSRNOG00000001730 | Acap2 | 1.14 | 7.24 | 5.64E-33 | 6.49E-31 |
| 2 h | ENSRNOG00000043095 | Etv3 | 1.34 | 4.07 | 8.81E-33 | 1.00E-30 |
| 2 h | ENSRNOG00000002075 | Cnot6l | 1.50 | 5.85 | 1.12E-32 | 1.26E-30 |
| 2 h | ENSRNOG00000021513 | Rtn4rl2 | 2.24 | 5.24 | 1.25E-32 | 1.40E-30 |
| 2 h | ENSRNOG00000025764 | Mt1a | 2.12 | 5.02 | 1.89E-32 | 2.09E-30 |
| 2 h | ENSRNOG00000005964 | Nr4a3 | 1.91 | 6.84 | 3.42E-32 | 3.75E-30 |
| 2 h | ENSRNOG00000030034 | Sox11 | 2.91 | 4.89 | 7.34E-32 | 7.92E-30 |
| 2 h | ENSRNOG00000015631 | Ippk | 1.48 | 5.25 | 1.07E-31 | 1.15E-29 |
| 2 h | ENSRNOG00000010428 | Bahd1 | 1.05 | 5.42 | 1.12E-31 | 1.19E-29 |
| 2 h | ENSRNOG00000018520 | Nsun6 | 1.65 | 2.67 | 1.43E-31 | 1.51E-29 |
| 2 h | ENSRNOG00000038440 | D3ZF40_RAT | 2.48 | 1.47 | 1.61E-31 | 1.68E-29 |
| 2 h | ENSRNOG00000019742 | Stat3 | 1.07 | 6.38 | 1.81E-31 | 1.88E-29 |
| 2 h | ENSRNOG00000013658 | Nefl | 1.11 | 10.31 | 1.85E-31 | 1.91E-29 |
| 2 h | ENSRNOG00000019645 | Osbp2 | 1.21 | 7.47 | 5.57E-31 | 5.70E-29 |
| 2 h | ENSRNOG00000015801 | Spns2 | 1.21 | 6.57 | 5.63E-31 | 5.72E-29 |
| 2 h | ENSRNOG00000014363 | Arhgef3 | 1.33 | 7.38 | 5.82E-31 | 5.87E-29 |
| 2 h | ENSRNOG00000021540 | RGD1563441 | 1.55 | 6.36 | 6.11E-31 | 6.12E-29 |
| 2 h | ENSRNOG00000014886 | Fam107b | 1.75 | 5.21 | 7.44E-31 | 7.40E-29 |
| 2 h | ENSRNOG00000003675 | Ptchd1 | 2.31 | 3.90 | 8.97E-31 | 8.86E-29 |
| 2 h | ENSRNOG00000007128 | Nop56 | 1.06 | 6.78 | 1.06E-30 | 1.03E-28 |
| 2 h | ENSRNOG00000012500 | Ddx10 | 1.11 | 5.85 | 1.55E-30 | 1.51E-28 |
| 2 h | ENSRNOG00000007029 | Dnaja1 | 1.02 | 8.66 | 1.99E-30 | 1.92E-28 |
| 2 h | ENSRNOG00000005209 | Spred1 | 1.01 | 6.03 | 2.47E-30 | 2.38E-28 |
| 2 h | ENSRNOG00000033026 | Dclk3 | -2.85 | 3.28 | 3.48E-30 | 3.32E-28 |
| 2 h | ENSRNOG00000020938 | Ppp1r15a | 1.28 | 5.64 | 4.03E-30 | 3.82E-28 |
| 2 h | ENSRNOG00000032178 | Cenpa | 5.28 | 0.03 | 7.84E-30 | 7.38E-28 |
| 2 h | ENSRNOG00000007582 | Zswim4 | 1.20 | 4.36 | 1.04E-29 | 9.61E-28 |
| 2 h | ENSRNOG00000019354 | Ptch1 | 1.11 | 5.43 | 7.44E-29 | 6.77E-27 |
| 2 h | ENSRNOG00000002345 | LOC100361238 | 1.97 | 4.43 | 9.12E-29 | 8.25E-27 |
| 2 h | ENSRNOG00000004624 | Rnd3 | 1.77 | 4.81 | 9.63E-29 | 8.66E-27 |
| 2 h | ENSRNOG00000007387 | Per1 | 1.93 | 6.48 | 1.25E-28 | 1.11E-26 |
| 2 h | ENSRNOG00000021687 | RGD1564664 | 2.40 | 4.63 | 1.60E-28 | 1.41E-26 |
| 2 h | ENSRNOG00000020937 | Ubtf | 1.00 | 7.60 | 1.85E-28 | 1.62E-26 |
| 2 h | ENSRNOG00000017087 | Man1c1 | 1.44 | 5.50 | 2.18E-28 | 1.90E-26 |
| 2 h | ENSRNOG00000011238 | D3ZMH5_RAT | 1.57 | 6.60 | 2.75E-28 | 2.37E-26 |
| 2 h | ENSRNOG00000017193 | Lingo1 | 1.64 | 7.37 | 2.83E-28 | 2.42E-26 |
| 2 h | ENSRNOG00000020009 | Npas4 | 4.82 | 4.36 | 3.10E-28 | 2.63E-26 |
| 2 h | ENSRNOG00000018149 | Smarca5 | 1.01 | 7.57 | 3.98E-28 | 3.36E-26 |
| 2 h | ENSRNOG00000007483 | Ccnf | 2.07 | 2.88 | 4.37E-28 | 3.67E-26 |
| 2 h | ENSRNOG00000004309 | Actn1 | 1.05 | 6.60 | 5.55E-28 | 4.63E-26 |
| 2 h | ENSRNOG00000018714 | Arl5b | 1.97 | 5.89 | 1.15E-27 | 9.49E-26 |
| 2 h | ENSRNOG00000004888 | Spred2 | 1.65 | 4.35 | 1.43E-27 | 1.17E-25 |
| 2 h | ENSRNOG00000015434 | Midn | 1.83 | 3.97 | 1.79E-27 | 1.46E-25 |
| 2 h | ENSRNOG00000006663 | Usp2 | 1.49 | 5.69 | 2.29E-27 | 1.84E-25 |
| 2 h | ENSRNOG00000013092 | D3ZER0_RAT | 1.98 | 6.10 | 2.47E-27 | 1.98E-25 |
| 2 h | ENSRNOG00000035516 | rno-mir-132 | 5.82 | -0.07 | 2.58E-27 | 2.05E-25 |
| 2 h | ENSRNOG00000043123 | Rnf128 | 1.34 | 5.43 | 8.63E-27 | 6.80E-25 |
| 2 h | ENSRNOG00000005724 | Map3k7 | 1.10 | 6.48 | 9.44E-27 | 7.39E-25 |
| 2 h | ENSRNOG00000007607 | Nr4a1 | 2.04 | 5.91 | 9.65E-27 | 7.52E-25 |
| 2 h | ENSRNOG00000028513 | ENSRNOG00000028513 | 1.95 | 2.41 | 2.01E-26 | 1.54E-24 |
| 2 h | ENSRNOG00000020426 | Erf | 1.64 | 4.92 | 8.43E-26 | 6.36E-24 |
| 2 h | ENSRNOG00000021584 | LOC100359912 | 1.02 | 5.71 | 9.86E-26 | 7.35E-24 |
| 2 h | ENSRNOG00000038044 | Tsc22d2 | 1.16 | 5.48 | 2.37E-25 | 1.75E-23 |
| 2 h | ENSRNOG00000004697 | BAALC_RAT | 1.11 | 7.46 | 3.35E-25 | 2.43E-23 |
| 2 h | ENSRNOG00000019475 | Sh3bp5 | -1.28 | 6.57 | 7.44E-25 | 5.33E-23 |
| 2 h | ENSRNOG00000017913 | Atg16l1 | 1.05 | 6.05 | 8.51E-25 | 6.06E-23 |
| 2 h | ENSRNOG00000043381 | D4A8L6_RAT | -1.06 | 5.10 | 1.65E-24 | 1.17E-22 |
| 2 h | ENSRNOG00000016950 | Otud1 | 2.62 | 3.13 | 1.71E-24 | 1.21E-22 |
| 2 h | ENSRNOG00000037085 | Xirp1 | 4.18 | 3.21 | 1.89E-24 | 1.33E-22 |
| 2 h | ENSRNOG00000026293 | Jun | 1.96 | 7.49 | 5.17E-24 | 3.56E-22 |
| 2 h | ENSRNOG00000005924 | Dstn | 1.04 | 7.32 | 7.67E-24 | 5.24E-22 |
| 2 h | ENSRNOG00000020597 | RGD1305014 | 1.36 | 4.11 | 1.37E-23 | 9.36E-22 |
| 2 h | ENSRNOG00000028382 | Rfxap | 1.16 | 5.15 | 1.67E-23 | 1.12E-21 |
| 2 h | ENSRNOG00000039274 | ENSRNOG00000039274 | 1.47 | 6.16 | 3.60E-23 | 2.41E-21 |
| 2 h | ENSRNOG00000013862 | Dusp2 | 3.43 | 2.38 | 4.13E-23 | 2.73E-21 |
| 2 h | ENSRNOG00000013067 | Phf21b | 2.45 | 2.49 | 8.09E-23 | 5.32E-21 |
| 2 h | ENSRNOG00000011107 | Pcsk1 | 1.07 | 6.03 | 1.25E-22 | 8.10E-21 |
| 2 h | ENSRNOG00000011582 | Rab3d | -1.29 | 4.50 | 1.49E-22 | 9.58E-21 |
| 2 h | ENSRNOG00000043378 | Nkpd1 | 2.62 | 1.28 | 1.69E-22 | 1.08E-20 |
| 2 h | ENSRNOG00000016571 | Ngf | 3.01 | 3.62 | 1.92E-22 | 1.22E-20 |
| 2 h | ENSRNOG00000021484 | retrotransposed | 2.25 | 1.43 | 1.95E-22 | 1.23E-20 |
| 2 h | ENSRNOG00000016258 | Znf516 | 1.59 | 4.38 | 1.04E-21 | 6.44E-20 |
| 2 h | ENSRNOG00000003805 | Rnft1 | 1.28 | 3.66 | 1.79E-21 | 1.10E-19 |
| 2 h | ENSRNOG00000003348 | Rasd1 | 1.60 | 4.96 | 1.81E-21 | 1.11E-19 |
| 2 h | ENSRNOG00000027540 | Fam102b | 1.01 | 4.49 | 1.94E-21 | 1.18E-19 |
| 2 h | ENSRNOG00000018498 | F1MA03_RAT | 1.39 | 5.39 | 2.72E-21 | 1.65E-19 |
| 2 h | ENSRNOG00000013703 | Siah2 | 1.06 | 4.63 | 5.06E-21 | 3.04E-19 |
| 2 h | ENSRNOG00000002680 | Lamc1 | 1.04 | 6.09 | 6.29E-21 | 3.75E-19 |
| 2 h | ENSRNOG00000004307 | Tor3a | 1.22 | 3.43 | 9.94E-21 | 5.84E-19 |
| 2 h | ENSRNOG00000023896 | Dusp6 | 1.99 | 4.79 | 1.03E-20 | 6.03E-19 |
| 2 h | ENSRNOG00000014548 | Nedd9 | 1.58 | 6.83 | 2.11E-20 | 1.23E-18 |
| 2 h | ENSRNOG00000007126 | Gpr19 | 1.24 | 4.08 | 3.14E-20 | 1.82E-18 |
| 2 h | ENSRNOG00000015416 | Obfc2a | 1.48 | 4.22 | 4.07E-20 | 2.34E-18 |
| 2 h | ENSRNOG00000000488 | Hmga1 | 1.12 | 4.82 | 4.77E-20 | 2.72E-18 |
| 2 h | ENSRNOG00000011668 | Nfil3 | 1.62 | 3.56 | 5.44E-20 | 3.09E-18 |
| 2 h | ENSRNOG00000007329 | Frmd6 | 1.80 | 3.91 | 8.96E-20 | 5.05E-18 |
| 2 h | ENSRNOG00000018949 | Dlk2 | 1.38 | 3.51 | 1.01E-19 | 5.66E-18 |
| 2 h | ENSRNOG00000007031 | Trim9 | 1.03 | 8.20 | 1.77E-19 | 9.76E-18 |
| 2 h | ENSRNOG00000032546 | Dot1l | 1.31 | 4.70 | 2.93E-19 | 1.57E-17 |
| 2 h | ENSRNOG00000020254 | Per2 | 1.38 | 5.20 | 5.81E-19 | 3.04E-17 |
| 2 h | ENSRNOG00000018859 | P3IP1_RAT | -1.01 | 5.38 | 7.39E-19 | 3.85E-17 |
| 2 h | ENSRNOG00000014082 | Sp7 | 3.85 | 0.12 | 1.10E-18 | 5.68E-17 |
| 2 h | ENSRNOG00000006612 | Map2k3 | 1.53 | 3.40 | 1.58E-18 | 8.12E-17 |
| 2 h | ENSRNOG00000032922 | Dclk1 | 1.24 | 9.12 | 2.05E-18 | 1.05E-16 |
| 2 h | ENSRNOG00000025587 | Plagl1 | 1.84 | 2.59 | 4.18E-18 | 2.11E-16 |
| 2 h | ENSRNOG00000006670 | Rai2 | 1.63 | 2.92 | 4.86E-18 | 2.43E-16 |
| 2 h | ENSRNOG00000025804 | Fastkd5 | 1.40 | 3.60 | 5.82E-18 | 2.90E-16 |
| 2 h | ENSRNOG00000014900 | CREM_RAT | 1.13 | 3.85 | 7.76E-18 | 3.80E-16 |
| 2 h | ENSRNOG00000038047 | LOC100362769 | 1.99 | 2.00 | 8.87E-18 | 4.32E-16 |
| 2 h | ENSRNOG00000009253 | Igsf9b | 1.93 | 4.40 | 9.22E-18 | 4.47E-16 |
| 2 h | ENSRNOG00000018692 | Mc4r | -2.60 | 1.19 | 3.41E-17 | 1.63E-15 |
| 2 h | ENSRNOG00000000275 | D3ZD52_RAT | 1.13 | 5.80 | 9.90E-17 | 4.57E-15 |
| 2 h | ENSRNOG00000018275 | Errfi1 | 1.10 | 6.69 | 1.43E-16 | 6.52E-15 |
| 2 h | ENSRNOG00000042546 | RGD1562551 | 1.42 | 2.51 | 2.58E-16 | 1.15E-14 |
| 2 h | ENSRNOG00000014456 | Coq10b | 1.29 | 5.08 | 3.95E-16 | 1.75E-14 |
| 2 h | ENSRNOG00000003909 | Esyt1 | 1.58 | 3.55 | 9.70E-16 | 4.21E-14 |
| 2 h | ENSRNOG00000026328 | RGD1563070 | 1.23 | 3.74 | 9.88E-16 | 4.28E-14 |
| 2 h | ENSRNOG00000008067 | LOC100363014 | 1.17 | 3.96 | 1.34E-15 | 5.78E-14 |
| 2 h | ENSRNOG00000002122 | Lrrc8c | 1.20 | 3.30 | 1.50E-15 | 6.46E-14 |
| 2 h | ENSRNOG00000028099 | retrotransposed | 1.98 | 0.69 | 1.84E-15 | 7.90E-14 |
| 2 h | ENSRNOG00000030101 | Traip | -1.62 | 3.12 | 2.42E-15 | 1.03E-13 |
| 2 h | ENSRNOG00000034240 | D4A1S3_RAT | 1.18 | 5.15 | 2.89E-15 | 1.22E-13 |
| 2 h | ENSRNOG00000002336 | D3ZMK4_RAT | 1.01 | 7.32 | 3.98E-15 | 1.64E-13 |
| 2 h | ENSRNOG00000008533 | Eif2c2 | 1.07 | 6.36 | 5.67E-15 | 2.32E-13 |
| 2 h | ENSRNOG00000011631 | Fst | 2.96 | 0.40 | 1.00E-14 | 4.07E-13 |
| 2 h | ENSRNOG00000005353 | Rybp | 1.00 | 5.16 | 1.06E-14 | 4.31E-13 |
| 2 h | ENSRNOG00000010489 | SMAG1_RAT | 1.33 | 5.35 | 1.16E-14 | 4.66E-13 |
| 2 h | ENSRNOG00000038004 | Znf804a | 1.65 | 4.80 | 1.58E-14 | 6.28E-13 |
| 2 h | ENSRNOG00000005148 | Scrt2 | 1.83 | 1.32 | 1.81E-14 | 7.16E-13 |
| 2 h | ENSRNOG00000028992 | Acan | 1.13 | 3.97 | 1.83E-14 | 7.23E-13 |
| 2 h | ENSRNOG00000001141 | Srrm4 | 1.01 | 3.57 | 2.18E-14 | 8.57E-13 |
| 2 h | ENSRNOG00000023534 | Haus8 | 2.07 | 3.76 | 2.59E-14 | 1.01E-12 |
| 2 h | ENSRNOG00000010488 | Zmiz1 | 1.23 | 6.63 | 2.69E-14 | 1.05E-12 |
| 2 h | ENSRNOG00000000748 | Dstnl1 | 1.04 | 3.78 | 2.87E-14 | 1.12E-12 |
| 2 h | ENSRNOG00000003696 | Prkx | 1.31 | 4.30 | 2.89E-14 | 1.12E-12 |
| 2 h | ENSRNOG00000024886 | Ext1 | 1.15 | 4.71 | 3.01E-14 | 1.16E-12 |
| 2 h | ENSRNOG00000000777 | RT1-S3 | 1.64 | 3.73 | 4.20E-14 | 1.60E-12 |
| 2 h | ENSRNOG00000019533 | Klhl3 | 1.12 | 4.79 | 5.80E-14 | 2.19E-12 |
| 2 h | ENSRNOG00000006859 | Insig1 | 1.11 | 7.21 | 5.96E-14 | 2.24E-12 |
| 2 h | ENSRNOG00000014971 | Mas1 | 1.65 | 6.41 | 2.89E-13 | 1.05E-11 |
| 2 h | ENSRNOG00000007302 | Fbn1 | -1.66 | 5.24 | 3.46E-13 | 1.25E-11 |
| 2 h | ENSRNOG00000020325 | Calhm2 | -1.87 | 2.56 | 3.64E-13 | 1.31E-11 |
| 2 h | ENSRNOG00000019598 | Vegfa | 1.14 | 6.04 | 3.68E-13 | 1.33E-11 |
| 2 h | ENSRNOG00000016541 | Enc1 | 1.17 | 10.18 | 4.45E-13 | 1.60E-11 |
| 2 h | ENSRNOG00000020298 | Bag3 | 1.60 | 5.32 | 7.96E-13 | 2.81E-11 |
| 2 h | ENSRNOG00000005302 | Slc2a9 | 1.87 | 2.63 | 8.68E-13 | 3.05E-11 |
| 2 h | ENSRNOG00000042191 | Rcor1 | 1.12 | 4.06 | 1.95E-12 | 6.69E-11 |
| 2 h | ENSRNOG00000003813 | Kcnk10 | 1.63 | 4.15 | 2.01E-12 | 6.86E-11 |
| 2 h | ENSRNOG00000012991 | LOC100363275 | 1.04 | 3.78 | 2.12E-12 | 7.25E-11 |
| 2 h | ENSRNOG00000043031 | RGD1305627 | 2.83 | 3.39 | 2.48E-12 | 8.35E-11 |
| 2 h | ENSRNOG00000016690 | Idi1 | 1.19 | 5.80 | 3.80E-12 | 1.27E-10 |
| 2 h | ENSRNOG00000011513 | Gla | 1.02 | 5.32 | 4.22E-12 | 1.41E-10 |
| 2 h | ENSRNOG00000019370 | Znf697 | 1.00 | 3.95 | 5.89E-12 | 1.96E-10 |
| 2 h | ENSRNOG00000018938 | Mkx | -2.40 | 2.60 | 6.31E-12 | 2.08E-10 |
| 2 h | ENSRNOG00000008943 | Penk | 1.70 | 5.90 | 1.55E-11 | 4.97E-10 |
| 2 h | ENSRNOG00000033973 | RGD1561115 | -1.54 | 2.48 | 1.85E-11 | 5.87E-10 |
| 2 h | ENSRNOG00000016110 | Kcnk12 | 1.44 | 2.65 | 2.13E-11 | 6.70E-10 |
| 2 h | ENSRNOG00000037690 | Sertad3 | 1.45 | 1.59 | 2.90E-11 | 9.06E-10 |
| 2 h | ENSRNOG00000044426 | U6 | 1.35 | 1.86 | 3.74E-11 | 1.15E-09 |
| 2 h | ENSRNOG00000017260 | Cdr2 | 1.18 | 3.11 | 3.94E-11 | 1.21E-09 |
| 2 h | ENSRNOG00000010557 | Smarcd2 | -1.14 | 5.17 | 9.63E-11 | 2.89E-09 |
| 2 h | ENSRNOG00000028017 | Tmem109 | -1.01 | 4.10 | 1.09E-10 | 3.26E-09 |
| 2 h | ENSRNOG00000015488 | Tead1 | 1.04 | 3.75 | 1.15E-10 | 3.45E-09 |
| 2 h | ENSRNOG00000002202 | Ankrd56 | 3.07 | 1.72 | 1.21E-10 | 3.61E-09 |
| 2 h | ENSRNOG00000035620 | rno-mir-22 | 1.20 | 1.86 | 1.29E-10 | 3.83E-09 |
| 2 h | ENSRNOG00000008445 | D4A3W8_RAT | 1.39 | 2.11 | 2.32E-10 | 6.71E-09 |
| 2 h | ENSRNOG00000030382 | ENSRNOG00000030382 | -2.03 | 1.29 | 4.75E-10 | 1.34E-08 |
| 2 h | ENSRNOG00000027002 | D4A8R8_RAT | 1.25 | 1.79 | 5.25E-10 | 1.47E-08 |
| 2 h | ENSRNOG00000030517 | RGD1563273 | -1.11 | 2.42 | 5.52E-10 | 1.53E-08 |
| 2 h | ENSRNOG00000024245 | D3ZXU0_RAT | -1.03 | 2.87 | 8.49E-10 | 2.33E-08 |
| 2 h | ENSRNOG00000030452 | Rimbp3 | -1.39 | 1.75 | 1.28E-09 | 3.43E-08 |
| 2 h | ENSRNOG00000012989 | Serinc2 | 1.83 | 3.87 | 1.34E-09 | 3.58E-08 |
| 2 h | ENSRNOG00000013851 | Spry4 | 1.35 | 2.83 | 1.38E-09 | 3.67E-08 |
| 2 h | ENSRNOG00000022730 | Zfp57 | -1.05 | 3.76 | 1.47E-09 | 3.89E-08 |
| 2 h | ENSRNOG00000036699 | RGD1306926 | -1.29 | 4.21 | 1.83E-09 | 4.82E-08 |
| 2 h | ENSRNOG00000013171 | Grm2 | -1.59 | 5.30 | 1.97E-09 | 5.18E-08 |
| 2 h | ENSRNOG00000013202 | Akap7 | -1.02 | 6.09 | 2.16E-09 | 5.62E-08 |
| 2 h | ENSRNOG00000004720 | Kcnj2 | 1.54 | 2.76 | 2.18E-09 | 5.64E-08 |
| 2 h | ENSRNOG00000021128 | Kcnj11 | -1.12 | 2.67 | 2.54E-09 | 6.54E-08 |
| 2 h | ENSRNOG00000019202 | PVR | 1.43 | 1.24 | 2.79E-09 | 7.14E-08 |
| 2 h | ENSRNOG00000029678 | D3ZUE7_RAT | -1.16 | 2.13 | 2.83E-09 | 7.24E-08 |
| 2 h | ENSRNOG00000013621 | Rnd1 | 1.34 | 3.59 | 3.32E-09 | 8.43E-08 |
| 2 h | ENSRNOG00000013231 | Ptafr | 1.03 | 2.89 | 3.54E-09 | 8.96E-08 |
| 2 h | ENSRNOG00000012193 | Cited2 | 1.17 | 5.73 | 3.72E-09 | 9.35E-08 |
| 2 h | ENSRNOG00000021517 | Tmem231 | -1.16 | 2.52 | 4.68E-09 | 1.16E-07 |
| 2 h | ENSRNOG00000016551 | Bcl2l11 | 1.58 | 0.70 | 5.47E-09 | 1.35E-07 |
| 2 h | ENSRNOG00000002515 | ATP7A_RAT | 1.02 | 4.00 | 8.93E-09 | 2.16E-07 |
| 2 h | ENSRNOG00000025114 | Cebpb | 1.69 | 4.97 | 1.17E-08 | 2.79E-07 |
| 2 h | ENSRNOG00000018254 | Sncaip | 1.37 | 2.88 | 1.26E-08 | 2.99E-07 |
| 2 h | ENSRNOG00000001793 | F1M3W7_RAT | 1.11 | 6.47 | 1.28E-08 | 3.03E-07 |
| 2 h | ENSRNOG00000034140 | Htra4 | 3.30 | 4.57 | 1.34E-08 | 3.16E-07 |
| 2 h | ENSRNOG00000016728 | LOC100362710 | -1.48 | 5.58 | 1.70E-08 | 3.97E-07 |
| 2 h | ENSRNOG00000007822 | Vgll4 | 1.20 | 4.89 | 1.83E-08 | 4.22E-07 |
| 2 h | ENSRNOG00000020559 | E9PTV7_RAT | 1.54 | 2.96 | 2.16E-08 | 4.95E-07 |
| 2 h | ENSRNOG00000000471 | B3galt4 | -1.32 | 1.49 | 3.49E-08 | 7.70E-07 |
| 2 h | ENSRNOG00000008026 | Fam148c | -1.16 | 2.53 | 3.94E-08 | 8.63E-07 |
| 2 h | ENSRNOG00000012280 | Ptx3 | 1.80 | 1.59 | 4.59E-08 | 9.96E-07 |
| 2 h | ENSRNOG00000043366 | ENSRNOG00000043366 | -1.43 | 3.53 | 7.62E-08 | 1.60E-06 |
| 2 h | ENSRNOG00000013436 | Pde7b | 1.37 | 5.17 | 8.00E-08 | 1.68E-06 |
| 2 h | ENSRNOG00000025298 | Dusp9 | 1.17 | 1.59 | 9.80E-08 | 2.02E-06 |
| 2 h | ENSRNOG00000038540 | Gp5 | 1.22 | 1.86 | 1.02E-07 | 2.10E-06 |
| 2 h | ENSRNOG00000007707 | Znf467 | -1.10 | 4.00 | 1.18E-07 | 2.40E-06 |
| 2 h | ENSRNOG00000042785 | RGD1566319 | 1.44 | 1.26 | 1.48E-07 | 2.98E-06 |
| 2 h | ENSRNOG00000037299 | LOC100359829 | -1.37 | 1.75 | 2.30E-07 | 4.44E-06 |
| 2 h | ENSRNOG00000005386 | Kitlg | 1.59 | 6.14 | 2.54E-07 | 4.87E-06 |
| 2 h | ENSRNOG00000017539 | Mmp9 | 2.07 | 1.89 | 2.66E-07 | 5.06E-06 |
| 2 h | ENSRNOG00000016500 | RGD1563891 | -1.79 | 2.85 | 3.50E-07 | 6.52E-06 |
| 2 h | ENSRNOG00000042874 | F1M1K7_RAT | 1.82 | 0.26 | 4.09E-07 | 7.53E-06 |
| 2 h | ENSRNOG00000007300 | C1qtnf6 | -1.90 | 1.16 | 4.96E-07 | 9.01E-06 |
| 2 h | ENSRNOG00000014259 | Mycl1 | -1.15 | 4.40 | 6.45E-07 | 1.15E-05 |
| 2 h | ENSRNOG00000009157 | Fut4 | -1.84 | 0.42 | 9.28E-07 | 1.61E-05 |
| 2 h | ENSRNOG00000022326 | Ccdc142 | -1.17 | 2.41 | 1.05E-06 | 1.80E-05 |
| 2 h | ENSRNOG00000009012 | Asb2 | -1.28 | 1.29 | 1.19E-06 | 2.03E-05 |
| 2 h | ENSRNOG00000004125 | C4bpb | -2.45 | -0.21 | 1.44E-06 | 2.41E-05 |
| 2 h | ENSRNOG00000035576 | rno-mir-27b | 1.47 | 0.06 | 1.88E-06 | 3.06E-05 |
| 2 h | ENSRNOG00000002930 | Ppl | -1.65 | 4.64 | 1.98E-06 | 3.20E-05 |
| 2 h | ENSRNOG00000020552 | Fosl1 | 3.59 | -0.83 | 2.54E-06 | 4.01E-05 |
| 2 h | ENSRNOG00000038365 | F1LXA9_RAT | 1.76 | 3.80 | 2.73E-06 | 4.26E-05 |
| 2 h | ENSRNOG00000010211 | LOC100362431 | -1.20 | 1.50 | 2.76E-06 | 4.31E-05 |
| 2 h | ENSRNOG00000023473 | RGD1561238 | 1.99 | 0.68 | 3.14E-06 | 4.84E-05 |
| 2 h | ENSRNOG00000009407 | Galr2 | -2.11 | 0.16 | 4.88E-06 | 7.23E-05 |
| 2 h | ENSRNOG00000024119 | Ghsr | 1.73 | 2.42 | 5.62E-06 | 8.27E-05 |
| 2 h | ENSRNOG00000028190 | D4ACF8_RAT | -1.61 | 1.22 | 6.06E-06 | 8.85E-05 |
| 2 h | ENSRNOG00000008430 | Spry3 | 1.05 | 1.55 | 6.28E-06 | 9.16E-05 |
| 2 h | ENSRNOG00000045465 | SNORD113 | 1.13 | 1.10 | 7.11E-06 | 0.000102879 |
| 2 h | ENSRNOG00000006588 | Meox2 | -1.66 | 0.20 | 7.14E-06 | 0.000103255 |
| 2 h | ENSRNOG00000010047 | LOC100363484 | -1.95 | 0.92 | 7.50E-06 | 0.000107925 |
| 2 h | ENSRNOG00000005781 | Wnt16 | -1.23 | 1.05 | 1.02E-05 | 0.000142241 |
| 2 h | ENSRNOG00000013944 | Zc3hav1l | -1.85 | -0.10 | 1.20E-05 | 0.000165144 |
| 2 h | ENSRNOG00000025184 | Prss35 | 1.07 | 4.03 | 1.28E-05 | 0.00017579 |
| 2 h | ENSRNOG00000023257 | Adamts9 | 1.02 | 4.58 | 1.52E-05 | 0.000205161 |
| 2 h | ENSRNOG00000010158 | Magel2 | 1.28 | 1.08 | 1.60E-05 | 0.000214459 |
| 2 h | ENSRNOG00000006396 | Sdc1 | 1.69 | 2.32 | 1.61E-05 | 0.000215569 |
| 2 h | ENSRNOG00000004641 | Sstr4 | 1.03 | 3.41 | 1.75E-05 | 0.000232628 |
| 2 h | ENSRNOG00000034363 | SNORD113 | 1.16 | 1.58 | 1.78E-05 | 0.000236707 |
| 2 h | ENSRNOG00000002408 | Rbm47 | 1.61 | 0.02 | 1.94E-05 | 0.000254921 |
| 2 h | ENSRNOG00000017093 | RGD1311307 | -1.01 | 3.59 | 2.47E-05 | 0.00031797 |
| 2 h | ENSRNOG00000005410 | Cdca7l | -1.13 | 1.06 | 2.72E-05 | 0.000345811 |
| 2 h | ENSRNOG00000042891 | D3ZB15_RAT | -1.64 | 0.41 | 4.36E-05 | 0.000529707 |
| 2 h | ENSRNOG00000042541 | Cpg1 | 5.48 | -0.70 | 4.92E-05 | 0.000587671 |
| 2 h | ENSRNOG00000027445 | D4A242_RAT | 1.03 | 1.63 | 5.20E-05 | 0.000616867 |
| 2 h | ENSRNOG00000035152 | SNORD113 | 1.14 | 0.94 | 5.32E-05 | 0.000628542 |
| 2 h | ENSRNOG00000022244 | Olr1462 | -1.42 | 1.02 | 6.38E-05 | 0.000737667 |
| 2 h | ENSRNOG00000009211 | C3ar1 | 1.82 | -0.23 | 7.14E-05 | 0.000813018 |
| 2 h | ENSRNOG00000018299 | RGD1564677 | 1.29 | 0.17 | 7.39E-05 | 0.000839541 |
| 2 h | ENSRNOG00000039897 | F1M636_RAT | 1.48 | 0.20 | 7.65E-05 | 0.000865019 |
| 2 h | ENSRNOG00000032539 | Spag8 | -1.74 | 0.18 | 7.89E-05 | 0.000886932 |
| 2 h | ENSRNOG00000024952 | Atp8b1 | -1.62 | 0.57 | 8.94E-05 | 0.000990714 |
| 2 h | ENSRNOG00000014705 | Rbm20 | -1.61 | 1.51 | 9.20E-05 | 0.001015568 |
| 2 h | ENSRNOG00000034514 | SNORD113 | 1.79 | -0.55 | 9.73E-05 | 0.001064816 |
| 2 h | ENSRNOG00000007533 | Rab27a | -1.05 | 3.50 | 0.000113526 | 0.001215248 |
| 2 h | ENSRNOG00000002097 | Rasl11b | 1.34 | 3.20 | 0.000114414 | 0.001221953 |
| 2 h | ENSRNOG00000039969 | D3ZJR7_RAT | -1.80 | 0.18 | 0.000116023 | 0.001233502 |
| 2 h | ENSRNOG00000005535 | LOC100361923 | -1.05 | 1.48 | 0.000120563 | 0.001273081 |
| 2 h | ENSRNOG00000002475 | RGD1565798 | 1.38 | -0.10 | 0.000130095 | 0.001356373 |
| 2 h | ENSRNOG00000020208 | Gdf10 | -1.47 | 3.59 | 0.000132195 | 0.0013752 |
| 2 h | ENSRNOG00000034443 | SNORD113 | 1.19 | 1.04 | 0.000137518 | 0.001422134 |
| 2 h | ENSRNOG00000011101 | Twist1 | -1.09 | 0.98 | 0.000162819 | 0.001655703 |
| 2 h | ENSRNOG00000023423 | Aim1l | 1.23 | 0.10 | 0.000163185 | 0.001657019 |
| 2 h | ENSRNOG00000019184 | Npr3 | 1.10 | 1.35 | 0.000166218 | 0.001682942 |
| 2 h | ENSRNOG00000030299 | LOC683302 | 1.35 | 0.21 | 0.000166207 | 0.001682942 |
| 2 h | ENSRNOG00000018340 | Marveld2 | -1.33 | 1.63 | 0.000195874 | 0.001950811 |
| 2 h | ENSRNOG00000043122 | B0K035_RAT | -1.24 | 0.77 | 0.000226516 | 0.002221278 |
| 2 h | ENSRNOG00000035716 | SNORD113 | 1.93 | -0.19 | 0.000258844 | 0.002505016 |
| 2 h | ENSRNOG00000034534 | SNORD113 | 1.18 | 0.54 | 0.000261787 | 0.002526522 |
| 2 h | ENSRNOG00000017887 | Mutyh | -1.01 | 1.23 | 0.000277762 | 0.002656928 |
| 2 h | ENSRNOG00000039861 | LOC690617 | -1.55 | 0.08 | 0.000340742 | 0.003156029 |
| 2 h | ENSRNOG00000018121 | E9PTW0_RAT | 1.23 | 0.03 | 0.000362897 | 0.003328865 |
| 2 h | ENSRNOG00000035568 | rno-mir-24-1 | 1.45 | -0.32 | 0.000378777 | 0.003444608 |
| 2 h | ENSRNOG00000009870 | Tmem88 | 1.14 | 0.74 | 0.000429655 | 0.003842511 |
| 2 h | ENSRNOG00000019270 | P2ry6 | 1.13 | 0.87 | 0.00046073 | 0.004089128 |
| 2 h | ENSRNOG00000034376 | U5 | 1.50 | -0.73 | 0.000490385 | 0.004327666 |
| 2 h | ENSRNOG00000013057 | Prc1 | 1.07 | 1.65 | 0.000575024 | 0.004962189 |
| 2 h | ENSRNOG00000020864 | LOC100359836 | -1.02 | 0.73 | 0.000616216 | 0.005252235 |
| 2 h | ENSRNOG00000036927 | F1M8X4_RAT | -1.77 | -0.17 | 0.000623402 | 0.005294955 |
| 2 h | ENSRNOG00000008125 | Igbp1b | -1.76 | -0.33 | 0.000643151 | 0.005429803 |
| 2 h | ENSRNOG00000004744 | Fam84b | -1.20 | 1.44 | 0.000645643 | 0.005447567 |
| 2 h | ENSRNOG00000018108 | Irf4 | -1.19 | 0.42 | 0.000789813 | 0.006465585 |
| 2 h | ENSRNOG00000023465 | LOC500300 | 1.51 | 1.10 | 0.000797507 | 0.006513359 |
| 2 h | ENSRNOG00000028662 | LOC100363455 | -1.28 | 0.17 | 0.000906992 | 0.007243027 |
| 2 h | ENSRNOG00000026091 | Slc10a4 | 1.41 | -0.23 | 0.000948302 | 0.007508778 |
| 2 h | ENSRNOG00000023968 | F1M0R6_RAT | -1.23 | 0.27 | 0.000972533 | 0.007670319 |
| 2 h | ENSRNOG00000011901 | Rrad | 1.39 | 0.60 | 0.001027517 | 0.007996044 |
| 2 h | ENSRNOG00000027784 | Tsku | -1.68 | -0.28 | 0.00115108 | 0.008739247 |
| 2 h | ENSRNOG00000034805 | SNORD113 | 1.55 | -0.62 | 0.0011928 | 0.008971344 |
| 2 h | ENSRNOG00000010516 | Plau | -1.04 | 1.64 | 0.001296288 | 0.00958242 |
| 2 h | ENSRNOG00000003927 | Cd55 | -1.02 | 2.51 | 0.001440789 | 0.010425279 |
| 2 h | ENSRNOG00000021266 | Prokr2 | 2.09 | 0.22 | 0.001462104 | 0.010563159 |
| 2 h | ENSRNOG00000029292 | LOC500877 | -1.43 | 0.39 | 0.001566357 | 0.011153727 |
| 2 h | ENSRNOG00000008412 | Gprc5a | 1.55 | -0.89 | 0.001693084 | 0.011942714 |
| 2 h | ENSRNOG00000029812 | Mex3a | 1.43 | 0.50 | 0.00172373 | 0.012128385 |
| 2 h | ENSRNOG00000021434 | RGD1562211 | -1.36 | 0.06 | 0.001744121 | 0.01223502 |
| 2 h | ENSRNOG00000002549 | Htr5b | -1.83 | 1.55 | 0.001766049 | 0.012345618 |
| 2 h | ENSRNOG00000016917 | CLCN1_RAT | -1.56 | -0.59 | 0.001945414 | 0.013408005 |
| 2 h | ENSRNOG00000026036 | Pdyn | 1.19 | 7.77 | 0.002289101 | 0.015378127 |
| 2 h | ENSRNOG00000004440 | Fignl1 | -1.20 | -0.04 | 0.002383677 | 0.01590957 |
| 2 h | ENSRNOG00000028016 | Ncf2 | 1.17 | 0.21 | 0.002558601 | 0.016900262 |
| 2 h | ENSRNOG00000032946 | LOC681677 | -1.37 | 0.44 | 0.002573662 | 0.016943878 |
| 2 h | ENSRNOG00000036700 | Fscn2 | -1.08 | 0.15 | 0.002619441 | 0.017194918 |
| 2 h | ENSRNOG00000019719 | Kcna5 | -1.09 | 0.71 | 0.002851257 | 0.018374623 |
| 2 h | ENSRNOG00000034543 | SNORD113 | 1.07 | -0.30 | 0.00298352 | 0.019069236 |
| 2 h | ENSRNOG00000009180 | Xkr7 | 1.08 | 2.78 | 0.003147723 | 0.019928 |
| 2 h | ENSRNOG00000042665 | F1LTD4_RAT | -1.01 | 0.25 | 0.003256094 | 0.020513446 |
| 2 h | ENSRNOG00000019783 | Akap3 | -1.04 | 0.11 | 0.003288792 | 0.020644016 |
| 2 h | ENSRNOG00000015541 | Gnb3 | -1.65 | -0.43 | 0.0033245 | 0.020812262 |
| 2 h | ENSRNOG00000000439 | Ager | -1.15 | 0.89 | 0.003450293 | 0.021484663 |
| 2 h | ENSRNOG00000011923 | Osap | -1.03 | 0.17 | 0.003630356 | 0.022347233 |
| 2 h | ENSRNOG00000011332 | Clspn | -1.27 | 1.01 | 0.003669106 | 0.022527093 |
| 2 h | ENSRNOG00000006549 | LOC100362205 | 1.18 | -0.49 | 0.003676809 | 0.022564506 |
| 2 h | ENSRNOG00000015247 | F1M1D4_RAT | -1.06 | 0.06 | 0.004579529 | 0.026775903 |
| 2 h | ENSRNOG00000007301 | Gdf9 | -1.12 | 0.12 | 0.004613049 | 0.02690881 |
| 2 h | ENSRNOG00000038916 | D3ZX91_RAT | 1.01 | 0.03 | 0.004862738 | 0.028002228 |
| 2 h | ENSRNOG00000045495 | U6 | -1.34 | -0.38 | 0.00488939 | 0.028099966 |
| 2 h | ENSRNOG00000016584 | Mamdc4 | -1.58 | -0.51 | 0.005209768 | 0.029734055 |
| 2 h | ENSRNOG00000010111 | RGD1307749 | 1.03 | 0.22 | 0.005223884 | 0.029790372 |
| 2 h | ENSRNOG00000031452 | Otop3 | 1.30 | -0.50 | 0.006790831 | 0.036909566 |
| 2 h | ENSRNOG00000021627 | Hmox3 | 1.00 | 0.24 | 0.006957322 | 0.037590481 |
| 2 h | ENSRNOG00000021568 | Ccdc110 | 1.23 | -0.36 | 0.007399291 | 0.03960315 |
| 2 h | ENSRNOG00000031478 | LOC100362089 | -1.10 | -0.19 | 0.007743333 | 0.041115018 |
| 2 h | ENSRNOG00000007754 | Insm2 | 1.56 | -0.67 | 0.007822128 | 0.041407966 |
| 2 h | ENSRNOG00000037467 | Ffar3 | 2.56 | -0.89 | 0.008216429 | 0.043072518 |
| 2 h | ENSRNOG00000040052 | RT1-M6-2 | 1.06 | 3.83 | 0.008874519 | 0.045769541 |
| 2 h | ENSRNOG00000002439 | Inhbb | 1.66 | 0.31 | 0.008992364 | 0.046275199 |
| 2 h | ENSRNOG00000003650 | LOC100360582 | -1.01 | 0.24 | 0.009498084 | 0.048367938 |
| 2 h | ENSRNOG00000022335 | Rad21l1 | -1.19 | 0.00 | 0.00957017 | 0.04862409 |
| 5 h | ENSRNOG00000005561 | Dbc1 | 2.72 | 8.50 | 0 | 0 |
| 5 h | ENSRNOG00000006184 | Pmepa1 | 3.79 | 5.76 | 5.71E-244 | 4.01E-240 |
| 5 h | ENSRNOG00000013945 | Itpkc | 3.23 | 4.66 | 5.52E-220 | 2.58E-216 |
| 5 h | ENSRNOG00000015401 | Mapk4 | 3.44 | 5.77 | 5.29E-213 | 1.86E-209 |
| 5 h | ENSRNOG00000007346 | Grasp | 3.90 | 6.62 | 1.15E-212 | 3.23E-209 |
| 5 h | ENSRNOG00000011387 | Tet3 | 2.60 | 6.63 | 4.98E-171 | 1.16E-167 |
| 5 h | ENSRNOG00000018484 | Plk3 | 5.49 | 3.77 | 5.24E-170 | 1.05E-166 |
| 5 h | ENSRNOG00000001006 | Nptx2 | 4.68 | 7.11 | 1.10E-168 | 1.92E-165 |
| 5 h | ENSRNOG00000043465 | Arc | 7.24 | 9.91 | 1.58E-166 | 2.46E-163 |
| 5 h | ENSRNOG00000016702 | Gsg1l | 4.05 | 3.63 | 5.19E-158 | 7.28E-155 |
| 5 h | ENSRNOG00000000130 | Dnajb5 | 2.99 | 7.97 | 1.05E-156 | 1.34E-153 |
| 5 h | ENSRNOG00000000529 | Pim1 | 3.44 | 4.26 | 3.42E-152 | 3.99E-149 |
| 5 h | ENSRNOG00000004100 | Trib1 | 4.84 | 4.60 | 3.91E-151 | 4.21E-148 |
| 5 h | ENSRNOG00000022561 | D3ZV58_RAT | 2.64 | 5.79 | 5.59E-150 | 5.60E-147 |
| 5 h | ENSRNOG00000014426 | Lox | 5.62 | 2.34 | 5.49E-146 | 5.13E-143 |
| 5 h | ENSRNOG00000033433 | Csrnp1 | 3.27 | 4.94 | 9.21E-146 | 8.07E-143 |
| 5 h | ENSRNOG00000006118 | Klf10 | 3.90 | 6.56 | 8.19E-142 | 6.75E-139 |
| 5 h | ENSRNOG00000018359 | Smad7 | 3.20 | 5.77 | 1.03E-128 | 8.02E-126 |
| 5 h | ENSRNOG00000011824 | Trh | 8.75 | 5.62 | 1.59E-125 | 1.17E-122 |
| 5 h | ENSRNOG00000037085 | Xirp1 | 9.79 | 3.21 | 3.99E-125 | 2.79E-122 |
| 5 h | ENSRNOG00000019822 | Gadd45b | 3.67 | 6.55 | 3.04E-123 | 2.03E-120 |
| 5 h | ENSRNOG00000017828 | Egr3 | 3.87 | 8.00 | 5.07E-120 | 3.23E-117 |
| 5 h | ENSRNOG00000002129 | Lrrc8b | 1.91 | 7.66 | 1.95E-116 | 1.19E-113 |
| 5 h | ENSRNOG00000019533 | Klhl3 | 3.29 | 4.79 | 2.99E-111 | 1.74E-108 |
| 5 h | ENSRNOG00000006828 | F1M4U9_RAT | 4.29 | 4.42 | 1.60E-107 | 8.96E-105 |
| 5 h | ENSRNOG00000012934 | Arhgef7 | 1.89 | 7.38 | 7.27E-105 | 3.92E-102 |
| 5 h | ENSRNOG00000020770 | Arl4d | 3.37 | 5.67 | 8.16E-105 | 4.24E-102 |
| 5 h | ENSRNOG00000020937 | Ubtf | 1.96 | 7.60 | 1.44E-103 | 7.21E-101 |
| 5 h | ENSRNOG00000009899 | D3ZWL1_RAT | 2.02 | 7.48 | 2.29E-102 | 1.11E-99 |
| 5 h | ENSRNOG00000014320 | Inhba | 5.40 | 5.21 | 7.98E-102 | 3.73E-99 |
| 5 h | ENSRNOG00000001416 | Vgf | 3.70 | 6.77 | 2.68E-101 | 1.21E-98 |
| 5 h | ENSRNOG00000019229 | Fbl | 3.02 | 5.29 | 3.55E-100 | 1.56E-97 |
| 5 h | ENSRNOG00000009972 | Rara | 3.66 | 4.39 | 1.06E-99 | 4.51E-97 |
| 5 h | ENSRNOG00000043168 | Homer1 | 2.47 | 6.70 | 6.61E-98 | 2.72E-95 |
| 5 h | ENSRNOG00000037613 | Kdm6b | 3.85 | 5.36 | 1.65E-97 | 6.62E-95 |
| 5 h | ENSRNOG00000028832 | Sorcs3 | 2.83 | 6.51 | 1.86E-97 | 7.24E-95 |
| 5 h | ENSRNOG00000023013 | Mar-03 | 3.17 | 3.39 | 2.36E-96 | 8.95E-94 |
| 5 h | ENSRNOG00000005140 | Prmt8 | 1.70 | 6.03 | 2.30E-95 | 8.50E-93 |
| 5 h | ENSRNOG00000017193 | Lingo1 | 3.24 | 7.37 | 5.68E-95 | 2.04E-92 |
| 5 h | ENSRNOG00000016326 | Cx3cl1 | 2.11 | 8.97 | 4.87E-93 | 1.71E-90 |
| 5 h | ENSRNOG00000002525 | Ptgs2 | 5.24 | 9.04 | 1.06E-92 | 3.61E-90 |
| 5 h | ENSRNOG00000020433 | Actn4 | 1.41 | 7.48 | 2.10E-92 | 7.03E-90 |
| 5 h | ENSRNOG00000015691 | RGD1306526 | 1.57 | 8.05 | 3.77E-91 | 1.23E-88 |
| 5 h | ENSRNOG00000006816 | Ubr5 | 1.17 | 8.88 | 1.27E-90 | 4.04E-88 |
| 5 h | ENSRNOG00000010060 | Panx1 | 2.25 | 4.79 | 4.42E-86 | 1.38E-83 |
| 5 h | ENSRNOG00000030091 | LOC681749 | 2.97 | 5.33 | 2.42E-84 | 7.39E-82 |
| 5 h | ENSRNOG00000016180 | Pdp1 | 2.18 | 6.75 | 1.99E-83 | 5.95E-81 |
| 5 h | ENSRNOG00000020867 | Numbl | 2.09 | 6.29 | 6.01E-83 | 1.76E-80 |
| 5 h | ENSRNOG00000007797 | Rbpsuh | 2.55 | 4.96 | 6.62E-83 | 1.89E-80 |
| 5 h | ENSRNOG00000010058 | Spry2 | 2.97 | 5.96 | 8.73E-83 | 2.45E-80 |
| 5 h | ENSRNOG00000008415 | Nab2 | 2.26 | 5.88 | 5.50E-82 | 1.51E-79 |
| 5 h | ENSRNOG00000010489 | SMAG1_RAT | 3.44 | 5.35 | 6.82E-82 | 1.84E-79 |
| 5 h | ENSRNOG00000015913 | Tspan5 | 1.76 | 6.86 | 3.50E-81 | 9.26E-79 |
| 5 h | ENSRNOG00000004110 | Trib2 | 3.31 | 5.37 | 2.25E-80 | 5.84E-78 |
| 5 h | ENSRNOG00000002657 | Pla2g4a | 2.71 | 3.87 | 4.60E-80 | 1.17E-77 |
| 5 h | ENSRNOG00000004697 | BAALC_RAT | 2.03 | 7.46 | 1.84E-78 | 4.60E-76 |
| 5 h | ENSRNOG00000003990 | Grb2 | 1.23 | 8.09 | 2.24E-78 | 5.51E-76 |
| 5 h | ENSRNOG00000004307 | Tor3a | 2.13 | 3.43 | 6.01E-76 | 1.45E-73 |
| 5 h | ENSRNOG00000017087 | Man1c1 | 2.37 | 5.50 | 2.27E-75 | 5.40E-73 |
| 5 h | ENSRNOG00000004726 | F1M244_RAT | 1.78 | 5.38 | 1.55E-74 | 3.63E-72 |
| 5 h | ENSRNOG00000013196 | RGD1562846 | 3.98 | 2.56 | 2.54E-73 | 5.84E-71 |
| 5 h | ENSRNOG00000000596 | Fyn | 1.45 | 7.74 | 2.77E-73 | 6.26E-71 |
| 5 h | ENSRNOG00000037615 | ENSRNOG00000037615 | 3.87 | 2.85 | 1.86E-72 | 4.14E-70 |
| 5 h | ENSRNOG00000004309 | Actn1 | 1.69 | 6.60 | 8.69E-72 | 1.90E-69 |
| 5 h | ENSRNOG00000011421 | Smap2 | 1.15 | 7.63 | 2.26E-70 | 4.87E-68 |
| 5 h | ENSRNOG00000010165 | D3ZYQ3_RAT | 2.58 | 2.00 | 1.26E-69 | 2.63E-67 |
| 5 h | ENSRNOG00000011463 | Psmb2 | 1.58 | 6.39 | 3.81E-69 | 7.85E-67 |
| 5 h | ENSRNOG00000023534 | Haus8 | 4.92 | 3.76 | 3.95E-69 | 8.02E-67 |
| 5 h | ENSRNOG00000031167 | Srxn1 | 2.36 | 4.94 | 1.21E-68 | 2.43E-66 |
| 5 h | ENSRNOG00000009007 | LOC100363621 | 1.76 | 4.97 | 1.77E-68 | 3.50E-66 |
| 5 h | ENSRNOG00000020225 | Dlgap4 | 1.42 | 7.22 | 1.41E-67 | 2.74E-65 |
| 5 h | ENSRNOG00000007031 | Trim9 | 2.01 | 8.20 | 1.53E-67 | 2.95E-65 |
| 5 h | ENSRNOG00000010208 | Timp1 | 5.73 | 3.61 | 2.27E-67 | 4.31E-65 |
| 5 h | ENSRNOG00000014061 | Dusp5 | 4.47 | 4.30 | 3.01E-67 | 5.63E-65 |
| 5 h | ENSRNOG00000017116 | Znf532 | 1.71 | 6.88 | 4.00E-67 | 7.38E-65 |
| 5 h | ENSRNOG00000020584 | Efna3 | 1.79 | 4.31 | 1.65E-66 | 3.00E-64 |
| 5 h | ENSRNOG00000043123 | Rnf128 | 2.11 | 5.43 | 2.06E-66 | 3.70E-64 |
| 5 h | ENSRNOG00000006396 | Sdc1 | 6.55 | 2.32 | 3.57E-66 | 6.33E-64 |
| 5 h | ENSRNOG00000020905 | Hdac5 | 1.66 | 6.05 | 1.68E-65 | 2.95E-63 |
| 5 h | ENSRNOG00000002453 | Taf9b | 1.33 | 6.59 | 3.87E-65 | 6.69E-63 |
| 5 h | ENSRNOG00000011352 | Furin | 1.79 | 6.13 | 4.18E-65 | 7.14E-63 |
| 5 h | ENSRNOG00000004518 | Cacnb1 | 1.23 | 7.29 | 8.41E-64 | 1.42E-61 |
| 5 h | ENSRNOG00000024363 | Sertad1 | 2.38 | 4.53 | 3.46E-63 | 5.77E-61 |
| 5 h | ENSRNOG00000000062 | Pcgf3 | 1.52 | 5.77 | 4.77E-63 | 7.86E-61 |
| 5 h | ENSRNOG00000008919 | Arpp21 | 2.94 | 8.42 | 1.70E-62 | 2.77E-60 |
| 5 h | ENSRNOG00000007925 | Pak6 | 1.93 | 6.44 | 4.28E-61 | 6.89E-59 |
| 5 h | ENSRNOG00000019861 | Tollip | 1.07 | 7.42 | 1.07E-60 | 1.70E-58 |
| 5 h | ENSRNOG00000001704 | Runx1 | 4.66 | 1.91 | 1.18E-59 | 1.84E-57 |
| 5 h | ENSRNOG00000035476 | rno-mir-212 | 8.02 | 1.34 | 1.18E-59 | 1.84E-57 |
| 5 h | ENSRNOG00000010888 | Ankrd33b | 1.92 | 5.54 | 2.45E-59 | 3.77E-57 |
| 5 h | ENSRNOG00000016734 | Emilin3 | 7.81 | -0.43 | 1.27E-58 | 1.94E-56 |
| 5 h | ENSRNOG00000027491 | Vldlr | 1.50 | 6.36 | 1.82E-58 | 2.74E-56 |
| 5 h | ENSRNOG00000032569 | Lingo3 | 2.07 | 4.37 | 2.14E-58 | 3.19E-56 |
| 5 h | ENSRNOG00000011631 | Fst | 5.30 | 0.40 | 8.08E-58 | 1.19E-55 |
| 5 h | ENSRNOG00000001070 | Cdk2ap1 | 1.33 | 5.61 | 3.45E-56 | 5.04E-54 |
| 5 h | ENSRNOG00000020298 | Bag3 | 3.64 | 5.32 | 2.05E-55 | 2.97E-53 |
| 5 h | ENSRNOG00000008752 | Nrf1 | 1.45 | 4.44 | 2.53E-55 | 3.62E-53 |
| 5 h | ENSRNOG00000034240 | D4A1S3_RAT | 2.30 | 5.15 | 2.70E-55 | 3.83E-53 |
| 5 h | ENSRNOG00000004888 | Spred2 | 2.35 | 4.35 | 2.76E-55 | 3.87E-53 |
| 5 h | ENSRNOG00000023509 | Irs2 | 1.74 | 7.01 | 8.50E-55 | 1.18E-52 |
| 5 h | ENSRNOG00000017037 | Otud3 | 1.90 | 3.99 | 8.94E-55 | 1.23E-52 |
| 5 h | ENSRNOG00000006832 | Zdhhc5 | 1.17 | 6.22 | 1.12E-54 | 1.53E-52 |
| 5 h | ENSRNOG00000014429 | Arfgap2 | 1.07 | 6.04 | 1.17E-54 | 1.57E-52 |
| 5 h | ENSRNOG00000000640 | Egr2 | 4.47 | 5.24 | 4.54E-54 | 6.06E-52 |
| 5 h | ENSRNOG00000000956 | Rasl11a | 3.18 | 3.03 | 2.48E-53 | 3.28E-51 |
| 5 h | ENSRNOG00000015415 | Rhoq | 2.08 | 5.05 | 7.50E-53 | 9.83E-51 |
| 5 h | ENSRNOG00000030034 | Sox11 | 3.77 | 4.89 | 3.08E-52 | 4.00E-50 |
| 5 h | ENSRNOG00000021513 | Rtn4rl2 | 2.82 | 5.24 | 3.41E-52 | 4.39E-50 |
| 5 h | ENSRNOG00000008553 | Mthfr | 2.57 | 2.71 | 3.68E-52 | 4.69E-50 |
| 5 h | ENSRNOG00000015434 | Midn | 2.47 | 3.97 | 1.16E-51 | 1.47E-49 |
| 5 h | ENSRNOG00000004823 | F1LUN1_RAT | 1.22 | 8.76 | 1.19E-51 | 1.48E-49 |
| 5 h | ENSRNOG00000001582 | Bach1 | 1.31 | 4.23 | 1.83E-50 | 2.25E-48 |
| 5 h | ENSRNOG00000010041 | Ube2g1 | 1.08 | 6.40 | 2.65E-50 | 3.23E-48 |
| 5 h | ENSRNOG00000014647 | Cbfb | 2.04 | 5.41 | 4.24E-50 | 5.12E-48 |
| 5 h | ENSRNOG00000026649 | Dnmt3a | 1.64 | 6.06 | 1.17E-49 | 1.41E-47 |
| 5 h | ENSRNOG00000021009 | Gapdhs | 5.79 | 0.64 | 1.58E-49 | 1.88E-47 |
| 5 h | ENSRNOG00000009525 | Rnf114 | 1.16 | 5.64 | 1.91E-49 | 2.25E-47 |
| 5 h | ENSRNOG00000007478 | Cry2 | 1.33 | 7.31 | 2.01E-49 | 2.35E-47 |
| 5 h | ENSRNOG00000032178 | Cenpa | 4.98 | 0.03 | 2.63E-49 | 3.04E-47 |
| 5 h | ENSRNOG00000010534 | F1LWB9_RAT | 1.75 | 3.32 | 7.65E-49 | 8.79E-47 |
| 5 h | ENSRNOG00000018804 | Fam65b | 1.97 | 6.67 | 9.43E-49 | 1.08E-46 |
| 5 h | ENSRNOG00000010488 | Zmiz1 | 2.42 | 6.63 | 1.10E-48 | 1.24E-46 |
| 5 h | ENSRNOG00000001141 | Srrm4 | 1.66 | 3.57 | 2.37E-48 | 2.66E-46 |
| 5 h | ENSRNOG00000025463 | LOC100125362 | 1.77 | 7.90 | 3.37E-48 | 3.75E-46 |
| 5 h | ENSRNOG00000004890 | Adcy8 | 3.19 | 4.25 | 4.49E-48 | 4.96E-46 |
| 5 h | ENSRNOG00000013930 | RNF4_RAT | 1.01 | 6.57 | 9.00E-48 | 9.86E-46 |
| 5 h | ENSRNOG00000019181 | Synpo | 2.12 | 7.12 | 1.18E-47 | 1.28E-45 |
| 5 h | ENSRNOG00000005353 | Rybp | 1.80 | 5.16 | 3.21E-47 | 3.46E-45 |
| 5 h | ENSRNOG00000013339 | Gmeb2 | 1.45 | 4.23 | 8.22E-47 | 8.80E-45 |
| 5 h | ENSRNOG00000007892 | Tram1 | 1.11 | 6.97 | 4.05E-46 | 4.31E-44 |
| 5 h | ENSRNOG00000019645 | Osbp2 | 1.49 | 7.47 | 4.38E-46 | 4.62E-44 |
| 5 h | ENSRNOG00000012815 | Chst10 | 1.38 | 6.03 | 1.15E-45 | 1.18E-43 |
| 5 h | ENSRNOG00000026026 | Stam | 1.22 | 6.88 | 2.57E-45 | 2.63E-43 |
| 5 h | ENSRNOG00000030628 | Eif4a1 | 1.14 | 8.47 | 3.00E-45 | 3.05E-43 |
| 5 h | ENSRNOG00000008144 | Irf1 | 1.60 | 3.54 | 3.31E-45 | 3.34E-43 |
| 5 h | ENSRNOG00000005157 | Rbbp7 | 1.32 | 7.99 | 3.84E-45 | 3.84E-43 |
| 5 h | ENSRNOG00000013135 | Ptpn12 | 1.47 | 6.04 | 1.14E-44 | 1.14E-42 |
| 5 h | ENSRNOG00000020651 | Cars | 1.34 | 6.14 | 1.20E-44 | 1.19E-42 |
| 5 h | ENSRNOG00000012623 | Arf4 | 1.24 | 7.39 | 1.89E-44 | 1.84E-42 |
| 5 h | ENSRNOG00000015845 | Fam129b | 1.93 | 4.88 | 1.89E-44 | 1.84E-42 |
| 5 h | ENSRNOG00000008845 | Pdrg1 | 1.18 | 5.31 | 1.91E-44 | 1.85E-42 |
| 5 h | ENSRNOG00000001189 | Sik1 | 2.51 | 5.54 | 3.20E-44 | 3.05E-42 |
| 5 h | ENSRNOG00000003348 | Rasd1 | 2.34 | 4.96 | 3.18E-44 | 3.05E-42 |
| 5 h | ENSRNOG00000000777 | RT1-S3 | 3.04 | 3.73 | 5.48E-44 | 5.19E-42 |
| 5 h | ENSRNOG00000014528 | F1M4G7_RAT | 1.30 | 5.63 | 5.97E-44 | 5.62E-42 |
| 5 h | ENSRNOG00000009540 | Gpr3 | 3.92 | 0.94 | 6.61E-44 | 6.18E-42 |
| 5 h | ENSRNOG00000009253 | Igsf9b | 3.16 | 4.40 | 6.98E-44 | 6.48E-42 |
| 5 h | ENSRNOG00000016811 | Fosb | 2.94 | 5.06 | 8.49E-44 | 7.84E-42 |
| 5 h | ENSRNOG00000006916 | SARDH_RAT | 2.11 | 3.98 | 1.90E-43 | 1.74E-41 |
| 5 h | ENSRNOG00000004049 | Baiap2 | 1.54 | 8.28 | 5.61E-43 | 5.11E-41 |
| 5 h | ENSRNOG00000009186 | Stmn4 | 1.26 | 7.98 | 6.54E-43 | 5.91E-41 |
| 5 h | ENSRNOG00000015459 | Heatr3 | 1.24 | 4.59 | 1.16E-42 | 1.04E-40 |
| 5 h | ENSRNOG00000014082 | Sp7 | 5.68 | 0.12 | 1.37E-42 | 1.22E-40 |
| 5 h | ENSRNOG00000013067 | Phf21b | 3.11 | 2.49 | 5.38E-42 | 4.77E-40 |
| 5 h | ENSRNOG00000005393 | Sertad2 | 1.53 | 4.03 | 9.06E-42 | 7.99E-40 |
| 5 h | ENSRNOG00000020848 | Adck4 | 1.47 | 4.32 | 2.63E-41 | 2.31E-39 |
| 5 h | ENSRNOG00000009338 | Kras | 1.26 | 6.07 | 2.77E-41 | 2.41E-39 |
| 5 h | ENSRNOG00000043498 | Sik2 | 1.19 | 6.20 | 6.78E-41 | 5.87E-39 |
| 5 h | ENSRNOG00000011503 | RGD1565350 | 2.07 | 3.26 | 1.08E-40 | 9.25E-39 |
| 5 h | ENSRNOG00000032922 | Dclk1 | 1.91 | 9.12 | 1.95E-40 | 1.65E-38 |
| 5 h | ENSRNOG00000001971 | Bbx | 1.19 | 5.77 | 3.65E-40 | 3.08E-38 |
| 5 h | ENSRNOG00000014516 | Hs6st1 | 1.08 | 5.76 | 1.11E-39 | 9.29E-38 |
| 5 h | ENSRNOG00000018893 | Sfrp4 | 2.33 | 1.51 | 1.48E-39 | 1.23E-37 |
| 5 h | ENSRNOG00000018498 | F1MA03_RAT | 1.94 | 5.39 | 1.66E-39 | 1.37E-37 |
| 5 h | ENSRNOG00000013154 | Sfpq | 1.78 | 6.80 | 2.49E-39 | 2.04E-37 |
| 5 h | ENSRNOG00000005724 | Map3k7 | 1.32 | 6.48 | 6.63E-39 | 5.40E-37 |
| 5 h | ENSRNOG00000042307 | D3ZED1_RAT | 1.78 | 4.01 | 8.26E-39 | 6.70E-37 |
| 5 h | ENSRNOG00000030983 | B3galt5 | -1.23 | 5.44 | 1.18E-38 | 9.51E-37 |
| 5 h | ENSRNOG00000042541 | Cpg1 | 6.64 | -0.70 | 1.24E-38 | 9.97E-37 |
| 5 h | ENSRNOG00000019475 | Sh3bp5 | -1.61 | 6.57 | 1.26E-38 | 1.01E-36 |
| 5 h | ENSRNOG00000002336 | D3ZMK4_RAT | 1.67 | 7.32 | 1.98E-38 | 1.56E-36 |
| 5 h | ENSRNOG00000043301 | F1M727_RAT | 1.13 | 4.58 | 1.97E-38 | 1.56E-36 |
| 5 h | ENSRNOG00000011582 | Rab3d | -1.71 | 4.50 | 2.61E-38 | 2.04E-36 |
| 5 h | ENSRNOG00000027540 | Fam102b | 1.28 | 4.49 | 2.62E-38 | 2.04E-36 |
| 5 h | ENSRNOG00000013101 | PCDH8_RAT | 2.76 | 8.53 | 3.65E-38 | 2.83E-36 |
| 5 h | ENSRNOG00000020061 | LOC100365924 | 1.61 | 5.22 | 3.85E-38 | 2.96E-36 |
| 5 h | ENSRNOG00000008533 | Eif2c2 | 1.78 | 6.36 | 6.01E-38 | 4.61E-36 |
| 5 h | ENSRNOG00000011921 | Dusp4 | 2.26 | 5.32 | 8.49E-38 | 6.47E-36 |
| 5 h | ENSRNOG00000019598 | Vegfa | 2.03 | 6.04 | 1.02E-37 | 7.71E-36 |
| 5 h | ENSRNOG00000012258 | Rras2 | 1.24 | 5.65 | 1.76E-37 | 1.33E-35 |
| 5 h | ENSRNOG00000007324 | Plxna2 | 1.66 | 8.12 | 2.20E-37 | 1.65E-35 |
| 5 h | ENSRNOG00000005209 | Spred1 | 1.11 | 6.03 | 2.75E-37 | 2.05E-35 |
| 5 h | ENSRNOG00000001720 | D4ADT9_RAT | 2.15 | 3.31 | 5.11E-37 | 3.79E-35 |
| 5 h | ENSRNOG00000005569 | Phospho1 | 1.98 | 3.77 | 5.21E-37 | 3.85E-35 |
| 5 h | ENSRNOG00000008445 | D4A3W8_RAT | 2.09 | 2.11 | 8.18E-37 | 5.97E-35 |
| 5 h | ENSRNOG00000010812 | Osbpl6 | 1.13 | 6.39 | 9.57E-37 | 6.95E-35 |
| 5 h | ENSRNOG00000013805 | Tnip2 | 2.03 | 2.11 | 9.92E-37 | 7.17E-35 |
| 5 h | ENSRNOG00000024711 | Sdk2 | 1.95 | 5.96 | 1.52E-36 | 1.09E-34 |
| 5 h | ENSRNOG00000017635 | Inpp5a | 1.22 | 4.89 | 2.56E-36 | 1.83E-34 |
| 5 h | ENSRNOG00000011951 | Plk2 | 2.38 | 8.58 | 3.23E-36 | 2.30E-34 |
| 5 h | ENSRNOG00000018839 | Ntrk2 | 1.13 | 9.13 | 4.00E-36 | 2.83E-34 |
| 5 h | ENSRNOG00000018911 | Pfkfb3 | 1.40 | 5.69 | 1.50E-35 | 1.06E-33 |
| 5 h | ENSRNOG00000003121 | Rtn4rl1 | 1.73 | 8.06 | 1.65E-35 | 1.16E-33 |
| 5 h | ENSRNOG00000024019 | Ccdc6 | 1.07 | 5.32 | 1.94E-35 | 1.35E-33 |
| 5 h | ENSRNOG00000015051 | Golga7b | 1.74 | 3.46 | 3.49E-35 | 2.43E-33 |
| 5 h | ENSRNOG00000015631 | Ippk | 1.50 | 5.25 | 5.99E-35 | 4.14E-33 |
| 5 h | ENSRNOG00000036662 | Wdr45l | 1.08 | 5.66 | 1.40E-34 | 9.56E-33 |
| 5 h | ENSRNOG00000007152 | Bhlhe40 | 3.04 | 6.43 | 1.52E-34 | 1.04E-32 |
| 5 h | ENSRNOG00000033026 | Dclk3 | -2.89 | 3.28 | 3.64E-34 | 2.45E-32 |
| 5 h | ENSRNOG00000043113 | Fam43b | 3.41 | 2.83 | 4.16E-34 | 2.79E-32 |
| 5 h | ENSRNOG00000023551 | Sp6 | 5.80 | -0.90 | 2.07E-33 | 1.38E-31 |
| 5 h | ENSRNOG00000037360 | ENSRNOG00000037360 | 1.34 | 3.50 | 2.10E-33 | 1.40E-31 |
| 5 h | ENSRNOG00000022216 | Abr | 1.26 | 9.90 | 2.46E-33 | 1.62E-31 |
| 5 h | ENSRNOG00000034025 | D4A4T5_RAT | 1.23 | 7.59 | 2.63E-33 | 1.73E-31 |
| 5 h | ENSRNOG00000019723 | Rbm12 | 1.02 | 5.96 | 2.90E-33 | 1.89E-31 |
| 5 h | ENSRNOG00000001730 | Acap2 | 1.12 | 7.24 | 1.74E-32 | 1.12E-30 |
| 5 h | ENSRNOG00000002303 | Kcnj12 | 2.28 | 2.78 | 2.80E-32 | 1.80E-30 |
| 5 h | ENSRNOG00000015801 | Spns2 | 1.22 | 6.57 | 3.52E-32 | 2.26E-30 |
| 5 h | ENSRNOG00000001128 | Tesc | 1.10 | 6.37 | 3.93E-32 | 2.51E-30 |
| 5 h | ENSRNOG00000013916 | Nefm | 1.32 | 8.16 | 6.42E-32 | 4.04E-30 |
| 5 h | ENSRNOG00000020871 | D3ZB23_RAT | 1.62 | 4.79 | 1.43E-31 | 8.89E-30 |
| 5 h | ENSRNOG00000007128 | Nop56 | 1.07 | 6.78 | 1.62E-31 | 9.94E-30 |
| 5 h | ENSRNOG00000037753 | Slc10a2 | 4.86 | -0.36 | 4.23E-31 | 2.57E-29 |
| 5 h | ENSRNOG00000007560 | D3ZJE5_RAT | 1.05 | 6.38 | 5.79E-31 | 3.50E-29 |
| 5 h | ENSRNOG00000029450 | D3ZNF1_RAT | 1.09 | 7.69 | 1.75E-30 | 1.05E-28 |
| 5 h | ENSRNOG00000025679 | Stk40 | 1.22 | 4.72 | 2.23E-30 | 1.33E-28 |
| 5 h | ENSRNOG00000014117 | Hmox1 | 2.52 | 2.65 | 5.27E-30 | 3.13E-28 |
| 5 h | ENSRNOG00000000902 | Hsph1 | 1.04 | 8.83 | 7.45E-30 | 4.41E-28 |
| 5 h | ENSRNOG00000012989 | Serinc2 | 3.58 | 3.87 | 9.07E-30 | 5.35E-28 |
| 5 h | ENSRNOG00000042855 | Ahdc1 | 1.56 | 6.45 | 1.02E-29 | 5.99E-28 |
| 5 h | ENSRNOG00000015007 | RGD1565591 | 1.29 | 6.14 | 1.45E-29 | 8.45E-28 |
| 5 h | ENSRNOG00000000866 | Rbmx | 1.15 | 5.89 | 2.19E-29 | 1.27E-27 |
| 5 h | ENSRNOG00000039902 | LOC683626 | 1.74 | 4.90 | 2.21E-29 | 1.28E-27 |
| 5 h | ENSRNOG00000007329 | Frmd6 | 2.15 | 3.91 | 2.67E-29 | 1.54E-27 |
| 5 h | ENSRNOG00000006146 | D3ZQ78_RAT | 3.32 | 0.38 | 3.37E-29 | 1.92E-27 |
| 5 h | ENSRNOG00000003563 | Blmh | 1.43 | 5.95 | 6.30E-29 | 3.56E-27 |
| 5 h | ENSRNOG00000042546 | RGD1562551 | 1.54 | 2.51 | 6.97E-29 | 3.92E-27 |
| 5 h | ENSRNOG00000004367 | Elk3 | 2.43 | 3.83 | 1.16E-28 | 6.52E-27 |
| 5 h | ENSRNOG00000011141 | Serpina11 | 3.55 | 0.38 | 2.43E-28 | 1.35E-26 |
| 5 h | ENSRNOG00000003813 | Kcnk10 | 2.57 | 4.15 | 4.64E-28 | 2.56E-26 |
| 5 h | ENSRNOG00000004099 | R3hdm1 | 1.23 | 6.60 | 6.88E-28 | 3.77E-26 |
| 5 h | ENSRNOG00000032943 | F1LTA3_RAT | 1.16 | 4.47 | 7.65E-28 | 4.17E-26 |
| 5 h | ENSRNOG00000013938 | Tshz3 | 1.99 | 3.71 | 9.79E-28 | 5.32E-26 |
| 5 h | ENSRNOG00000013140 | Pdzd2 | 1.58 | 7.08 | 1.86E-27 | 9.99E-26 |
| 5 h | ENSRNOG00000035516 | rno-mir-132 | 6.68 | -0.07 | 2.23E-27 | 1.19E-25 |
| 5 h | ENSRNOG00000018692 | Mc4r | -3.17 | 1.19 | 2.34E-27 | 1.25E-25 |
| 5 h | ENSRNOG00000007474 | ST7 | 1.21 | 4.46 | 2.36E-27 | 1.25E-25 |
| 5 h | ENSRNOG00000006612 | Map2k3 | 1.76 | 3.40 | 2.69E-27 | 1.42E-25 |
| 5 h | ENSRNOG00000043031 | RGD1305627 | 4.55 | 3.39 | 1.20E-26 | 6.26E-25 |
| 5 h | ENSRNOG00000007923 | Cgref1 | 1.26 | 4.79 | 1.36E-26 | 7.02E-25 |
| 5 h | ENSRNOG00000007078 | Wisp1 | 3.66 | 0.54 | 1.36E-26 | 7.02E-25 |
| 5 h | ENSRNOG00000005033 | Acvr1 | 1.99 | 5.38 | 1.42E-26 | 7.29E-25 |
| 5 h | ENSRNOG00000002946 | Socs3 | 3.02 | 0.72 | 1.75E-26 | 8.90E-25 |
| 5 h | ENSRNOG00000014199 | Znf703 | 1.65 | 2.71 | 2.15E-26 | 1.09E-24 |
| 5 h | ENSRNOG00000008015 | Fos | 3.68 | 6.22 | 2.66E-26 | 1.34E-24 |
| 5 h | ENSRNOG00000008519 | RGD1309079 | -1.61 | 4.11 | 2.91E-26 | 1.46E-24 |
| 5 h | ENSRNOG00000020426 | Erf | 1.60 | 4.92 | 4.47E-26 | 2.22E-24 |
| 5 h | ENSRNOG00000021262 | Slc23a2 | 1.13 | 6.15 | 4.52E-26 | 2.24E-24 |
| 5 h | ENSRNOG00000012442 | RGD1305254 | 2.74 | 6.49 | 4.63E-26 | 2.28E-24 |
| 5 h | ENSRNOG00000037467 | Ffar3 | 4.08 | -0.89 | 4.98E-26 | 2.45E-24 |
| 5 h | ENSRNOG00000006929 | Hnrpll | 1.09 | 5.48 | 5.89E-26 | 2.89E-24 |
| 5 h | ENSRNOG00000017212 | Spsb1 | 1.08 | 4.64 | 6.91E-26 | 3.38E-24 |
| 5 h | ENSRNOG00000006532 | H3f3b | 1.00 | 8.35 | 7.92E-26 | 3.84E-24 |
| 5 h | ENSRNOG00000022772 | Prickle1 | 1.38 | 8.03 | 9.85E-26 | 4.76E-24 |
| 5 h | ENSRNOG00000005049 | Tbr1 | -1.70 | 3.83 | 2.03E-25 | 9.74E-24 |
| 5 h | ENSRNOG00000004303 | Timp3 | 1.08 | 5.74 | 2.65E-25 | 1.26E-23 |
| 5 h | ENSRNOG00000001557 | Cxadr | 1.40 | 4.11 | 2.93E-25 | 1.39E-23 |
| 5 h | ENSRNOG00000009180 | Xkr7 | 3.79 | 2.78 | 3.13E-25 | 1.48E-23 |
| 5 h | ENSRNOG00000043381 | D4A8L6_RAT | -1.01 | 5.10 | 3.66E-25 | 1.73E-23 |
| 5 h | ENSRNOG00000007817 | Kctd6 | 1.48 | 5.59 | 4.34E-25 | 2.04E-23 |
| 5 h | ENSRNOG00000010910 | Gmeb1 | 1.07 | 4.23 | 4.59E-25 | 2.15E-23 |
| 5 h | ENSRNOG00000002159 | Agpat9 | 1.48 | 3.21 | 5.31E-25 | 2.47E-23 |
| 5 h | ENSRNOG00000021256 | Adra1d | -1.49 | 5.76 | 8.24E-25 | 3.81E-23 |
| 5 h | ENSRNOG00000013838 | Hcrtr1 | 3.04 | 0.97 | 9.89E-25 | 4.56E-23 |
| 5 h | ENSRNOG00000010744 | Nrp1 | 1.48 | 7.45 | 1.49E-24 | 6.83E-23 |
| 5 h | ENSRNOG00000014343 | D4A0V6_RAT | -1.49 | 5.39 | 1.66E-24 | 7.53E-23 |
| 5 h | ENSRNOG00000003675 | Ptchd1 | 1.98 | 3.90 | 2.01E-24 | 9.06E-23 |
| 5 h | ENSRNOG00000010306 | LOC100365063 | 1.18 | 5.62 | 5.73E-24 | 2.57E-22 |
| 5 h | ENSRNOG00000001086 | Vps37b | 1.21 | 5.32 | 6.51E-24 | 2.91E-22 |
| 5 h | ENSRNOG00000008187 | Ubash3b | 1.40 | 4.93 | 7.87E-24 | 3.49E-22 |
| 5 h | ENSRNOG00000017539 | Mmp9 | 4.04 | 1.89 | 1.04E-23 | 4.57E-22 |
| 5 h | ENSRNOG00000024945 | Dusp18 | 1.64 | 4.70 | 1.62E-23 | 7.10E-22 |
| 5 h | ENSRNOG00000020782 | Pspc1 | 1.02 | 6.13 | 3.16E-23 | 1.35E-21 |
| 5 h | ENSRNOG00000001110 | N4bp2l1 | -1.17 | 4.23 | 3.31E-23 | 1.41E-21 |
| 5 h | ENSRNOG00000000488 | Hmga1 | 1.15 | 4.82 | 4.04E-23 | 1.72E-21 |
| 5 h | ENSRNOG00000017204 | PQLC1_RAT | -1.41 | 5.30 | 4.61E-23 | 1.95E-21 |
| 5 h | ENSRNOG00000032546 | Dot1l | 1.41 | 4.70 | 6.43E-23 | 2.72E-21 |
| 5 h | ENSRNOG00000002122 | Lrrc8c | 1.36 | 3.30 | 9.56E-23 | 3.98E-21 |
| 5 h | ENSRNOG00000015903 | Add2 | 1.01 | 7.74 | 1.36E-22 | 5.65E-21 |
| 5 h | ENSRNOG00000036816 | WLS_RAT | 1.00 | 5.73 | 2.76E-22 | 1.13E-20 |
| 5 h | ENSRNOG00000010237 | Map7d1 | 1.33 | 7.70 | 3.07E-22 | 1.26E-20 |
| 5 h | ENSRNOG00000021103 | Nrxn2 | 1.18 | 7.37 | 3.14E-22 | 1.28E-20 |
| 5 h | ENSRNOG00000020559 | E9PTV7_RAT | 2.61 | 2.96 | 5.46E-22 | 2.20E-20 |
| 5 h | ENSRNOG00000008041 | RGD1561357 | -1.15 | 5.53 | 6.81E-22 | 2.74E-20 |
| 5 h | ENSRNOG00000012466 | Heca | 1.06 | 6.19 | 7.28E-22 | 2.92E-20 |
| 5 h | ENSRNOG00000009046 | Phf13 | 1.20 | 3.82 | 8.23E-22 | 3.28E-20 |
| 5 h | ENSRNOG00000042191 | Rcor1 | 1.47 | 4.06 | 1.13E-21 | 4.42E-20 |
| 5 h | ENSRNOG00000024461 | Mar-11 | 2.24 | 1.01 | 1.39E-21 | 5.41E-20 |
| 5 h | ENSRNOG00000007754 | Insm2 | 3.08 | -0.67 | 1.40E-21 | 5.41E-20 |
| 5 h | ENSRNOG00000005413 | Creb3l1 | 1.89 | 1.59 | 1.85E-21 | 7.14E-20 |
| 5 h | ENSRNOG00000019134 | Htr4 | 1.28 | 3.69 | 1.92E-21 | 7.38E-20 |
| 5 h | ENSRNOG00000013862 | Dusp2 | 3.19 | 2.38 | 1.96E-21 | 7.50E-20 |
| 5 h | ENSRNOG00000021484 | retrotransposed | 2.06 | 1.43 | 2.25E-21 | 8.59E-20 |
| 5 h | ENSRNOG00000038440 | D3ZF40_RAT | 1.84 | 1.47 | 4.32E-21 | 1.61E-19 |
| 5 h | ENSRNOG00000024832 | Gpr158 | 1.14 | 8.09 | 4.76E-21 | 1.76E-19 |
| 5 h | ENSRNOG00000026447 | D3ZW02_RAT | 1.79 | 5.23 | 5.21E-21 | 1.92E-19 |
| 5 h | ENSRNOG00000025731 | PTMA_RAT | 1.06 | 6.09 | 7.62E-21 | 2.80E-19 |
| 5 h | ENSRNOG00000025406 | LOC100360623 | 1.06 | 6.62 | 7.76E-21 | 2.84E-19 |
| 5 h | ENSRNOG00000009768 | Npy | 1.62 | 4.59 | 1.08E-20 | 3.92E-19 |
| 5 h | ENSRNOG00000018584 | Ptma | 1.05 | 8.38 | 1.53E-20 | 5.47E-19 |
| 5 h | ENSRNOG00000020597 | RGD1305014 | 1.21 | 4.11 | 1.63E-20 | 5.84E-19 |
| 5 h | ENSRNOG00000023579 | D4AC33_RAT | 1.02 | 6.01 | 2.52E-20 | 8.92E-19 |
| 5 h | ENSRNOG00000010868 | Rims4 | 1.22 | 3.35 | 3.46E-20 | 1.21E-18 |
| 5 h | ENSRNOG00000008067 | LOC100363014 | 1.26 | 3.96 | 3.81E-20 | 1.33E-18 |
| 5 h | ENSRNOG00000021540 | RGD1563441 | 1.21 | 6.36 | 4.74E-20 | 1.64E-18 |
| 5 h | ENSRNOG00000037951 | Nhsl2 | -1.17 | 4.36 | 4.75E-20 | 1.64E-18 |
| 5 h | ENSRNOG00000007645 | Kcnj9 | 1.34 | 5.34 | 5.11E-20 | 1.75E-18 |
| 5 h | ENSRNOG00000011803 | Lrfn2 | 1.51 | 5.06 | 5.59E-20 | 1.91E-18 |
| 5 h | ENSRNOG00000001785 | Etv5 | 1.04 | 6.14 | 6.05E-20 | 2.06E-18 |
| 5 h | ENSRNOG00000009412 | Lrch1 | 1.21 | 3.87 | 7.02E-20 | 2.37E-18 |
| 5 h | ENSRNOG00000008943 | Penk | 2.34 | 5.90 | 8.49E-20 | 2.84E-18 |
| 5 h | ENSRNOG00000005776 | Bcl11b | -1.24 | 7.14 | 9.56E-20 | 3.18E-18 |
| 5 h | ENSRNOG00000010712 | Terf2ip | 1.03 | 6.90 | 1.81E-19 | 5.95E-18 |
| 5 h | ENSRNOG00000005159 | RGD1308133 | 1.01 | 5.67 | 2.09E-19 | 6.86E-18 |
| 5 h | ENSRNOG00000018113 | Anln | -1.42 | 5.40 | 3.07E-19 | 9.95E-18 |
| 5 h | ENSRNOG00000028992 | Acan | 1.28 | 3.97 | 3.43E-19 | 1.11E-17 |
| 5 h | ENSRNOG00000014037 | Dcun1d3 | 1.19 | 2.82 | 4.86E-19 | 1.56E-17 |
| 5 h | ENSRNOG00000016011 | Plekhg1 | 1.26 | 5.44 | 5.53E-19 | 1.77E-17 |
| 5 h | ENSRNOG00000016110 | Kcnk12 | 1.72 | 2.65 | 6.71E-19 | 2.14E-17 |
| 5 h | ENSRNOG00000025587 | Plagl1 | 1.73 | 2.59 | 6.97E-19 | 2.22E-17 |
| 5 h | ENSRNOG00000014963 | GPR56_RAT | 1.48 | 7.09 | 8.73E-19 | 2.76E-17 |
| 5 h | ENSRNOG00000024089 | Fndc3b | 1.59 | 5.13 | 1.06E-18 | 3.32E-17 |
| 5 h | ENSRNOG00000021984 | Rgs7 | 1.02 | 7.00 | 1.37E-18 | 4.25E-17 |
| 5 h | ENSRNOG00000036699 | RGD1306926 | -1.92 | 4.21 | 1.53E-18 | 4.73E-17 |
| 5 h | ENSRNOG00000033973 | RGD1561115 | -1.94 | 2.48 | 1.72E-18 | 5.29E-17 |
| 5 h | ENSRNOG00000026793 | Nt5dc3 | 1.29 | 6.96 | 2.05E-18 | 6.28E-17 |
| 5 h | ENSRNOG00000009347 | Arhgap25 | 1.31 | 3.40 | 2.80E-18 | 8.56E-17 |
| 5 h | ENSRNOG00000029812 | Mex3a | 3.06 | 0.50 | 3.52E-18 | 1.07E-16 |
| 5 h | ENSRNOG00000012036 | PCSK5_RAT | 1.12 | 5.20 | 6.69E-18 | 2.01E-16 |
| 5 h | ENSRNOG00000016874 | Znf521 | 1.24 | 4.44 | 8.16E-18 | 2.43E-16 |
| 5 h | ENSRNOG00000002980 | LOC100360406 | 1.16 | 5.79 | 8.30E-18 | 2.47E-16 |
| 5 h | ENSRNOG00000024055 | Dmkn | 3.81 | -1.24 | 1.04E-17 | 3.04E-16 |
| 5 h | ENSRNOG00000024970 | F1M5L7_RAT | 1.05 | 4.35 | 1.15E-17 | 3.36E-16 |
| 5 h | ENSRNOG00000001843 | Bcl6 | -1.18 | 5.99 | 1.32E-17 | 3.85E-16 |
| 5 h | ENSRNOG00000012284 | Khdrbs2 | 1.42 | 4.30 | 1.64E-17 | 4.76E-16 |
| 5 h | ENSRNOG00000018094 | Sv2c | 1.31 | 3.53 | 2.39E-17 | 6.88E-16 |
| 5 h | ENSRNOG00000001184 | Pknox1 | 1.04 | 3.05 | 3.09E-17 | 8.82E-16 |
| 5 h | ENSRNOG00000010417 | D3ZHS3_RAT | 1.15 | 4.12 | 3.19E-17 | 9.06E-16 |
| 5 h | ENSRNOG00000025624 | Arhgap20 | -1.53 | 6.88 | 3.22E-17 | 9.15E-16 |
| 5 h | ENSRNOG00000021285 | F1MAS4_RAT | 1.10 | 4.87 | 3.62E-17 | 1.02E-15 |
| 5 h | ENSRNOG00000018927 | Sprn | -1.22 | 3.99 | 4.02E-17 | 1.13E-15 |
| 5 h | ENSRNOG00000001004 | Ncor2 | 1.19 | 6.85 | 4.82E-17 | 1.35E-15 |
| 5 h | ENSRNOG00000035620 | rno-mir-22 | 1.50 | 1.86 | 7.06E-17 | 1.97E-15 |
| 5 h | ENSRNOG00000020879 | Nags | 2.73 | -0.28 | 1.10E-16 | 3.00E-15 |
| 5 h | ENSRNOG00000030914 | RGD1559493 | 3.54 | -1.40 | 1.09E-16 | 3.00E-15 |
| 5 h | ENSRNOG00000016728 | LOC100362710 | -2.24 | 5.58 | 1.31E-16 | 3.58E-15 |
| 5 h | ENSRNOG00000021691 | Ccdc92 | 1.19 | 6.10 | 1.33E-16 | 3.63E-15 |
| 5 h | ENSRNOG00000030397 | Zbtb1 | 1.09 | 3.42 | 1.47E-16 | 4.00E-15 |
| 5 h | ENSRNOG00000016950 | Otud1 | 1.98 | 3.13 | 1.54E-16 | 4.16E-15 |
| 5 h | ENSRNOG00000011668 | Nfil3 | 1.46 | 3.56 | 1.66E-16 | 4.46E-15 |
| 5 h | ENSRNOG00000002075 | Cnot6l | 1.02 | 5.85 | 1.69E-16 | 4.53E-15 |
| 5 h | ENSRNOG00000005609 | Neurod1 | -1.94 | 5.06 | 2.16E-16 | 5.76E-15 |
| 5 h | ENSRNOG00000033528 | Tll1 | 1.43 | 4.03 | 2.24E-16 | 5.96E-15 |
| 5 h | ENSRNOG00000003745 | Atf3 | 3.16 | 5.03 | 2.37E-16 | 6.28E-15 |
| 5 h | ENSRNOG00000016099 | Id4 | 1.34 | 5.75 | 2.84E-16 | 7.49E-15 |
| 5 h | ENSRNOG00000001598 | Usp16 | 1.14 | 4.88 | 3.49E-16 | 9.18E-15 |
| 5 h | ENSRNOG00000004622 | Calcrl | 1.08 | 3.94 | 3.92E-16 | 1.02E-14 |
| 5 h | ENSRNOG00000027590 | Jakmip3 | -1.10 | 4.06 | 3.92E-16 | 1.02E-14 |
| 5 h | ENSRNOG00000042899 | Fcgbpl1 | 3.86 | -1.46 | 5.40E-16 | 1.40E-14 |
| 5 h | ENSRNOG00000011720 | D3Z9L4_RAT | 1.18 | 4.72 | 5.61E-16 | 1.45E-14 |
| 5 h | ENSRNOG00000001877 | Med15 | 1.21 | 5.23 | 6.21E-16 | 1.59E-14 |
| 5 h | ENSRNOG00000023197 | Znf608 | 1.18 | 5.17 | 6.29E-16 | 1.61E-14 |
| 5 h | ENSRNOG00000009946 | Ldlr | -1.04 | 6.03 | 9.60E-16 | 2.40E-14 |
| 5 h | ENSRNOG00000007483 | Ccnf | 1.41 | 2.88 | 9.72E-16 | 2.43E-14 |
| 5 h | ENSRNOG00000002930 | Ppl | -2.93 | 4.64 | 1.35E-15 | 3.36E-14 |
| 5 h | ENSRNOG00000026293 | Jun | 1.52 | 7.49 | 1.54E-15 | 3.82E-14 |
| 5 h | ENSRNOG00000015382 | Arid5a | 1.44 | 4.66 | 1.59E-15 | 3.93E-14 |
| 5 h | ENSRNOG00000006388 | Pygl | 2.29 | 2.88 | 1.68E-15 | 4.13E-14 |
| 5 h | ENSRNOG00000007843 | Wnt2 | 2.82 | 3.32 | 1.92E-15 | 4.73E-14 |
| 5 h | ENSRNOG00000020448 | Ncan | 1.00 | 8.15 | 2.08E-15 | 5.09E-14 |
| 5 h | ENSRNOG00000021744 | ENSRNOG00000021744 | 3.61 | -1.02 | 2.94E-15 | 7.17E-14 |
| 5 h | ENSRNOG00000022712 | Bend7 | 1.67 | 1.16 | 3.76E-15 | 9.03E-14 |
| 5 h | ENSRNOG00000014548 | Nedd9 | 1.33 | 6.83 | 3.78E-15 | 9.06E-14 |
| 5 h | ENSRNOG00000007707 | Znf467 | -1.64 | 4.00 | 4.37E-15 | 1.04E-13 |
| 5 h | ENSRNOG00000015719 | Egr4 | 2.26 | 4.69 | 4.41E-15 | 1.05E-13 |
| 5 h | ENSRNOG00000031801 | Ephb3 | 1.72 | 3.03 | 5.14E-15 | 1.22E-13 |
| 5 h | ENSRNOG00000039837 | RGD1563945 | 1.26 | 2.84 | 5.26E-15 | 1.25E-13 |
| 5 h | ENSRNOG00000032019 | Bend4 | 1.38 | 2.06 | 5.31E-15 | 1.26E-13 |
| 5 h | ENSRNOG00000037299 | LOC100359829 | -2.18 | 1.75 | 5.47E-15 | 1.29E-13 |
| 5 h | ENSRNOG00000006663 | Usp2 | 1.05 | 5.69 | 7.60E-15 | 1.78E-13 |
| 5 h | ENSRNOG00000022730 | Zfp57 | -1.34 | 3.76 | 8.67E-15 | 2.03E-13 |
| 5 h | ENSRNOG00000002202 | Ankrd56 | 3.84 | 1.72 | 9.23E-15 | 2.15E-13 |
| 5 h | ENSRNOG00000022598 | Trerf1 | -1.22 | 3.90 | 9.28E-15 | 2.16E-13 |
| 5 h | ENSRNOG00000009528 | Sdcbp2 | 3.51 | 0.12 | 9.57E-15 | 2.21E-13 |
| 5 h | ENSRNOG00000005148 | Scrt2 | 1.59 | 1.32 | 1.20E-14 | 2.77E-13 |
| 5 h | ENSRNOG00000018674 | Ntrk3 | 1.06 | 7.10 | 1.28E-14 | 2.94E-13 |
| 5 h | ENSRNOG00000021324 | RGD1565059 | -1.41 | 2.59 | 2.13E-14 | 4.82E-13 |
| 5 h | ENSRNOG00000025804 | Fastkd5 | 1.14 | 3.60 | 2.37E-14 | 5.35E-13 |
| 5 h | ENSRNOG00000020164 | RGD1563319 | 1.02 | 3.44 | 3.46E-14 | 7.71E-13 |
| 5 h | ENSRNOG00000009157 | Fut4 | -2.38 | 0.42 | 4.48E-14 | 9.86E-13 |
| 5 h | ENSRNOG00000030382 | ENSRNOG00000030382 | -1.99 | 1.29 | 4.91E-14 | 1.08E-12 |
| 5 h | ENSRNOG00000006789 | Ddit3 | 1.15 | 5.97 | 5.39E-14 | 1.18E-12 |
| 5 h | ENSRNOG00000008412 | Gprc5a | 3.31 | -0.89 | 5.75E-14 | 1.25E-12 |
| 5 h | ENSRNOG00000025394 | TANC1_RAT | 1.29 | 8.31 | 6.35E-14 | 1.38E-12 |
| 5 h | ENSRNOG00000038540 | Gp5 | 1.75 | 1.86 | 6.51E-14 | 1.42E-12 |
| 5 h | ENSRNOG00000028190 | D4ACF8_RAT | -2.84 | 1.22 | 6.72E-14 | 1.46E-12 |
| 5 h | ENSRNOG00000037476 | Galnt9 | 1.24 | 6.84 | 7.65E-14 | 1.65E-12 |
| 5 h | ENSRNOG00000010984 | Anxa11 | 1.02 | 7.69 | 1.01E-13 | 2.15E-12 |
| 5 h | ENSRNOG00000005781 | Wnt16 | -1.84 | 1.05 | 1.60E-13 | 3.39E-12 |
| 5 h | ENSRNOG00000001198 | RGD1565883 | 1.75 | 1.38 | 1.80E-13 | 3.78E-12 |
| 5 h | ENSRNOG00000021091 | Trank1 | 1.49 | 7.28 | 1.84E-13 | 3.87E-12 |
| 5 h | ENSRNOG00000001149 | Pxn | 1.00 | 4.83 | 1.98E-13 | 4.16E-12 |
| 5 h | ENSRNOG00000038375 | retrotransposed | 1.21 | 2.21 | 2.19E-13 | 4.58E-12 |
| 5 h | ENSRNOG00000020424 | Ppapdc1a | 2.20 | 4.14 | 3.02E-13 | 6.25E-12 |
| 5 h | ENSRNOG00000014172 | Npy5r | -1.38 | 2.96 | 3.09E-13 | 6.37E-12 |
| 5 h | ENSRNOG00000019716 | Ntf3 | -2.27 | 4.06 | 3.84E-13 | 7.83E-12 |
| 5 h | ENSRNOG00000036827 | Ppp1r1a | 1.22 | 6.61 | 3.96E-13 | 8.07E-12 |
| 5 h | ENSRNOG00000028584 | Alkbh2 | -1.18 | 2.41 | 4.05E-13 | 8.22E-12 |
| 5 h | ENSRNOG00000002036 | LOC681004 | 1.82 | 6.08 | 6.08E-13 | 1.22E-11 |
| 5 h | ENSRNOG00000004828 | Acvr1c | 1.54 | 3.80 | 6.13E-13 | 1.23E-11 |
| 5 h | ENSRNOG00000002456 | LOC690286 | -1.08 | 2.91 | 6.16E-13 | 1.23E-11 |
| 5 h | ENSRNOG00000030318 | Gpr83 | 1.03 | 4.75 | 6.71E-13 | 1.34E-11 |
| 5 h | ENSRNOG00000011238 | D3ZMH5_RAT | 1.00 | 6.60 | 8.62E-13 | 1.71E-11 |
| 5 h | ENSRNOG00000004870 | Dyrk3 | 1.32 | 3.72 | 9.21E-13 | 1.82E-11 |
| 5 h | ENSRNOG00000020193 | Runx2 | 2.47 | 0.15 | 1.75E-12 | 3.38E-11 |
| 5 h | ENSRNOG00000004744 | Fam84b | -2.38 | 1.44 | 2.72E-12 | 5.11E-11 |
| 5 h | ENSRNOG00000002004 | D4A123_RAT | -1.44 | 6.00 | 3.34E-12 | 6.20E-11 |
| 5 h | ENSRNOG00000016500 | RGD1563891 | -2.51 | 2.85 | 3.62E-12 | 6.68E-11 |
| 5 h | ENSRNOG00000009354 | Nrarp | 1.03 | 4.07 | 4.13E-12 | 7.59E-11 |
| 5 h | ENSRNOG00000016541 | Enc1 | 1.12 | 10.18 | 4.13E-12 | 7.59E-11 |
| 5 h | ENSRNOG00000018938 | Mkx | -2.30 | 2.60 | 4.69E-12 | 8.59E-11 |
| 5 h | ENSRNOG00000006689 | D3ZAP7_RAT | 1.08 | 5.12 | 4.85E-12 | 8.87E-11 |
| 5 h | ENSRNOG00000015416 | Obfc2a | 1.08 | 4.22 | 5.10E-12 | 9.28E-11 |
| 5 h | ENSRNOG00000004483 | Ptprr | -1.04 | 4.87 | 6.36E-12 | 1.14E-10 |
| 5 h | ENSRNOG00000017489 | Gse1 | 1.44 | 6.17 | 6.67E-12 | 1.20E-10 |
| 5 h | ENSRNOG00000011788 | RGD1566029 | 1.37 | 3.30 | 7.60E-12 | 1.35E-10 |
| 5 h | ENSRNOG00000017189 | KCNG2_RAT | -1.29 | 4.16 | 8.14E-12 | 1.44E-10 |
| 5 h | ENSRNOG00000010794 | Dennd3 | 1.37 | 2.99 | 9.05E-12 | 1.60E-10 |
| 5 h | ENSRNOG00000014259 | Mycl1 | -1.56 | 4.40 | 1.46E-11 | 2.53E-10 |
| 5 h | ENSRNOG00000042960 | Rgc32 | 1.14 | 3.27 | 1.48E-11 | 2.57E-10 |
| 5 h | ENSRNOG00000013202 | Akap7 | -1.15 | 6.09 | 1.53E-11 | 2.65E-10 |
| 5 h | ENSRNOG00000011568 | Rspo3 | -1.82 | 3.42 | 1.96E-11 | 3.38E-10 |
| 5 h | ENSRNOG00000002345 | LOC100361238 | 1.17 | 4.43 | 2.49E-11 | 4.26E-10 |
| 5 h | ENSRNOG00000005531 | Tyro3 | 1.19 | 6.66 | 4.20E-11 | 6.97E-10 |
| 5 h | ENSRNOG00000001793 | F1M3W7_RAT | 1.28 | 6.47 | 4.53E-11 | 7.50E-10 |
| 5 h | ENSRNOG00000022244 | Olr1462 | -2.34 | 1.02 | 7.45E-11 | 1.20E-09 |
| 5 h | ENSRNOG00000043378 | Nkpd1 | 1.72 | 1.28 | 9.36E-11 | 1.49E-09 |
| 5 h | ENSRNOG00000005124 | Plekhh2 | 1.30 | 5.12 | 9.66E-11 | 1.53E-09 |
| 5 h | ENSRNOG00000012280 | Ptx3 | 2.07 | 1.59 | 1.07E-10 | 1.69E-09 |
| 5 h | ENSRNOG00000012708 | RGD1563020 | 1.55 | 1.56 | 1.07E-10 | 1.69E-09 |
| 5 h | ENSRNOG00000015495 | Slc25a37 | 1.35 | 1.56 | 1.10E-10 | 1.74E-09 |
| 5 h | ENSRNOG00000003888 | Rgs13 | -2.82 | 1.56 | 1.14E-10 | 1.79E-09 |
| 5 h | ENSRNOG00000008270 | Cntrob | 1.53 | 1.53 | 1.19E-10 | 1.86E-09 |
| 5 h | ENSRNOG00000036880 | Arl5c | 2.59 | 0.22 | 1.58E-10 | 2.45E-09 |
| 5 h | ENSRNOG00000006622 | Cry1 | 1.03 | 3.75 | 1.60E-10 | 2.47E-09 |
| 5 h | ENSRNOG00000009802 | LOC100362888 | -1.42 | 2.41 | 1.77E-10 | 2.72E-09 |
| 5 h | ENSRNOG00000035032 | U5 | 1.55 | 0.88 | 2.05E-10 | 3.12E-09 |
| 5 h | ENSRNOG00000030180 | Lrrc10b | 1.19 | 5.96 | 2.56E-10 | 3.85E-09 |
| 5 h | ENSRNOG00000001252 | Chst12 | -1.05 | 3.38 | 2.83E-10 | 4.26E-09 |
| 5 h | ENSRNOG00000006670 | Rai2 | 1.14 | 2.92 | 2.84E-10 | 4.27E-09 |
| 5 h | ENSRNOG00000013436 | Pde7b | 1.60 | 5.17 | 2.89E-10 | 4.33E-09 |
| 5 h | ENSRNOG00000021234 | D3ZVP9_RAT | 1.92 | 2.58 | 2.91E-10 | 4.36E-09 |
| 5 h | ENSRNOG00000042874 | F1M1K7_RAT | 1.85 | 0.26 | 3.12E-10 | 4.67E-09 |
| 5 h | ENSRNOG00000015655 | Ptgfrn | 1.12 | 4.86 | 3.17E-10 | 4.73E-09 |
| 5 h | ENSRNOG00000030101 | Traip | -1.20 | 3.12 | 3.46E-10 | 5.14E-09 |
| 5 h | ENSRNOG00000017659 | Hs3st2 | 3.16 | 1.41 | 3.88E-10 | 5.74E-09 |
| 5 h | ENSRNOG00000008758 | Tspan18 | -1.33 | 4.46 | 4.11E-10 | 6.06E-09 |
| 5 h | ENSRNOG00000032878 | CXXC5_RAT | 1.02 | 5.79 | 4.45E-10 | 6.55E-09 |
| 5 h | ENSRNOG00000028099 | retrotransposed | 1.55 | 0.69 | 4.86E-10 | 7.14E-09 |
| 5 h | ENSRNOG00000006110 | Jph1 | -1.36 | 4.82 | 7.27E-10 | 1.04E-08 |
| 5 h | ENSRNOG00000039897 | F1M636_RAT | 1.68 | 0.20 | 8.09E-10 | 1.15E-08 |
| 5 h | ENSRNOG00000031368 | LOC499235 | 1.04 | 1.94 | 8.25E-10 | 1.18E-08 |
| 5 h | ENSRNOG00000014566 | Fkbp11 | 1.26 | 1.81 | 8.94E-10 | 1.27E-08 |
| 5 h | ENSRNOG00000016246 | Tshz1 | 1.10 | 5.92 | 1.08E-09 | 1.52E-08 |
| 5 h | ENSRNOG00000018116 | Kcna3 | 1.24 | 3.37 | 1.08E-09 | 1.52E-08 |
| 5 h | ENSRNOG00000015441 | Il4ra | -1.06 | 4.59 | 1.17E-09 | 1.64E-08 |
| 5 h | ENSRNOG00000015667 | Epcam | 1.91 | -0.35 | 1.23E-09 | 1.71E-08 |
| 5 h | ENSRNOG00000010516 | Plau | -2.01 | 1.64 | 1.23E-09 | 1.72E-08 |
| 5 h | ENSRNOG00000036828 | Pde1b | -1.22 | 6.49 | 1.25E-09 | 1.74E-08 |
| 5 h | ENSRNOG00000020652 | Tgfb1 | 1.58 | 0.83 | 1.34E-09 | 1.86E-08 |
| 5 h | ENSRNOG00000014149 | Npy1r | -1.30 | 4.00 | 1.41E-09 | 1.95E-08 |
| 5 h | ENSRNOG00000010011 | Osbpl3 | 1.39 | 2.39 | 1.46E-09 | 2.02E-08 |
| 5 h | ENSRNOG00000020729 | Stc2 | 1.80 | -0.12 | 1.69E-09 | 2.30E-08 |
| 5 h | ENSRNOG00000016925 | Rbm24 | -1.34 | 1.64 | 2.00E-09 | 2.70E-08 |
| 5 h | ENSRNOG00000007533 | Rab27a | -1.62 | 3.50 | 2.10E-09 | 2.82E-08 |
| 5 h | ENSRNOG00000016225 | Fgd3 | 1.05 | 2.65 | 2.23E-09 | 2.98E-08 |
| 5 h | ENSRNOG00000021128 | Kcnj11 | -1.06 | 2.67 | 2.32E-09 | 3.08E-08 |
| 5 h | ENSRNOG00000008125 | Igbp1b | -2.95 | -0.33 | 2.42E-09 | 3.20E-08 |
| 5 h | ENSRNOG00000021568 | Ccdc110 | 2.18 | -0.36 | 2.48E-09 | 3.27E-08 |
| 5 h | ENSRNOG00000010959 | Klhl25 | 1.13 | 2.49 | 2.56E-09 | 3.37E-08 |
| 5 h | ENSRNOG00000023352 | Fam78a | 1.42 | 0.50 | 2.74E-09 | 3.60E-08 |
| 5 h | ENSRNOG00000006588 | Meox2 | -3.18 | 0.20 | 3.35E-09 | 4.31E-08 |
| 5 h | ENSRNOG00000004084 | Fam84a | 1.01 | 8.13 | 4.01E-09 | 5.10E-08 |
| 5 h | ENSRNOG00000005275 | Shmt1 | -1.19 | 1.94 | 6.09E-09 | 7.56E-08 |
| 5 h | ENSRNOG00000004207 | EMAL5_RAT | 1.15 | 6.76 | 6.14E-09 | 7.60E-08 |
| 5 h | ENSRNOG00000002092 | Hunk | 1.75 | 2.90 | 6.40E-09 | 7.92E-08 |
| 5 h | ENSRNOG00000012216 | Tgfbi | 1.25 | 1.56 | 6.57E-09 | 8.11E-08 |
| 5 h | ENSRNOG00000034140 | Htra4 | 3.36 | 4.57 | 6.78E-09 | 8.34E-08 |
| 5 h | ENSRNOG00000019451 | Smtn | 1.12 | 2.42 | 6.97E-09 | 8.55E-08 |
| 5 h | ENSRNOG00000042724 | Slc7a14 | 1.04 | 4.56 | 6.99E-09 | 8.58E-08 |
| 5 h | ENSRNOG00000026435 | Arid3a | -1.19 | 2.19 | 7.42E-09 | 9.08E-08 |
| 5 h | ENSRNOG00000005956 | LOC681820 | -1.41 | 2.09 | 7.95E-09 | 9.65E-08 |
| 5 h | ENSRNOG00000017093 | RGD1311307 | -1.37 | 3.59 | 8.59E-09 | 1.04E-07 |
| 5 h | ENSRNOG00000023473 | RGD1561238 | 2.22 | 0.68 | 9.09E-09 | 1.09E-07 |
| 5 h | ENSRNOG00000041417 | U4atac | 1.23 | 1.31 | 9.74E-09 | 1.17E-07 |
| 5 h | ENSRNOG00000021731 | N4bp3 | 1.40 | 1.70 | 9.77E-09 | 1.17E-07 |
| 5 h | ENSRNOG00000011901 | Rrad | 2.25 | 0.60 | 1.16E-08 | 1.38E-07 |
| 5 h | ENSRNOG00000009870 | Tmem88 | 1.47 | 0.74 | 1.19E-08 | 1.41E-07 |
| 5 h | ENSRNOG00000019673 | Zfp36 | 1.16 | 2.47 | 1.41E-08 | 1.66E-07 |
| 5 h | ENSRNOG00000016222 | RGD1308930 | 1.17 | 2.40 | 1.51E-08 | 1.76E-07 |
| 5 h | ENSRNOG00000028137 | D4A0Y6_RAT | 1.50 | 1.85 | 1.69E-08 | 1.95E-07 |
| 5 h | ENSRNOG00000015374 | LOC100362059 | 1.46 | 0.59 | 1.75E-08 | 2.02E-07 |
| 5 h | ENSRNOG00000016623 | Tmeff2 | 1.01 | 6.91 | 1.77E-08 | 2.04E-07 |
| 5 h | ENSRNOG00000027784 | Tsku | 2.17 | -0.28 | 1.81E-08 | 2.08E-07 |
| 5 h | ENSRNOG00000013647 | Polm | -1.41 | 1.90 | 1.93E-08 | 2.20E-07 |
| 5 h | ENSRNOG00000011101 | Twist1 | -1.49 | 0.98 | 2.24E-08 | 2.54E-07 |
| 5 h | ENSRNOG00000006204 | Slc30a3 | 1.12 | 7.62 | 2.27E-08 | 2.56E-07 |
| 5 h | ENSRNOG00000013851 | Spry4 | 1.23 | 2.83 | 2.68E-08 | 3.01E-07 |
| 5 h | ENSRNOG00000009173 | Smad6 | 1.03 | 1.23 | 2.80E-08 | 3.13E-07 |
| 5 h | ENSRNOG00000018557 | D3ZWM4_RAT | 1.25 | 3.78 | 3.06E-08 | 3.41E-07 |
| 5 h | ENSRNOG00000026944 | Gpr150 | -1.47 | 1.12 | 3.93E-08 | 4.30E-07 |
| 5 h | ENSRNOG00000037695 | Sgpp2 | -1.01 | 4.02 | 4.04E-08 | 4.41E-07 |
| 5 h | ENSRNOG00000023492 | RGD1566021 | 1.08 | 2.31 | 4.07E-08 | 4.44E-07 |
| 5 h | ENSRNOG00000003927 | Cd55 | -1.75 | 2.51 | 4.24E-08 | 4.61E-07 |
| 5 h | ENSRNOG00000029292 | LOC500877 | -3.01 | 0.39 | 4.43E-08 | 4.81E-07 |
| 5 h | ENSRNOG00000000471 | B3galt4 | -1.23 | 1.49 | 4.70E-08 | 5.07E-07 |
| 5 h | ENSRNOG00000007607 | Nr4a1 | 1.04 | 5.91 | 4.76E-08 | 5.14E-07 |
| 5 h | ENSRNOG00000016551 | Bcl2l11 | 1.23 | 0.70 | 4.83E-08 | 5.20E-07 |
| 5 h | ENSRNOG00000007262 | Ccdc134 | -1.10 | 2.63 | 4.87E-08 | 5.23E-07 |
| 5 h | ENSRNOG00000042717 | RGD1566380 | -1.37 | 3.14 | 4.90E-08 | 5.26E-07 |
| 5 h | ENSRNOG00000014971 | Mas1 | 1.22 | 6.41 | 4.98E-08 | 5.33E-07 |
| 5 h | ENSRNOG00000025184 | Prss35 | 1.33 | 4.03 | 5.22E-08 | 5.56E-07 |
| 5 h | ENSRNOG00000016385 | Gabrd | -1.33 | 4.40 | 5.45E-08 | 5.79E-07 |
| 5 h | ENSRNOG00000008259 | Clec1a | -1.32 | 3.49 | 5.81E-08 | 6.15E-07 |
| 5 h | ENSRNOG00000007822 | Vgll4 | 1.13 | 4.89 | 6.45E-08 | 6.77E-07 |
| 5 h | ENSRNOG00000042003 | SNORD113 | 1.47 | 2.03 | 6.60E-08 | 6.91E-07 |
| 5 h | ENSRNOG00000007300 | C1qtnf6 | -1.98 | 1.16 | 9.54E-08 | 9.74E-07 |
| 5 h | ENSRNOG00000034177 | Efna5 | 1.32 | 0.31 | 9.55E-08 | 9.74E-07 |
| 5 h | ENSRNOG00000013869 | Kcnj4 | 1.01 | 5.26 | 1.27E-07 | 1.27E-06 |
| 5 h | ENSRNOG00000005888 | Fsip1 | -1.26 | 0.75 | 1.31E-07 | 1.31E-06 |
| 5 h | ENSRNOG00000022204 | D3ZXV5_RAT | -1.20 | 4.80 | 1.33E-07 | 1.33E-06 |
| 5 h | ENSRNOG00000010047 | LOC100363484 | -2.57 | 0.92 | 1.45E-07 | 1.43E-06 |
| 5 h | ENSRNOG00000018785 | Slc16a13 | -1.17 | 2.34 | 1.58E-07 | 1.56E-06 |
| 5 h | ENSRNOG00000013922 | Dok2 | 1.38 | 0.31 | 1.71E-07 | 1.67E-06 |
| 5 h | ENSRNOG00000033256 | LOC691141 | 1.73 | -0.82 | 1.81E-07 | 1.76E-06 |
| 5 h | ENSRNOG00000003480 | D4A9W0_RAT | -1.61 | 0.55 | 2.02E-07 | 1.95E-06 |
| 5 h | ENSRNOG00000014867 | Synpo2 | -1.16 | 1.59 | 2.39E-07 | 2.29E-06 |
| 5 h | ENSRNOG00000014290 | Grm1 | -1.00 | 7.06 | 2.44E-07 | 2.33E-06 |
| 5 h | ENSRNOG00000024310 | Kcnf1 | 1.60 | 2.88 | 2.66E-07 | 2.53E-06 |
| 5 h | ENSRNOG00000002439 | Inhbb | 2.90 | 0.31 | 2.92E-07 | 2.76E-06 |
| 5 h | ENSRNOG00000020009 | Npas4 | 2.64 | 4.36 | 3.27E-07 | 3.07E-06 |
| 5 h | ENSRNOG00000038606 | F1LVF1_RAT | -1.48 | 0.56 | 3.41E-07 | 3.18E-06 |
| 5 h | ENSRNOG00000043687 | snoU13 | 1.85 | -0.56 | 3.60E-07 | 3.35E-06 |
| 5 h | ENSRNOG00000004889 | Vangl2 | -1.47 | 3.75 | 3.62E-07 | 3.36E-06 |
| 5 h | ENSRNOG00000011994 | Perp | 1.92 | 4.87 | 4.23E-07 | 3.90E-06 |
| 5 h | ENSRNOG00000008027 | Murc | 1.08 | 1.66 | 4.38E-07 | 4.03E-06 |
| 5 h | ENSRNOG00000025846 | LOC690082 | 1.23 | 0.12 | 4.44E-07 | 4.07E-06 |
| 5 h | ENSRNOG00000021745 | Bhlhe22 | -1.07 | 7.85 | 4.61E-07 | 4.20E-06 |
| 5 h | ENSRNOG00000007431 | F1M0U8_RAT | -1.09 | 2.68 | 5.16E-07 | 4.66E-06 |
| 5 h | ENSRNOG00000030299 | LOC683302 | 1.45 | 0.21 | 5.16E-07 | 4.66E-06 |
| 5 h | ENSRNOG00000037901 | D4A8J6_RAT | 1.32 | 1.43 | 5.46E-07 | 4.91E-06 |
| 5 h | ENSRNOG00000015419 | Spem1 | 1.15 | 0.70 | 5.65E-07 | 5.07E-06 |
| 5 h | ENSRNOG00000024065 | Znf575 | -1.26 | 0.96 | 6.31E-07 | 5.61E-06 |
| 5 h | ENSRNOG00000001427 | Orai2 | -1.04 | 5.66 | 6.64E-07 | 5.87E-06 |
| 5 h | ENSRNOG00000015166 | D3ZUC3_RAT | -1.54 | 0.68 | 7.91E-07 | 6.92E-06 |
| 5 h | ENSRNOG00000019447 | Ecel1 | 2.04 | -0.03 | 9.03E-07 | 7.86E-06 |
| 5 h | ENSRNOG00000008526 | Pdzd3 | -1.23 | 1.85 | 1.17E-06 | 9.93E-06 |
| 5 h | ENSRNOG00000000906 | RGD1307396 | -1.42 | 2.73 | 1.44E-06 | 1.20E-05 |
| 5 h | ENSRNOG00000005978 | DOC2B_RAT | 1.04 | 4.76 | 1.50E-06 | 1.25E-05 |
| 5 h | ENSRNOG00000003374 | Ccdc85a | -1.38 | 6.24 | 1.57E-06 | 1.30E-05 |
| 5 h | ENSRNOG00000008930 | Tnfsf15 | -1.88 | 0.69 | 1.74E-06 | 1.44E-05 |
| 5 h | ENSRNOG00000028097 | Slc7a14 | 1.00 | 5.13 | 1.78E-06 | 1.47E-05 |
| 5 h | ENSRNOG00000028530 | D4A6L2_RAT | 1.07 | 1.08 | 1.79E-06 | 1.47E-05 |
| 5 h | ENSRNOG00000018760 | Mpp7 | -1.26 | 3.73 | 1.97E-06 | 1.61E-05 |
| 5 h | ENSRNOG00000039315 | LOC499781 | 1.53 | -0.42 | 2.09E-06 | 1.70E-05 |
| 5 h | ENSRNOG00000020570 | Spred3 | 1.01 | 2.54 | 2.29E-06 | 1.85E-05 |
| 5 h | ENSRNOG00000013215 | DCTD_RAT | -1.10 | 2.43 | 2.65E-06 | 2.10E-05 |
| 5 h | ENSRNOG00000001414 | Serpine1 | 1.80 | 0.42 | 2.83E-06 | 2.24E-05 |
| 5 h | ENSRNOG00000022724 | F1M5M4_RAT | -1.09 | 2.87 | 2.90E-06 | 2.29E-05 |
| 5 h | ENSRNOG00000008168 | Wnt5b | 1.26 | 0.32 | 2.96E-06 | 2.33E-05 |
| 5 h | ENSRNOG00000021434 | RGD1562211 | -1.79 | 0.06 | 3.31E-06 | 2.58E-05 |
| 5 h | ENSRNOG00000039723 | D3ZP37_RAT | 1.29 | 0.51 | 3.32E-06 | 2.59E-05 |
| 5 h | ENSRNOG00000017455 | Wnt10a | 1.24 | 1.05 | 4.50E-06 | 3.45E-05 |
| 5 h | ENSRNOG00000012311 | Slc35d3 | 1.34 | 1.55 | 4.84E-06 | 3.69E-05 |
| 5 h | ENSRNOG00000004229 | Tac2 | 1.20 | 2.31 | 5.59E-06 | 4.20E-05 |
| 5 h | ENSRNOG00000006723 | Itga11 | -2.45 | 3.16 | 5.59E-06 | 4.20E-05 |
| 5 h | ENSRNOG00000011407 | Pragmin | -1.03 | 4.31 | 5.59E-06 | 4.20E-05 |
| 5 h | ENSRNOG00000001547 | Agps | -1.22 | 0.74 | 8.59E-06 | 6.20E-05 |
| 5 h | ENSRNOG00000010254 | Htr1a | -1.09 | 4.37 | 1.04E-05 | 7.40E-05 |
| 5 h | ENSRNOG00000019093 | Nrg2 | 1.21 | 2.71 | 1.05E-05 | 7.44E-05 |
| 5 h | ENSRNOG00000020208 | Gdf10 | -1.68 | 3.59 | 1.08E-05 | 7.64E-05 |
| 5 h | ENSRNOG00000025874 | Efcab4b | -1.96 | 0.07 | 1.10E-05 | 7.79E-05 |
| 5 h | ENSRNOG00000034978 | SNORD113 | 1.80 | -0.56 | 1.22E-05 | 8.51E-05 |
| 5 h | ENSRNOG00000024952 | Atp8b1 | -1.56 | 0.57 | 1.25E-05 | 8.73E-05 |
| 5 h | ENSRNOG00000010553 | Pnma1 | -1.10 | 1.55 | 1.55E-05 | 0.000106108 |
| 5 h | ENSRNOG00000034363 | SNORD113 | 1.14 | 1.58 | 1.59E-05 | 0.000108237 |
| 5 h | ENSRNOG00000013152 | Arhgap10 | 1.07 | 5.08 | 1.62E-05 | 0.000109866 |
| 5 h | ENSRNOG00000013057 | Prc1 | 1.24 | 1.65 | 1.70E-05 | 0.000115169 |
| 5 h | ENSRNOG00000043366 | ENSRNOG00000043366 | -1.08 | 3.53 | 1.99E-05 | 0.000133012 |
| 5 h | ENSRNOG00000034376 | U5 | 1.65 | -0.73 | 2.46E-05 | 0.000160888 |
| 5 h | ENSRNOG00000010610 | Hpgd | -1.09 | 1.61 | 2.49E-05 | 0.00016243 |
| 5 h | ENSRNOG00000025114 | Cebpb | 1.21 | 4.97 | 2.75E-05 | 0.000177093 |
| 5 h | ENSRNOG00000017181 | Malt1 | -1.02 | 2.45 | 3.26E-05 | 0.000206652 |
| 5 h | ENSRNOG00000000827 | Ier3 | 1.22 | 0.97 | 3.34E-05 | 0.000211268 |
| 5 h | ENSRNOG00000022162 | Pbx3 | 1.20 | 2.52 | 3.61E-05 | 0.000227052 |
| 5 h | ENSRNOG00000002549 | Htr5b | -2.49 | 1.55 | 3.96E-05 | 0.000245945 |
| 5 h | ENSRNOG00000006773 | Dhrs9 | 2.96 | -1.11 | 4.18E-05 | 0.000257549 |
| 5 h | ENSRNOG00000019778 | Ptrf | 2.05 | 3.29 | 4.18E-05 | 0.000257549 |
| 5 h | ENSRNOG00000023465 | LOC500300 | 1.59 | 1.10 | 4.38E-05 | 0.000269373 |
| 5 h | ENSRNOG00000005857 | Lrrc3b | 1.44 | 2.83 | 4.46E-05 | 0.000273888 |
| 5 h | ENSRNOG00000016571 | Ngf | 1.20 | 3.62 | 5.10E-05 | 0.000308691 |
| 5 h | ENSRNOG00000036681 | D3ZXI1_RAT | -1.43 | 0.45 | 5.61E-05 | 0.00033737 |
| 5 h | ENSRNOG00000004757 | Tmem158 | -1.43 | 1.91 | 5.75E-05 | 0.000344869 |
| 5 h | ENSRNOG00000040190 | ENSRNOG00000040190 | -1.03 | 0.79 | 6.68E-05 | 0.000393959 |
| 5 h | ENSRNOG00000006324 | Trpc6 | 1.28 | 6.10 | 6.86E-05 | 0.000402747 |
| 5 h | ENSRNOG00000025198 | Gas2l3 | -1.33 | 0.47 | 7.42E-05 | 0.000432267 |
| 5 h | ENSRNOG00000009407 | Galr2 | -1.53 | 0.16 | 7.63E-05 | 0.000443291 |
| 5 h | ENSRNOG00000002475 | RGD1565798 | 1.25 | -0.10 | 7.75E-05 | 0.000449025 |
| 5 h | ENSRNOG00000032939 | LOC100360891 | -2.44 | 0.18 | 8.00E-05 | 0.000462153 |
| 5 h | ENSRNOG00000038365 | F1LXA9_RAT | 1.45 | 3.80 | 9.27E-05 | 0.000529582 |
| 5 h | ENSRNOG00000039321 | F1LZW0_RAT | -1.49 | 0.20 | 9.45E-05 | 0.000538663 |
| 5 h | ENSRNOG00000019472 | Ccbp2 | -1.40 | 0.52 | 0.000104324 | 0.000588944 |
| 5 h | ENSRNOG00000035568 | rno-mir-24-1 | 1.58 | -0.32 | 0.000122475 | 0.000677813 |
| 5 h | ENSRNOG00000009785 | CDKN3_RAT | 1.18 | -0.45 | 0.00013405 | 0.000735153 |
| 5 h | ENSRNOG00000026036 | Pdyn | 1.49 | 7.77 | 0.00014102 | 0.000767374 |
| 5 h | ENSRNOG00000011680 | Il16 | -1.05 | 6.30 | 0.000150842 | 0.000815127 |
| 5 h | ENSRNOG00000010929 | Tcam1 | -1.14 | 1.39 | 0.000154786 | 0.000833872 |
| 5 h | ENSRNOG00000029811 | Kcne2 | -1.24 | -0.25 | 0.000155296 | 0.000835879 |
| 5 h | ENSRNOG00000006674 | Fam101b | -1.11 | 2.85 | 0.000170676 | 0.000909687 |
| 5 h | ENSRNOG00000036700 | Fscn2 | -1.21 | 0.15 | 0.000174166 | 0.000924425 |
| 5 h | ENSRNOG00000043288 | LOC690806 | -1.58 | 0.34 | 0.000179552 | 0.000951211 |
| 5 h | ENSRNOG00000013304 | Arg1 | -1.41 | 4.08 | 0.00019533 | 0.001029355 |
| 5 h | ENSRNOG00000000589 | RGD1310495 | -1.05 | 1.04 | 0.000197723 | 0.001040265 |
| 5 h | ENSRNOG00000005166 | Nhlh1 | -2.16 | 3.41 | 0.000249137 | 0.001275984 |
| 5 h | ENSRNOG00000016275 | Ttr | -3.52 | 0.95 | 0.000260127 | 0.001327422 |
| 5 h | ENSRNOG00000019184 | Npr3 | 1.04 | 1.35 | 0.000285668 | 0.001438911 |
| 5 h | ENSRNOG00000016701 | Gng8 | 1.37 | -0.08 | 0.000286734 | 0.001443762 |
| 5 h | ENSRNOG00000013887 | Adra2b | 1.36 | -0.62 | 0.000325546 | 0.001618843 |
| 5 h | ENSRNOG00000021823 | Herv-frd | -1.04 | 0.00 | 0.000334536 | 0.001659425 |
| 5 h | ENSRNOG00000011923 | Osap | -1.06 | 0.17 | 0.000359705 | 0.001765538 |
| 5 h | ENSRNOG00000003354 | LOC100360473 | -1.08 | 0.29 | 0.00040248 | 0.001950233 |
| 5 h | ENSRNOG00000027385 | D3ZNJ2_RAT | -1.30 | -0.43 | 0.000420764 | 0.002026226 |
| 5 h | ENSRNOG00000002154 | Mepe | 1.28 | 0.43 | 0.000423342 | 0.002036546 |
| 5 h | ENSRNOG00000036026 | SNORD49 | 1.60 | -0.34 | 0.000463373 | 0.002198201 |
| 5 h | ENSRNOG00000035152 | SNORD113 | 1.05 | 0.94 | 0.00047364 | 0.002240087 |
| 5 h | ENSRNOG00000002053 | F1M3H3_RAT | 1.25 | 4.31 | 0.000515987 | 0.002407081 |
| 5 h | ENSRNOG00000033110 | Svep1 | -1.12 | 5.36 | 0.000564446 | 0.002607926 |
| 5 h | ENSRNOG00000024221 | LOC499465 | -1.20 | 0.16 | 0.000617807 | 0.002822908 |
| 5 h | ENSRNOG00000025287 | RGD1565611 | -1.24 | -0.03 | 0.000700409 | 0.003143515 |
| 5 h | ENSRNOG00000028662 | LOC100363455 | -1.20 | 0.17 | 0.000752922 | 0.003353949 |
| 5 h | ENSRNOG00000030542 | D4AEE8_RAT | 1.07 | -0.19 | 0.000803023 | 0.003554493 |
| 5 h | ENSRNOG00000010880 | Gpr27 | -1.61 | 0.30 | 0.000812394 | 0.003585837 |
| 5 h | ENSRNOG00000001701 | Cbr3 | 1.03 | 0.00 | 0.000820924 | 0.00362007 |
| 5 h | ENSRNOG00000016413 | Pstpip1 | 1.20 | 0.39 | 0.000875713 | 0.003830359 |
| 5 h | ENSRNOG00000034805 | SNORD113 | 1.65 | -0.62 | 0.000877467 | 0.003836832 |
| 5 h | ENSRNOG00000009881 | Fam161a | 1.04 | 0.41 | 0.000929292 | 0.004034506 |
| 5 h | ENSRNOG00000015545 | LOC685977 | -1.11 | -0.19 | 0.00099128 | 0.004270574 |
| 5 h | ENSRNOG00000006549 | LOC100362205 | 1.21 | -0.49 | 0.001010545 | 0.004336253 |
| 5 h | ENSRNOG00000028335 | Fat4 | -1.21 | 8.47 | 0.001083189 | 0.004590376 |
| 5 h | ENSRNOG00000014350 | Cyr61 | 1.29 | -0.07 | 0.001092221 | 0.004620275 |
| 5 h | ENSRNOG00000019931 | RGD1306091 | -1.05 | -0.24 | 0.00122477 | 0.005085859 |
| 5 h | ENSRNOG00000033908 | F1M377_RAT | -1.25 | -0.22 | 0.001226109 | 0.005088407 |
| 5 h | ENSRNOG00000015418 | D3ZYQ7_RAT | -1.05 | 2.64 | 0.001259391 | 0.005206496 |
| 5 h | ENSRNOG00000036927 | F1M8X4_RAT | 1.02 | -0.17 | 0.001273239 | 0.005257549 |
| 5 h | ENSRNOG00000013944 | Zc3hav1l | -1.01 | -0.10 | 0.001293255 | 0.005330778 |
| 5 h | ENSRNOG00000035909 | U2 | 1.01 | 0.03 | 0.001363228 | 0.005581473 |
| 5 h | ENSRNOG00000008611 | ENSRNOG00000008611 | 1.20 | -0.19 | 0.001484118 | 0.006018447 |
| 5 h | ENSRNOG00000028622 | Pnpla1 | 1.64 | 1.79 | 0.001518211 | 0.006131874 |
| 5 h | ENSRNOG00000013928 | Dsp | -1.87 | 3.61 | 0.001601115 | 0.006431519 |
| 5 h | ENSRNOG00000039126 | F1M8T6_RAT | 1.31 | -0.46 | 0.00183513 | 0.007249695 |
| 5 h | ENSRNOG00000023991 | Rab20 | 1.29 | -0.29 | 0.001845898 | 0.007289503 |
| 5 h | ENSRNOG00000007000 | Grhl2 | -1.13 | -0.51 | 0.001863274 | 0.00733952 |
| 5 h | ENSRNOG00000037151 | Crlf3 | -1.10 | -0.07 | 0.001878223 | 0.007381817 |
| 5 h | ENSRNOG00000000418 | Ng35 | -1.01 | 0.73 | 0.00191812 | 0.007521754 |
| 5 h | ENSRNOG00000034229 | Serpinb1b | -1.17 | 0.40 | 0.001995043 | 0.007782055 |
| 5 h | ENSRNOG00000015179 | Tradd | -1.02 | 0.19 | 0.002173485 | 0.008370992 |
| 5 h | ENSRNOG00000023633 | Crabp1 | 1.24 | -0.35 | 0.002366025 | 0.009006179 |
| 5 h | ENSRNOG00000011343 | D3ZMH4_RAT | -1.05 | 0.54 | 0.002692065 | 0.010083019 |
| 5 h | ENSRNOG00000008470 | Lrrc43 | -1.23 | -0.10 | 0.002803384 | 0.010416494 |
| 5 h | ENSRNOG00000039861 | LOC690617 | -1.49 | 0.08 | 0.003145571 | 0.011460209 |
| 5 h | ENSRNOG00000006387 | Adam4l1 | -1.13 | 0.11 | 0.003327448 | 0.012044606 |
| 5 h | ENSRNOG00000013036 | Epha8 | -1.08 | 4.72 | 0.003557582 | 0.012736271 |
| 5 h | ENSRNOG00000020446 | LOC691962 | -1.13 | -0.29 | 0.003562507 | 0.012750648 |
| 5 h | ENSRNOG00000029131 | F1LVM9_RAT | 1.14 | -0.01 | 0.004215784 | 0.014731606 |
| 5 h | ENSRNOG00000039370 | LOC100365061 | -2.05 | 0.86 | 0.005314373 | 0.017918971 |
| 5 h | ENSRNOG00000003972 | Tshr | -1.12 | 0.00 | 0.005608111 | 0.018764624 |
| 5 h | ENSRNOG00000014166 | Smoc2 | -1.46 | 1.15 | 0.006355251 | 0.020964405 |
| 5 h | ENSRNOG00000012827 | Mlf1 | 1.15 | -0.42 | 0.006759877 | 0.022068927 |
| 5 h | ENSRNOG00000011094 | Efcab6 | 1.52 | 0.69 | 0.006923264 | 0.02254643 |
| 5 h | ENSRNOG00000009735 | Fibcd1 | 1.70 | 5.15 | 0.006989498 | 0.02271992 |
| 5 h | ENSRNOG00000012155 | Sox15 | -1.17 | -0.28 | 0.007065254 | 0.022928964 |
| 5 h | ENSRNOG00000033737 | D4A3N8_RAT | -1.15 | 0.45 | 0.008682507 | 0.027323786 |
| 5 h | ENSRNOG00000009211 | C3ar1 | 1.10 | -0.23 | 0.008982383 | 0.02812862 |
| 5 h | ENSRNOG00000028484 | D4AE52_RAT | -1.26 | -0.02 | 0.009465902 | 0.029426909 |
| 5 h | ENSRNOG00000031452 | Otop3 | 1.27 | -0.50 | 0.010390327 | 0.031792178 |
| 5 h | ENSRNOG00000005246 | Tcfap2c | 1.34 | -0.39 | 0.011632852 | 0.034983375 |
| 5 h | ENSRNOG00000026060 | Arsi | -1.07 | -0.18 | 0.012574454 | 0.037350471 |
| 5 h | ENSRNOG00000024569 | Gimap9 | 1.10 | -0.70 | 0.012664105 | 0.037505544 |
| 5 h | ENSRNOG00000019842 | Artn | -1.28 | -0.46 | 0.014151312 | 0.041188012 |
| 5 h | ENSRNOG00000035126 | SNORD113 | 1.28 | -0.59 | 0.016555194 | 0.047032716 |

**Table S4.** Differentially expressed lncRNAs

| Condition | LncRNA | logFC | logCPM | PValue | FDR |
| --- | --- | --- | --- | --- | --- |
| 30 min | XLOC_031897 | 7.19 | 5.63 | 2.87E-26 | 2.95E-22 |
| 30 min | XLOC_115497 | 2.44 | 7.58 | 1.63E-11 | 8.34E-08 |
| 30 min | XLOC_011857 | 3.12 | 4.08 | 4.48E-07 | 0.001532898 |
| 30 min | XLOC_051598 | 1.47 | 6.66 | 1.17E-06 | 0.003011844 |
| 30 min | XLOC_120346 | 1.45 | 5.57 | 2.94E-06 | 0.006035458 |
| 2 h | XLOC_157345 | 3.91 | 6.40 | 6.47E-40 | 6.63E-36 |
| 2 h | XLOC_115497 | 4.49 | 7.58 | 2.67E-37 | 1.37E-33 |
| 2 h | XLOC_031897 | 7.75 | 5.63 | 1.98E-32 | 6.75E-29 |
| 2 h | XLOC_130355 | 3.23 | 7.17 | 1.63E-31 | 4.18E-28 |
| 2 h | XLOC_051598 | 2.28 | 6.66 | 6.18E-17 | 1.27E-13 |
| 2 h | XLOC_110977 | 1.93 | 6.89 | 4.07E-13 | 6.96E-10 |
| 2 h | XLOC_157825 | 3.42 | 7.66 | 1.97E-12 | 2.88E-09 |
| 2 h | XLOC_055591 | 5.57 | 4.54 | 5.39E-12 | 6.52E-09 |
| 2 h | XLOC_035001 | 2.09 | 7.19 | 5.72E-12 | 6.52E-09 |
| 2 h | XLOC_094324 | 2.25 | 6.83 | 2.21E-11 | 2.27E-08 |
| 2 h | XLOC_050251 | 2.20 | 6.53 | 4.60E-11 | 4.29E-08 |
| 2 h | XLOC_146390 | 2.18 | 8.53 | 6.35E-11 | 5.43E-08 |
| 2 h | XLOC_155437 | 1.74 | 7.80 | 6.35E-10 | 5.01E-07 |
| 2 h | XLOC_045589 | 1.93 | 9.56 | 7.66E-07 | 0.000561491 |
| 2 h | XLOC_092630 | 2.28 | 5.33 | 9.81E-06 | 0.006289621 |
| 2 h | XLOC_017742 | 1.22 | 6.44 | 1.64E-05 | 0.009602414 |
| 2 h | XLOC_101933 | 1.45 | 6.82 | 1.69E-05 | 0.009602414 |
| 2 h | XLOC_076716 | 1.68 | 5.00 | 2.33E-05 | 0.012570913 |
| 2 h | XLOC_096893 | 1.46 | 6.54 | 2.86E-05 | 0.014015921 |
| 2 h | XLOC_026193 | 1.09 | 8.91 | 3.01E-05 | 0.014015921 |
| 2 h | XLOC_128886 | 3.39 | 3.19 | 0.000104114 | 0.03813555 |
| 2 h | XLOC_069761 | -2.45 | 5.26 | 0.0001165 | 0.041200808 |
| 2 h | XLOC_003451 | 2.80 | 3.75 | 0.00013168 | 0.045016891 |
| 5 h | XLOC_115497 | 4.71 | 7.58 | 6.05E-45 | 6.21E-41 |
| 5 h | XLOC_130355 | 3.29 | 7.17 | 2.54E-37 | 1.30E-33 |
| 5 h | XLOC_146390 | 4.17 | 8.53 | 4.59E-34 | 1.57E-30 |
| 5 h | XLOC_055591 | 6.41 | 4.54 | 1.05E-28 | 2.69E-25 |
| 5 h | XLOC_134395 | 4.02 | 4.97 | 2.79E-24 | 5.71E-21 |
| 5 h | XLOC_094324 | 3.14 | 6.83 | 4.32E-23 | 7.38E-20 |
| 5 h | XLOC_110977 | 2.27 | 6.89 | 7.99E-21 | 1.17E-17 |
| 5 h | XLOC_153554 | 3.96 | 4.64 | 3.34E-19 | 4.29E-16 |
| 5 h | XLOC_051598 | 2.41 | 6.66 | 3.85E-19 | 4.39E-16 |
| 5 h | XLOC_048557 | 3.26 | 6.50 | 3.15E-18 | 3.23E-15 |
| 5 h | XLOC_155437 | 2.38 | 7.80 | 3.40E-17 | 3.17E-14 |
| 5 h | XLOC_112065 | 3.71 | 7.50 | 2.28E-16 | 1.95E-13 |
| 5 h | XLOC_157825 | 3.90 | 7.66 | 9.61E-16 | 7.59E-13 |
| 5 h | XLOC_003451 | 4.70 | 3.75 | 7.41E-15 | 5.43E-12 |
| 5 h | XLOC_157345 | 2.13 | 6.40 | 2.38E-14 | 1.63E-11 |
| 5 h | XLOC_059611 | 2.38 | 6.73 | 3.19E-13 | 2.05E-10 |
| 5 h | XLOC_035001 | 1.93 | 7.19 | 5.59E-12 | 3.37E-09 |
| 5 h | XLOC_112734 | 7.67 | 3.46 | 9.34E-12 | 5.32E-09 |
| 5 h | XLOC_084066 | 3.74 | 3.49 | 1.32E-10 | 7.13E-08 |
| 5 h | XLOC_031897 | 4.28 | 5.63 | 3.63E-10 | 1.86E-07 |
| 5 h | XLOC_140626 | 3.14 | 3.98 | 6.99E-10 | 3.41E-07 |
| 5 h | XLOC_164782 | 3.30 | 4.45 | 8.86E-10 | 4.13E-07 |
| 5 h | XLOC_101383 | -1.11 | 8.06 | 1.60E-08 | 7.14E-06 |
| 5 h | XLOC_049808 | 1.40 | 6.30 | 2.22E-08 | 9.48E-06 |
| 5 h | XLOC_047519 | 1.97 | 8.13 | 2.33E-08 | 9.55E-06 |
| 5 h | XLOC_112155 | -2.11 | 6.67 | 2.69E-08 | 1.06E-05 |
| 5 h | XLOC_067977 | 3.85 | 3.46 | 6.67E-08 | 2.53E-05 |
| 5 h | XLOC_171906 | 7.13 | 3.00 | 8.25E-08 | 3.00E-05 |
| 5 h | XLOC_045589 | 2.10 | 9.56 | 8.47E-08 | 3.00E-05 |
| 5 h | XLOC_155643 | -2.28 | 5.01 | 1.01E-07 | 3.47E-05 |
| 5 h | XLOC_139362 | 2.43 | 9.03 | 1.45E-07 | 4.70E-05 |
| 5 h | XLOC_071664 | -2.25 | 6.95 | 1.47E-07 | 4.70E-05 |
| 5 h | XLOC_018198 | -1.36 | 7.02 | 3.06E-07 | 9.51E-05 |
| 5 h | XLOC_140619 | 1.13 | 7.42 | 3.44E-07 | 0.000103639 |
| 5 h | XLOC_050251 | 1.56 | 6.53 | 4.75E-07 | 0.000139106 |
| 5 h | XLOC_165879 | 1.73 | 6.23 | 5.71E-07 | 0.000162791 |
| 5 h | XLOC_069948 | -2.12 | 8.40 | 9.51E-07 | 0.000263493 |
| 5 h | XLOC_117240 | 1.10 | 9.56 | 1.25E-06 | 0.000336125 |
| 5 h | XLOC_148305 | 1.37 | 6.59 | 1.29E-06 | 0.000339096 |
| 5 h | XLOC_155278 | 1.02 | 8.73 | 1.32E-06 | 0.000339401 |
| 5 h | XLOC_047831 | 1.99 | 5.46 | 1.44E-06 | 0.00035944 |
| 5 h | XLOC_126330 | -2.18 | 5.82 | 2.36E-06 | 0.000575912 |
| 5 h | XLOC_069899 | 2.57 | 3.57 | 4.56E-06 | 0.001087827 |
| 5 h | XLOC_092616 | 1.69 | 5.68 | 5.02E-06 | 0.001170691 |
| 5 h | XLOC_058725 | -1.06 | 9.15 | 5.37E-06 | 0.001224942 |
| 5 h | XLOC_021010 | 1.91 | 6.67 | 1.09E-05 | 0.002375415 |
| 5 h | XLOC_037584 | -1.29 | 7.54 | 1.40E-05 | 0.002992456 |
| 5 h | XLOC_124463 | -4.09 | 3.76 | 1.85E-05 | 0.003865182 |
| 5 h | XLOC_029633 | 1.71 | 5.82 | 1.92E-05 | 0.003944351 |
| 5 h | XLOC_071661 | -2.28 | 5.83 | 3.70E-05 | 0.00743671 |
| 5 h | XLOC_128886 | 3.54 | 3.19 | 3.95E-05 | 0.007786604 |
| 5 h | XLOC_052581 | -2.23 | 7.14 | 5.99E-05 | 0.011378497 |
| 5 h | XLOC_092630 | 2.08 | 5.33 | 7.49E-05 | 0.013961321 |
| 5 h | XLOC_167955 | -1.40 | 7.07 | 8.04E-05 | 0.014731793 |
| 5 h | XLOC_012719 | 1.14 | 8.40 | 8.69E-05 | 0.015370484 |
| 5 h | XLOC_105393 | -1.31 | 10.29 | 9.18E-05 | 0.015763231 |
| 5 h | XLOC_012720 | 1.24 | 7.60 | 9.22E-05 | 0.015763231 |
| 5 h | XLOC_135543 | -2.10 | 4.42 | 9.45E-05 | 0.015894073 |
| 5 h | XLOC_003921 | -1.05 | 9.47 | 0.000102856 | 0.017014384 |
| 5 h | XLOC_150877 | 5.54 | 2.49 | 0.000111744 | 0.018191217 |
| 5 h | XLOC_058341 | -1.79 | 5.86 | 0.0001418 | 0.022373811 |
| 5 h | XLOC_155598 | -1.14 | 6.85 | 0.000147821 | 0.022970518 |
| 5 h | XLOC_022015 | 2.81 | 4.31 | 0.00026031 | 0.039260896 |
| 5 h | XLOC_096893 | 1.13 | 6.54 | 0.000295115 | 0.043238593 |

**Table S5. Differentially expressed repeat types**

| Condition | repType | logFC | logCPM | PValue | FDR |
| --- | --- | --- | --- | --- | --- |
| 30 min | Simple_repeat:Simple_repeat:(TCCCG)n | 6.49 | 6.01 | 5.69E-65 | 3.41E-62 |
| 30 min | Simple_repeat:Simple_repeat:(CCCCG)n | 1.87 | 6.68 | 1.74E-10 | 3.49E-08 |
| 30 min | Simple_repeat:Simple_repeat:(TTTTTA)n | 1.57 | 6.31 | 1.73E-10 | 3.49E-08 |
| 30 min | Simple_repeat:Simple_repeat:(TTCG)n | 2.25 | 3.99 | 1.31E-09 | 1.75E-07 |
| 30 min | LTR:ERV1:MER21B | 0.73 | 9.86 | 1.58E-09 | 1.75E-07 |
| 30 min | DNA:MER2_type:MER82 | 1.42 | 6.49 | 1.75E-09 | 1.75E-07 |
| 30 min | tRNA:tRNA:tRNA-Ser-TCG | 1.27 | 7.12 | 4.70E-09 | 4.03E-07 |
| 30 min | LTR:ERV1:LTR9 | 0.94 | 7.02 | 6.94E-09 | 5.21E-07 |
| 30 min | LTR:MaLR:MTE2b-int | -0.77 | 8.13 | 4.59E-08 | 3.06E-06 |
| 30 min | DNA:MER1_type:Charlie11 | -1.36 | 5.67 | 9.41E-07 | 5.64E-05 |
| 30 min | tRNA:tRNA:tRNA-His-CAY_ | 1.36 | 5.00 | 3.05E-06 | 0.000166094 |
| 30 min | LTR:MaLR:MLT1J2 | 0.99 | 5.90 | 8.89E-06 | 0.000444531 |
| 30 min | Low_complexity:Low_complexity:C-rich | 0.39 | 12.00 | 0.000130058 | 0.006002696 |
| 30 min | LINE:L1:L1MC1 | 0.83 | 6.65 | 0.000142129 | 0.006091224 |
| 30 min | LTR:MaLR:ORR1D1 | 0.53 | 7.80 | 0.000152605 | 0.006104185 |
| 30 min | Unknown:Unknown:MurSatRep1 | -0.30 | 12.38 | 0.0002362 | 0.0088575 |
| 30 min | tRNA:tRNA:tRNA-Ser-TCY | 1.12 | 4.24 | 0.000334995 | 0.011823352 |
| 30 min | LTR:ERVK:RMER3D1 | 0.60 | 7.40 | 0.000602046 | 0.020068186 |
| 30 min | Simple_repeat:Simple_repeat:(CAAAA)n | 0.31 | 10.13 | 0.000891284 | 0.028145811 |
| 30 min | LTR:MaLR:ORR1A_Rn | -0.31 | 9.70 | 0.000968175 | 0.029045238 |
| 2 h | Simple_repeat:Simple_repeat:(TCCCG)n | 6.09 | 6.01 | 8.04E-69 | 4.82E-66 |
| 2 h | tRNA:tRNA:tRNA-Ser-TCG | 2.47 | 7.12 | 1.32E-27 | 3.97E-25 |
| 2 h | LTR:ERV1:MER21B | 1.05 | 9.86 | 3.03E-18 | 6.05E-16 |
| 2 h | LTR:ERV1:LTR9 | 1.34 | 7.02 | 1.15E-17 | 1.72E-15 |
| 2 h | Simple_repeat:Simple_repeat:(CCCCG)n | 2.30 | 6.68 | 1.66E-14 | 2.00E-12 |
| 2 h | LINE:L1:L1MD3 | 0.85 | 10.00 | 6.17E-14 | 6.17E-12 |
| 2 h | tRNA:tRNA:tRNA-Arg-AGG | 2.51 | 4.43 | 9.95E-13 | 8.53E-11 |
| 2 h | Simple_repeat:Simple_repeat:(TTTA)n | 0.95 | 10.00 | 4.91E-12 | 3.68E-10 |
| 2 h | Simple_repeat:Simple_repeat:(CTAGGG)n | 0.89 | 7.47 | 5.81E-11 | 3.87E-09 |
| 2 h | tRNA:tRNA:tRNA-His-CAY_ | 1.71 | 5.00 | 1.91E-09 | 1.14E-07 |
| 2 h | LTR:MaLR:ORR1A_Rn | -0.54 | 9.70 | 1.29E-08 | 7.02E-07 |
| 2 h | SINE:ID:ID_Rn2 | 0.42 | 12.51 | 2.37E-08 | 1.19E-06 |
| 2 h | tRNA:tRNA:tRNA-Cys-TGY | 1.62 | 4.86 | 3.92E-08 | 1.81E-06 |
| 2 h | Simple_repeat:Simple_repeat:(CAT)n | -0.87 | 7.83 | 7.10E-08 | 3.04E-06 |
| 2 h | LTR:MaLR:MLT1A | 0.77 | 7.64 | 1.20E-07 | 4.80E-06 |
| 2 h | tRNA:tRNA:tRNA-Ser-TCY | 1.56 | 4.24 | 5.37E-07 | 1.90E-05 |
| 2 h | tRNA:tRNA:tRNA-Trp-TGG | 1.72 | 3.85 | 5.38E-07 | 1.90E-05 |
| 2 h | DNA:Tip100:MER91B | 1.70 | 4.73 | 7.09E-07 | 2.36E-05 |
| 2 h | tRNA:tRNA:tRNA-Glu-GAG_ | 1.49 | 5.81 | 1.30E-06 | 4.10E-05 |
| 2 h | LINE:L1:L1ME3A | 0.83 | 6.44 | 7.63E-06 | 0.00022899 |
| 2 h | tRNA:tRNA:tRNA-Lys-AAA | 1.54 | 3.85 | 9.55E-06 | 0.000272822 |
| 2 h | tRNA:tRNA:tRNA-Asn-AAC | 1.87 | 3.93 | 1.15E-05 | 0.000314304 |
| 2 h | DNA:Mariner:MADE2 | 0.64 | 7.69 | 1.37E-05 | 0.00035713 |
| 2 h | LTR:ERVK:RMER3D1 | 0.73 | 7.40 | 2.91E-05 | 0.000726516 |
| 2 h | Simple_repeat:Simple_repeat:(CAAAA)n | 0.37 | 10.13 | 4.24E-05 | 0.001016822 |
| 2 h | LTR:ERVK:RLTR20D | 1.54 | 3.91 | 5.81E-05 | 0.001341023 |
| 2 h | DNA:MER1_type:Charlie11 | -0.97 | 5.67 | 6.95E-05 | 0.001544567 |
| 2 h | tRNA:tRNA:tRNA-Tyr-TAC | 1.59 | 4.00 | 8.57E-05 | 0.001835619 |
| 2 h | LTR:ERVL:MER74B | -1.13 | 4.91 | 9.33E-05 | 0.001929726 |
| 2 h | DNA:MER1_type:MER5A1 | 0.50 | 8.32 | 0.000226235 | 0.004524694 |
| 2 h | Simple_repeat:Simple_repeat:(A)n | 0.34 | 14.33 | 0.000260521 | 0.005042346 |
| 2 h | LTR:ERV1:RLTR23 | -0.82 | 6.06 | 0.000334392 | 0.006269856 |
| 2 h | LTR:ERVK:RMER19A | -0.57 | 7.01 | 0.000436445 | 0.007935369 |
| 2 h | LTR:MaLR:MTE2b-int | -0.48 | 8.13 | 0.000450834 | 0.007955886 |
| 2 h | snRNA:snRNA:U2 | 0.53 | 6.75 | 0.000611824 | 0.010488403 |
| 2 h | LTR:ERV1:RMER21B | -0.89 | 5.22 | 0.000644014 | 0.010733574 |
| 2 h | LINE:L1:Lx2A1 | -1.18 | 5.33 | 0.000760354 | 0.012330061 |
| 2 h | LTR:ERV1:LTR65 | 1.12 | 4.45 | 0.000896979 | 0.014162825 |
| 2 h | Simple_repeat:Simple_repeat:(CAAAAA)n | 0.39 | 11.06 | 0.000923608 | 0.014209356 |
| 2 h | Unknown:Unknown:MurSatRep1 | -0.27 | 12.38 | 0.00097181 | 0.014577151 |
| 2 h | LTR:MaLR:ORR1C2 | -0.40 | 8.12 | 0.001160423 | 0.016981797 |
| 2 h | LTR:ERVL:RLTR28B | -0.48 | 7.70 | 0.001197887 | 0.017112676 |
| 2 h | DNA:MER1_type:Charlie10 | -0.48 | 7.29 | 0.001545955 | 0.02157146 |
| 2 h | LTR:ERVK:RLTR31_Rn2 | -0.47 | 8.08 | 0.00195207 | 0.026619141 |
| 2 h | LTR:ERV1:LTR48B | 1.14 | 4.27 | 0.002075054 | 0.027667391 |
| 2 h | Simple_repeat:Simple_repeat:(CATATA)n | 0.63 | 6.81 | 0.002625587 | 0.033518137 |
| 2 h | Simple_repeat:Simple_repeat:(TTCG)n | 1.33 | 3.99 | 0.002581427 | 0.033518137 |
| 2 h | LINE:L1:L1MB4 | -0.58 | 6.95 | 0.002975276 | 0.037190952 |
| 2 h | LTR:MaLR:MTE-int | -0.69 | 5.50 | 0.003257473 | 0.039887424 |
| 2 h | LTR:ERVK:RMER3-int | -0.59 | 6.39 | 0.00393589 | 0.047230686 |
| 5 h | Simple_repeat:Simple_repeat:(TCCCG)n | 6.85 | 6.01 | 7.44E-75 | 4.46E-72 |
| 5 h | Simple_repeat:Simple_repeat:(CAAG)n | 1.51 | 7.88 | 9.43E-37 | 2.83E-34 |
| 5 h | LTR:ERV1:MER21B | 1.13 | 9.86 | 1.82E-21 | 3.65E-19 |
| 5 h | DNA:MER1_type:MER113 | 3.91 | 3.04 | 3.29E-20 | 4.93E-18 |
| 5 h | DNA:MER1_type:Charlie4a | 1.57 | 6.20 | 9.47E-20 | 1.14E-17 |
| 5 h | LTR:ERV1:LTR9 | 1.24 | 7.02 | 1.10E-17 | 1.10E-15 |
| 5 h | Simple_repeat:Simple_repeat:(CCCCG)n | 2.35 | 6.68 | 6.23E-17 | 5.34E-15 |
| 5 h | LTR:ERVK:RMER17D2 | 2.17 | 4.49 | 1.32E-16 | 9.91E-15 |
| 5 h | LTR:ERVL:MER68 | 1.03 | 7.55 | 1.63E-15 | 1.09E-13 |
| 5 h | LTR:MaLR:MTB_Rn-int | 1.57 | 5.79 | 8.96E-13 | 5.37E-11 |
| 5 h | tRNA:tRNA:tRNA-Arg-AGG | 1.86 | 4.43 | 3.65E-11 | 1.99E-09 |
| 5 h | tRNA:tRNA:tRNA-Cys-TGY | 1.62 | 4.86 | 2.11E-10 | 1.05E-08 |
| 5 h | LINE:L1:L1MD3 | 0.71 | 10.00 | 2.85E-10 | 1.22E-08 |
| 5 h | tRNA:tRNA:tRNA-His-CAY_ | 1.66 | 5.00 | 2.78E-10 | 1.22E-08 |
| 5 h | tRNA:tRNA:tRNA-Ser-TCG | 1.32 | 7.12 | 3.96E-10 | 1.58E-08 |
| 5 h | LTR:ERVK:RLTR25A | 1.13 | 6.36 | 4.88E-10 | 1.83E-08 |
| 5 h | Simple_repeat:Simple_repeat:(TTTA)n | 0.84 | 10.00 | 7.86E-10 | 2.78E-08 |
| 5 h | LTR:ERV1:LTR65 | 2.23 | 4.45 | 1.11E-09 | 3.68E-08 |
| 5 h | LTR:ERVL:LTR16A | 1.41 | 7.79 | 1.33E-09 | 4.21E-08 |
| 5 h | tRNA:tRNA:tRNA-Glu-GAG_ | 1.78 | 5.81 | 1.53E-09 | 4.59E-08 |
| 5 h | Simple_repeat:Simple_repeat:(CAT)n | -0.92 | 7.83 | 5.05E-09 | 1.44E-07 |
| 5 h | Simple_repeat:Simple_repeat:(CAAAA)n | 0.50 | 10.13 | 2.67E-08 | 7.28E-07 |
| 5 h | LTR:ERV1:MER50B | 1.38 | 6.98 | 8.22E-08 | 2.08E-06 |
| 5 h | tRNA:tRNA:tRNA-Trp-TGG | 1.86 | 3.85 | 8.34E-08 | 2.08E-06 |
| 5 h | LTR:MaLR:ORR1A_Rn | -0.47 | 9.70 | 3.88E-07 | 9.30E-06 |
| 5 h | tRNA:tRNA:tRNA-Lys-AAA | 1.76 | 3.85 | 4.48E-07 | 1.03E-05 |
| 5 h | DNA:AcHobo:MER63D | 1.23 | 5.00 | 4.88E-07 | 1.08E-05 |
| 5 h | Simple_repeat:Simple_repeat:(CTAGGG)n | 0.64 | 7.47 | 6.15E-07 | 1.32E-05 |
| 5 h | Simple_repeat:Simple_repeat:(TTCA)n | 0.64 | 7.85 | 8.27E-07 | 1.71E-05 |
| 5 h | tRNA:tRNA:tRNA-Tyr-TAC | 1.59 | 4.00 | 2.94E-06 | 5.89E-05 |
| 5 h | snRNA:snRNA:U2 | 0.68 | 6.75 | 3.19E-06 | 6.17E-05 |
| 5 h | Simple_repeat:Simple_repeat:(CAAGG)n | 1.06 | 4.98 | 5.89E-06 | 0.000107149 |
| 5 h | LTR:ERVL:LTR40c | 0.89 | 6.04 | 5.86E-06 | 0.000107149 |
| 5 h | DNA:AcHobo:MER63B | -1.56 | 3.89 | 6.66E-06 | 0.000117479 |
| 5 h | LTR:ERVL:RLTR28B | -0.62 | 7.70 | 1.90E-05 | 0.000326474 |
| 5 h | tRNA:tRNA:tRNA-Ser-TCY | 1.23 | 4.24 | 4.76E-05 | 0.000792942 |
| 5 h | DNA:MER1_type:MER58 | -0.42 | 9.62 | 0.000100328 | 0.001626944 |
| 5 h | DNA:Mariner:MADE2 | 0.52 | 7.69 | 0.000125598 | 0.001983123 |
| 5 h | LINE:L1:L1MB4 | -0.72 | 6.95 | 0.000148653 | 0.002286969 |
| 5 h | SINE:ID:ID_Rn2 | 0.27 | 12.51 | 0.000220771 | 0.003311564 |
| 5 h | LTR:ERVK:RLTR18 | 0.40 | 8.74 | 0.000228722 | 0.003347144 |
| 5 h | Simple_repeat:Simple_repeat:(CAGAA)n | 0.64 | 6.36 | 0.000247966 | 0.003534119 |
| 5 h | Simple_repeat:Simple_repeat:(TAAAAA)n | -1.11 | 4.72 | 0.000253279 | 0.003534119 |
| 5 h | LTR:MaLR:MTEb-int | 1.26 | 4.39 | 0.000284626 | 0.003795017 |
| 5 h | LTR:MaLR:ORR1A_Rn-int | 0.77 | 8.79 | 0.000280028 | 0.003795017 |
| 5 h | LINE:L1:L1_Rod | 0.48 | 9.81 | 0.000315773 | 0.004118776 |
| 5 h | LTR:ERVK:RMER3B | -0.38 | 9.33 | 0.000335066 | 0.004277438 |
| 5 h | Simple_repeat:Simple_repeat:(CCGCG)n | -0.96 | 6.37 | 0.000388128 | 0.0048516 |
| 5 h | DNA:MER1_type:Charlie11 | 0.67 | 5.67 | 0.00040473 | 0.004955873 |
| 5 h | Simple_repeat:Simple_repeat:(TTTTTG)n | 0.32 | 10.57 | 0.000432672 | 0.005090259 |
| 5 h | LTR:ERV1:RMER21B | -0.92 | 5.22 | 0.000429269 | 0.005090259 |
| 5 h | LINE:CR1:L3 | 0.26 | 12.58 | 0.000617838 | 0.0071289 |
| 5 h | DNA:AcHobo:MER63 | -0.44 | 8.08 | 0.000653349 | 0.007396399 |
| 5 h | Simple_repeat:Simple_repeat:(CCCCCT)n | 0.51 | 8.53 | 0.000833338 | 0.009259306 |
| 5 h | LINE:L1:L1M4c | -1.32 | 5.69 | 0.000881064 | 0.009611609 |
| 5 h | tRNA:tRNA:tRNA-Arg-CGY_ | 0.86 | 4.57 | 0.001185021 | 0.01269665 |
| 5 h | DNA:MER1_type:MER5A1 | 0.43 | 8.32 | 0.001309929 | 0.013788729 |
| 5 h | DNA:MER1_type:Charlie10 | -0.46 | 7.29 | 0.001367512 | 0.014146673 |
| 5 h | DNA:Tip100:MER91B | 1.07 | 4.73 | 0.001408593 | 0.014324678 |
| 5 h | DNA:MER1_type:MER58A | 0.34 | 8.73 | 0.001648366 | 0.016483665 |
| 5 h | LINE:L1:L1_Mur2 | -0.41 | 9.95 | 0.001733248 | 0.016790453 |
| 5 h | LTR:ERVK:RLTR33 | -0.41 | 7.34 | 0.001735013 | 0.016790453 |
| 5 h | LTR:ERV1:MER92B | 0.79 | 5.26 | 0.001828335 | 0.017385944 |
| 5 h | LTR:ERV1:RNLTR16 | -0.53 | 6.97 | 0.001854501 | 0.017385944 |
| 5 h | Simple_repeat:Simple_repeat:(CTA)n | 0.49 | 6.42 | 0.002189846 | 0.020194073 |
| 5 h | tRNA:tRNA:tRNA-Asn-AAC | 1.26 | 3.93 | 0.002221348 | 0.020194073 |
| 5 h | Simple_repeat:Simple_repeat:(CGTG)n | 0.36 | 8.50 | 0.003409207 | 0.030530211 |
| 5 h | LTR:ERV1:LTR48 | 0.71 | 4.73 | 0.003669271 | 0.032375925 |
| 5 h | LTR:MaLR:MLT1C | -0.38 | 8.47 | 0.003872617 | 0.03367493 |
| 5 h | LINE:L1:L1ME3A | 0.54 | 6.44 | 0.00398206 | 0.034131942 |
| 5 h | LINE:L1:RNHAL1 | 0.34 | 8.03 | 0.004177204 | 0.035300317 |
| 5 h | Simple_repeat:Simple_repeat:(TGG)n | 0.35 | 9.56 | 0.005398382 | 0.044986515 |
| 5 h | LTR:ERVL:MT2B | 0.26 | 10.98 | 0.005872122 | 0.048264016 |
| 5 h | Simple_repeat:Simple_repeat:(CCCGG)n | -0.60 | 6.71 | 0.006145755 | 0.048763047 |
| 5 h | Simple_repeat:Simple_repeat:(TTCC)n | 0.27 | 9.78 | 0.006176653 | 0.048763047 |
| 5 h | LTR:MaLR:MLT1E1 | 0.37 | 7.47 | 0.006066315 | 0.048763047 |
